# Cyanine‐Flavonol Hybrids for Near‐Infrared Light‐Activated Delivery of Carbon Monoxide

**DOI:** 10.1002/chem.202003272

**Published:** 2020-09-04

**Authors:** Lenka Štacková, Marina Russo, Lucie Muchová, Vojtěch Orel, Libor Vítek, Peter Štacko, Petr Klán

**Affiliations:** ^1^ Department of Chemistry and RECETOX Faculty of Science Masaryk University Kamenice 5 62500 Brno Czech Republic; ^2^ Institute of Medical Biochemistry and Laboratory Diagnostics General Faculty Hospital and 1st Faculty of Medicine Charles University Na Bojišti 3 12108 Praha 2 Czech Republic

**Keywords:** CO release, cyanine, near-infrared light, photoCORM, photorelease

## Abstract

Carbon monoxide (CO) is an endogenous signaling molecule that controls a number of physiological processes. To circumvent the inherent toxicity of CO, light‐activated CO‐releasing molecules (photoCORMs) have emerged as an alternative for its administration. However, their wider application requires photoactivation using biologically benign visible and near‐infrared (NIR) light. In this work, a strategy to access such photoCORMs by fusing two CO‐releasing flavonol moieties with a NIR‐absorbing cyanine dye is presented. These hybrids liberate two molecules of CO in high chemical yields upon activation with NIR light up to 820 nm and exhibit excellent uncaging cross‐sections, which surpass the state‐of‐the‐art by two orders of magnitude. Furthermore, the biocompatibility and applicability of the system in vitro and in vivo are demonstrated, and a mechanism of CO release is proposed. It is hoped that this strategy will stimulate the discovery of new classes of photoCORMs and accelerate the translation of CO‐based phototherapy into practice.

## Graphical abstract

**CO worker**: Carbon monoxide (CO) is a signaling molecule with potent therapeutic properties hampered by its inherent toxicity. Here, cross‐breeding a CO‐releasing flavonol moiety with heptamethine cyanine dye leads to highly efficient transition‐metal‐free photoCORMs (CO‐releasing molecules). These photoCORMs operate in the middle of the phototherapeutic window, release two molecules of CO upon irradiation, and are shown to be applicable in vivo.

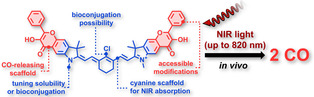

## Introduction

Use of light as a control stimulus offers unparalleled advantages in terms of availability, adjustability, high spatial and temporal precision, high orthogonality towards biochemical systems, and the minimization of waste products. The irradiation wavelengths applicable in living organisms are restricted by the adverse effects of UV‐ and visible‐light absorption and optical scattering by endogenous chromophores, which limits the depth of tissue penetration.^1^, ^2^ Successful application of photodynamic therapy (PDT) lends credibility to the use of light in the near‐infrared (NIR) range of 650–900 nm, known as the phototherapeutic window, in clinical applications.^3^

Carbon monoxide (CO) has recently emerged as a promising target for photodelivery, possessing remarkable therapeutic potential.^4^, ^5^, ^6^, ^7^ It is a naturally occurring cell‐signaling molecule exhibiting strong cytoprotective, cardioprotective, anti‐inflammatory, and anti‐microbial effects at submicromolar concentrations.^8^ Taking advantage of an anti‐Warburg effect,^9^ cancer cells and tumors exposed to CO are forced to switch to oxidative metabolism, leading eventually to growth inhibition, cellular exhaustion, and death.^10^ Moreover, the sensitivity of cancer cells towards chemotherapeutics is significantly increased (up to 1000‐fold) upon exposure to CO while simultaneously protecting normal cell growth and viability. CO also exhibits potent multifactorial inhibitory and anti‐angiogenic effects on cancer proliferation.^11^, ^12^

**Figure 1:**
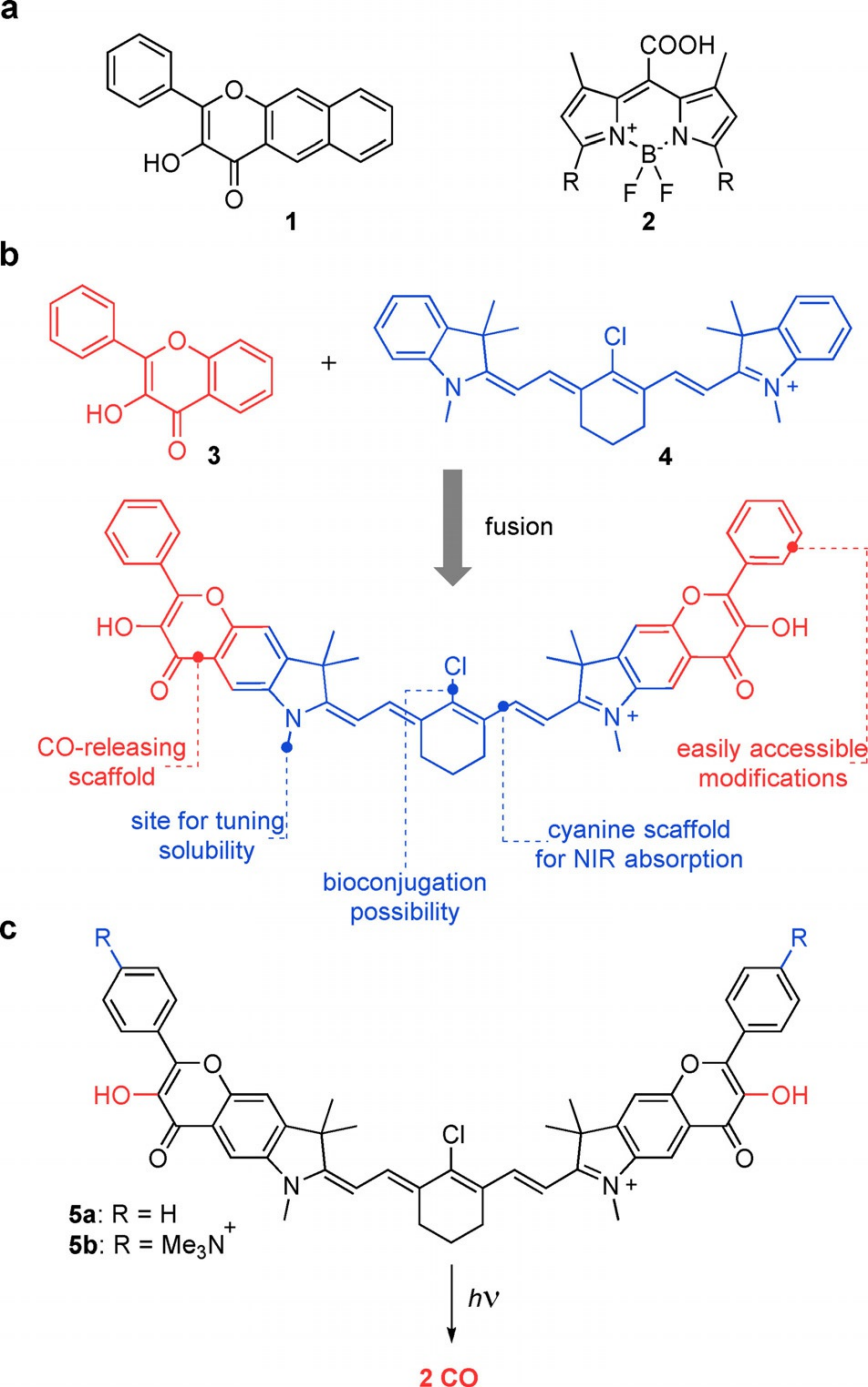
(a) Flavonol‐based **1** (left) and BODIPY‐based **2** (right) photoCORMs. (b) The concept of fusing a flavonol photoCORM moiety and a heptamethine cyanine dye into the conjugated system proposed in this work. Structural elements suitable for further modifications are depicted. (c) Photochemical release of CO from cyanine‐flavonol hybrids **5 a**–**b** prepared and studied herein. Trimethylammonium groups installed in **5 b** facilitate solubility in aqueous media. Counter anions are omitted for clarity.

However, owing to its inherent toxicity and arduous administration of CO, many CO‐releasing molecules (CORMs), primarily based on transition metal‐carbonyl complexes, have been developed.^4^, ^13^, ^14^ Generally, these CORMs rely on enzymes as the release triggers or on a solvent‐mediated ligand exchange reaction in aqueous media, methods that exhibit poor spatial and temporal control over the release profile.^15^, ^16^ Furthermore, the metal backbone left upon CO release from these metal‐carbonyl complexes can lead to uncontrolled reactions with adjacent cells, resulting in their damage and a major barrier to in vivo CORM applications.^6^ Transition‐metal‐free light‐triggered CORMs (photoCORMs) have recently appeared with the promise of circumventing these challenges. Many organic molecules, such as cyclopropenones,^17^, ^18^, ^19^ 1,3‐cyclobutadiones,^20^ or 1,2‐dioxolane‐3,4‐diones,^21^ liberate CO upon irradiation with biologically adverse UV light. Cyclic aromatic α‐diketones^22^ (*λ*_max_=468 nm) and a xanthene‐based carboxylic acid^23^ (*λ*_max_=488 nm) have been designed to undergo photochemical decarbonylation with visible light. Recently, a class of π‐extended flavonols **1** based on a naturally occurring flavone scaffold has been shown to efficiently release CO upon irradiation at 400–540 nm (Figure 1 a, left),^24^, ^25^, ^26^, ^27^ and the effects of liberated CO on the cells were unequivocally demonstrated.^28^ Our recent mechanistic investigation of this system revealed the existence of three orthogonal pathways for the CO photorelease.^29^ *meso*‐Carboxy BODIPY derivative **2** is currently the only organic photoCORM operating at the edge of a phototherapeutic window (*λ*_max_=652 nm), but it suffers from a low uncaging cross‐section *Φϵ*_max_ (Figure 1 a, right).^30^

## Results and Discussion

After elucidating the mechanism of photochemical CO release from flavonol **1**,^29^ we envisioned that fusing its analog **3** with an established chromophore could serve as a general strategy for designing new classes of photoCORMs, especially those absorbing in the NIR region (Figure 1 b). Heptamethine cyanine (Cy7) chromophore **4** was chosen for its strong absorption in the center of the phototherapeutic window, synthetic versatility, and its well‐established role in contemporary chemistry, biology, and medicine.^31^, ^32^, ^33^, ^34^ For instance, the most prominent cyanine dye, indocyanine green, is FDA approved and currently enrolled in hundreds of clinical trials despite being discovered over 60 years ago.^35^ The symmetric nature of cyanines also allows the installation of two flavonol moieties in the molecule, facilitating a potential release of two equivalents of CO (Figure 1 c). In addition, such a system exhibits high customization potential for further applications owing to the presence of several versatile structural elements. The *N*‐substituents of the indolenine heterocycles can be used to tune its solubility in aqueous media or for bioconjugation of enzyme ligands and antibodies,^36^ whereas the C4′ position of the heptamethine chain can be used to tune absorption spectra^37^ or photostability,^38^ or to attach targeting residues.^39^, ^40^ The phenyl substituent of the flavonol moiety is introduced at a late stage of the synthesis, which allows for the installation of additional substituents. In this work, we present a strategy to access potent NIR‐absorbing photoCORMs by designing flavonol‐cyanine hybrids **5 a** and **b** (Figure 1 c), and we describe their photochemical behavior and demonstrate their biological applicability in in vivo experiments.

**Scheme 1:**
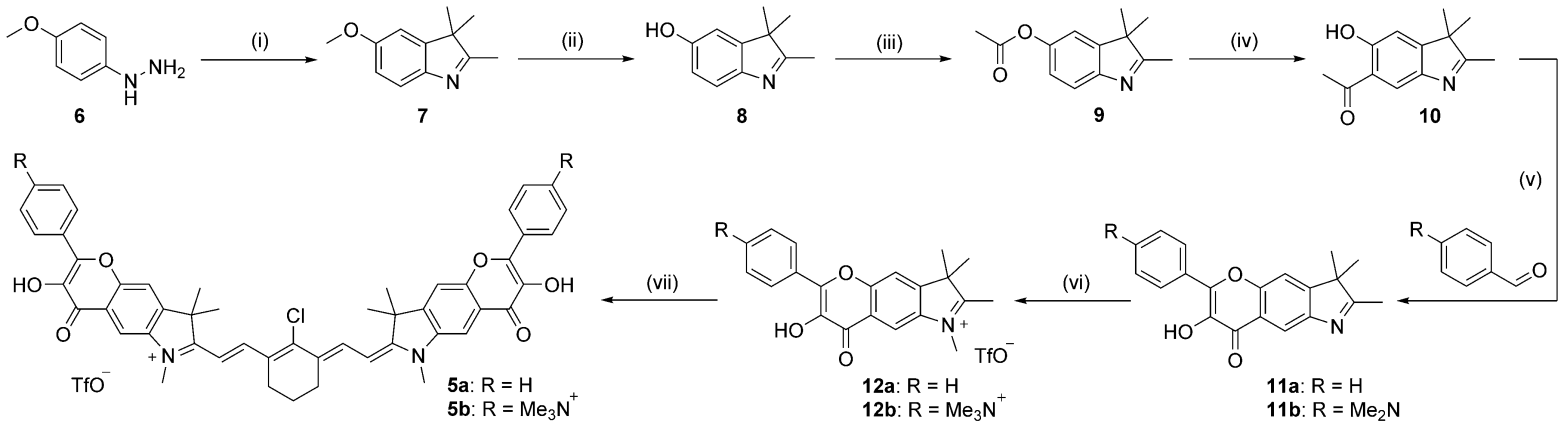
Synthesis of the cyanine‐flavonol hybrids **5 a**–**b**. Reaction conditions: (i) 3‐methyl‐2‐butanone, AcOH, 110 °C, 97 %; (ii) BBr_3_, CH_2_Cl_2_, 0 °C to rt, 95 %; (iii) AcCl, Et_3_N, CH_2_Cl_2_, rt, 59 %; (iv) AlCl_3_, neat, 190 °C, 80 %; (v) NaOH, 30 % H_2_O_2_, MeOH, 0 °C. **11 a**: 22 %. **11 b**: 38 %; (vi) **12 a**: MeOTf, CH_2_Cl_2_, 86 % or **12 b**: 1) MeI, MeCN, 100 °C; 2) AgOTf, MeOH 81 %; (vii) enamine **13**, AcONa, EtOH, reflux. **5 a**: 55 %. **5 b**: 51 %.

We synthesized hybrid **5 a** and its analog **5 b** featuring trimethylammonium substituents to facilitate its solubility in aqueous media (Figure 1 c). We reasoned that the flavonol core must be constructed prior to assembling the cyanine scaffold, which is susceptible to strongly basic and oxidative conditions required in the flavonol preparation. The synthesis of **5 a**–**b** was started by the construction of an indolenine core **7** from **6** and 3‐methyl‐2‐butanone in 97 % yield (Scheme 1). The methoxy group of **7** was removed with BBr_3_, and the resulting phenolic group was acylated with acetyl chloride. Subsequent Fries rearrangement of **9** with AlCl_3_ at 190 °C provided the desired methylketone **10** in 80 % yield in a regioselective fashion, presumably because of steric hindrance imposed by the dimethylmethylene substituent. Algar–Flynn–Oyamada reaction of **10** with benzaldehyde or 4‐(dimethylamino)benzaldehyde in the presence of H_2_O_2_ provided flavonols **11 a**–**b** in 22 % and 38 % yields, respectively. Methylation with TfOMe or MeI afforded the building blocks **12 a**–**b**, which were subsequently condensed with commercially available enamine **13** (see the Supporting Information) to access the final cyanine‐flavonol hybrids **5 a**–**b** in 55 % and 51 % yields, respectively.

**Figure 2:**
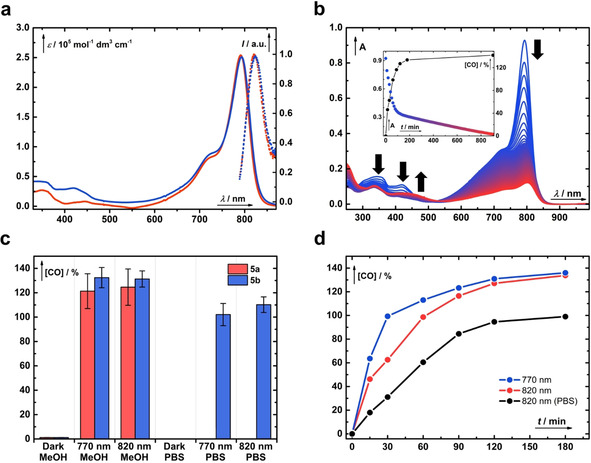
(a) UV/Vis absorption (solid) and emission (dotted) spectra of the hybrids **5 a** (red) and **5 b** (blue). (b) Irradiation of **5 b** (*c*≈3.8×10^−6^ m) at 820 nm in aerated methanol followed by UV/Vis spectroscopy at 10 min intervals (from blue to red lines). The inset depicts a kinetic trace of the absorption at 793 nm superimposed with the liberation of CO in time (black). (c) Total chemical yields of CO produced in the dark or upon exhaustive irradiation of **5 a** (red) or **5 b** (blue) in methanol or PBS (pH 7.4, 10 mm, *I*=100 mm) at 770 or 820 nm, respectively. The error bars represent the standard deviation of the mean from four independent samples. (d) Time‐dependent CO release from **5 b** (*c*≈4×10^−6^ m); blue line: irradiated at 770 nm in methanol; red line: irradiated at 820 nm in methanol; black line: irradiated at 820 nm in PBS. The CO released to the headspace was determined by GC and is expressed as the total chemical yield.

UV/Vis absorption spectra of cyanine‐flavonol hybrids **5 a**–**b** in methanol display an intense absorption band typical for the heptamethine cyanine dyes with *λ*_max_=791 and 793 nm, respectively (Figure 2 a and Table 1).^37^ The compounds also exhibit emission at *λ*_em_=815 and 819 nm, respectively, with the intensity comparable to that of indocyanine green (ICG). Compound **5 b** in methanol or DMSO at spectroscopic concentrations was found stable at 4 °C for days.

**Table 1:** Photophysical and photochemical properties of cyanine‐flavonol hybrids **5 a** and **5 b**.

	*λ*_abs_ [nm]	*λ*_em_ [nm]	*ϵ*_max_^[a]^ [m ^−1^ cm^−1^]	*Φ*_Δ_ [%]^[b]^	Yield [%]^[c]^	*Φ*_CO_ [%]^[d]^	*Φ*_CO_ *ϵ*_max_^[e]^ [m ^−1^ cm^−1^]
**5 a**	791	815	25 4000	0.34±0.01	125±15	(2.0±0.3)×10^−2^	51±8
**5 b**	793	819	25 1000	0.62±0.02	131±6	(3.0±0.5)×10^−2^	75±12

All measurements were performed in methanol. [a] The molar absorption coefficient, *ϵ*_max_. [b] The singlet oxygen production quantum yield was determined by using diphenylisobenzofurane (DPBF). Compounds **5 a** and **5 b** were irradiated with 770 nm LEDs and ICG as a ^1^O_2_ generator (*Φ*_Δ_=0.0077) and used as a reference. [c] The total chemical yield of released CO, monitored by GC headspace, obtained upon exhaustive irradiation at 770 nm. [d] Absolute quantum yields of CO release at *λ*_max_ determined by using a calibrated Si‐photodiode. [e] The CO uncaging cross‐section at *λ*_max_: *Φ*_CO_ *ϵ*_max_.

Irradiation of **5 a**–**b** (*c*≈4×10^−6^ m) in methanol at 770 (≈60 mW cm^−2^) or 820 nm (≈14 mW cm^−2^) with commercial LEDs was initially (≈50 min) accompanied by a small hypsochromic shift (≈3 nm) of the major absorption bands and a decrease of their intensities, and concurrent disappearance of the absorption bands at 343 and 422 nm (Figure 2 b and Figure S52 in the Supporting Information). Because the substitution of the aromatic cores of the cyanine heterocycles affects the cyanine absorption maxima relatively insignificantly owing to its weak interaction with the HOMO and LUMO of the cyanine chromophore, we hypothesized that this process corresponds to the photochemical cleavage of the terminal flavonol rings (see below). Upon extended irradiation, the disappearance of the major absorption bands at approximately 790 nm and the appearance of very weak bands at about 420 nm were observed, which is attributed to the subsequent photooxidation of the cyanine heptamethine chain with singlet oxygen produced by sensitization of the oxygen present in the solution (Figure S63 in the Supporting Information).^41^ Self‐sensitized photooxygenation is known to be a primary photobleaching pathway of cyanines and has recently been utilized for NIR uncaging.^42^, ^43^ These two distinct photochemical processes were also clearly distinguished for **5 b** from the kinetic trace detected at 793 nm (Figure 2 b, inset). The phototransformation efficiency of **5 b** in degassed methanol was severely suppressed; only an approximately 20 % decrease in absorbance at 793 nm was observed after irradiation for 16 h (Figures S54 and S55 in the Supporting Information).

In the next step, we assessed whether the photolysis of **5 a**–**b** with NIR light is accompanied by the anticipated formation of CO (Figure 2 c). Septum‐sealed vials containing **5 a**–**b** (*c*≈4–10×10^−6^ m) in methanol or phosphate‐buffered saline (PBS, pH 7.4, 10 mm, *I*=100 mm) were irradiated or kept in the dark, and the headspace above the solution was analyzed by using gas chromatography. Indeed, exhaustive irradiation of **5 a**–**b** in methanol at 820 nm resulted in the formation of CO in (125±15) % and (131±6) % chemical yields, respectively, whereas the samples kept in the dark liberated only negligible (<2 %) amounts of CO, demonstrating that the release is of photochemical origin. The photolysis of water‐soluble **5 b** in PBS (pH 7.4, 10 mm, *I*=100 mm) at 770 or 820 nm generated CO in (102±9) % and (110±6) % chemical yields, respectively, whereas no CO was detected in the samples kept in the dark. Irradiation of the parent cyanine **4** for 16 h did not lead to the production of CO (<2 %). These results show that **5 a–b** can release in principle two molecules of CO from both flavonol moieties, corresponding to approximately 65 % and 55 % yields of CO per flavonol unit in methanol and PBS, respectively. The chemical yield values obtained in both methanol and aqueous solutions are slightly lower than those observed for the parent flavonol **1**.^29^ The majority of CO was released within 1–2 h of irradiation (Figure 2 d) and is related to the first photochemical process detected by UV/Vis spectroscopy (Figure S57 in the Supporting Information). Under the given conditions, the therapeutic levels of CO (1 μm, 44 corresponding to ≈25 % chemical yield) were reached after about 15 min of irradiation and the total dose of light (≈6.3 W cm^−2^) is comparable to doses routinely used in PDT.^45^ On the contrary, solutions of **5 b** in degassed methanol prepared under hypoxic conditions (≈3 ppm of O_2_ in the atmosphere) and irradiated at 770 nm produced CO less efficiently (≈30 %, ≈108 %, and ≈128 % after 1, 24, and 36 h, respectively). This demonstrates the pivotal role of oxygen in the photodecarbonylation process but also indicates that the system can operate under the hypoxic conditions often found in tumors, albeit with lower efficiency (Figure S62 in the Supporting Information). Performing the photolyzes in minimum essential medium (MEM) or the presence of bovine serum albumin (BSA, 20 mg mL^−1^) did not influence the chemical yield of the released CO. Irradiation of **5 b** in aerated methanol at 420 nm produced CO in approximately 160 % chemical yield.

Subsequently, the efficiency of the NIR‐light‐triggered CO photorelease was assessed. Owing to the lack of suitable actinometers operating in the NIR region, we instead opted to use a calibrated Si‐photodiode to measure the photon flux. Coupling this with GC headspace analysis allowed us to directly determine the absolute quantum yields of CO release (*Φ*_CO_) to be (2.0±0.3)×10^−4^ and (3.0±0.5)×10^−4^ for **5 a** and **5 b** in aerated methanol, respectively (Table 1). The excellent uncaging cross‐section *Φ*_CO_ *ϵ*_max_ of **5 a**–**b** on the order of 50–75 m ^−1^ cm^−1^ is comparable to that of the parent flavonol **1** (≈84 m ^−1^ cm^−1^)^26^ when operating at wavelengths bathochromically shifted by nearly 400 nm. The only transition‐metal‐free photoCORM **2** activated by near‐infrared light reported to date possesses *Φϵ*_max_ of 0.6.^30^ Compared with this value, the present system constitutes up to 110‐fold increase in the efficiency of the photochemical CO release with a simultaneous shift of the absorption maximum by approximately 140 nm to the NIR region. The strong absorption in the phototherapeutic window, high chemical yields of CO, and excellent uncaging cross‐section *Φ*_CO_ *ϵ*_max_ showcase the practical utility and applicability of the hybrids **5 a**–**b** in biological settings.

**Figure 3:**
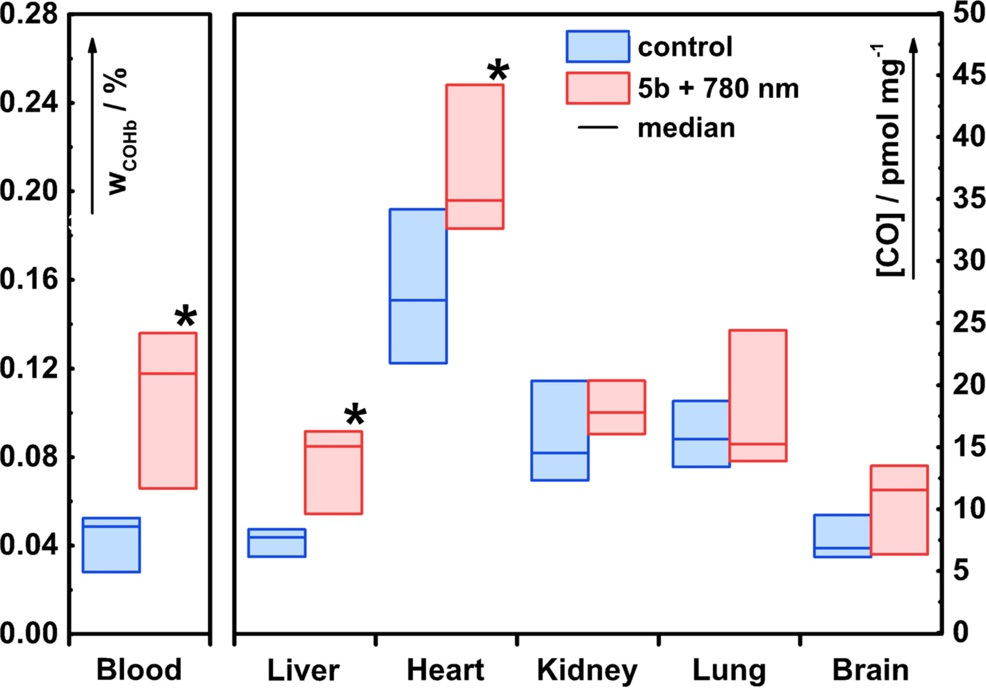
CO release from **5 b** upon irradiation in vivo. A control group of eight animals (only vehicle application) is depicted in blue. Another group of six animals (red) received an intraperitoneal injection of **5 b** (50 μmol kg^−1^ of body weight) in vehicle (saline 10 μL g^−1^, 5 % DMSO) and was irradiated with 780 nm light focused on the abdominal area for 2 h. The amounts of CO are expressed as the relative amounts of COHb in the total amount of Hb in the blood (*w*_COHb_ in %; left ordinate) or in pmol mg^−1^ of fresh organ tissue (right ordinate). The horizontal lines represent the median, the boxes show the interquartile range. A statistically significant increase of CO was observed in the blood, liver, and heart tissues of the irradiated mice (* *P* value ≤0.05 vs. “control group”; *n=*6).

The water‐soluble derivative **5 b** exhibited cytotoxic effects in in vitro studies on the HepG2 hepatoblastoma cell line after a 24 h exposure from concentrations as high as 100 μm (Figure S72 in the Supporting Information). We further probed the cytotoxic effects of the photoproducts by exhaustive irradiation of **5 b** in the absence of HepG2 cells and subsequent exposure of HepG2 cells to the mixture of photoproducts for 24 h (Figure S73 in the Supporting Information). This approach enabled us to assess the effects of photoproducts independently from those of CO or ^1^O_2_. The photoproducts were found to be non‐toxic up to concentrations as high as 200 μm. Irradiation of **5 b** at 780 nm for 1 h incubated together with the presence of HepG2 cells (Figure S74 in the Supporting Information) had no significant effect on cell viability up to the concentration of 100 μm as measured 24 h post‐irradiation, indicating the low toxicity of the photoproducts. Encouraged by these findings, we decided to evaluate the performance of **5 b** in in vivo experiments on nude SKH1 mice. Hairless mice were used to facilitate light penetration through the cutaneous barrier. One group of mice was not treated with **5 b**, but received vehicle only, and served as a control group. Another group received an intraperitoneal injection of **5 b** (50 μmol kg^−1^ of body weight) in saline (10 μL g^−1^, with 5 % DMSO), and the mice were irradiated for 2 h with NIR light (*λ*_irr_=780 nm) focused on the abdominal area. Irradiation resulted in a substantial increase of carbonylhemoglobin (COHb) in the blood and of the CO content in the liver and heart tissues when compared with the control group (Figure 3). A significant increase in the CO levels (up to 3‐fold) in both the blood and tissues of the irradiated mice demonstrates that the photorelease of CO from **5 b** also occurs in vivo.

We sought to provide additional support for the circumstantial evidence obtained from Figure 2 b that the initial photodecarbonylation process competes with a significantly less efficient photooxidation of the Cy7 scaffold. Thus, the production of singlet oxygen by sensitization of **5 a**–**b** and the reactivity of the individual molecular fragments towards oxygenation were examined. The quantum yields of singlet oxygen formation (*Φ*_Δ_) in methanol were found to be (3.4±0.1)×10^−3^ and (6.2±0.2)×10^−3^, respectively, values which are comparable to those of analogous cyanines and lower than those of FDA‐approved ICG.^46^ The extended flavonol **1**, which was previously utilized in several biological studies, possesses *Φ*_Δ_ of 14 %. The ratio *Φ*_Δ_/*Φ*_CO_, which can serve as a handle to assess how much singlet oxygen molecule is generated per molecule of released CO, is more favorable for **5 b** than for **1** by a factor of 2. We assumed that the generated singlet oxygen can either cleave the flavonol units with the concurrent expulsion of CO^29^ or undergo addition to the polyene chain of cyanine to destroy the chromophore.^41^ In our previous study, a neutral form of the parent flavonol **1** was shown to be relatively unreactive towards singlet oxygen in methanol with a bimolecular rate constant *k*_r_ of 4.3×10^5^ m ^−1^ s^−1^.^29^ The *k*_r_ values for **1** as well as for **14 B** (see below) are in good agreement with those reported for analogous **3**.^47^ On the contrary, the parent Cy7 scaffold **4** was found to be more reactive by nearly two orders of magnitude with *k*_r_ of 1.7×10^7^ m ^−1^ s^−1^, the same order of magnitude as those for heptamethine cyanines.^48^ Applying a steady‐state approximation for singlet oxygen as a reactive intermediate and employing the quantum yields *Φ*_CO_ and *Φ*_Δ_ determined herein, we found that the CO release quantum yield is about two orders of magnitude higher than that of self‐sensitized polyene photooxygenation (see the Supporting Information). This implies that singlet oxygen quenching by the solvent is a dominant deactivation pathway at low concentrations of cyanine.

**Scheme 2:**
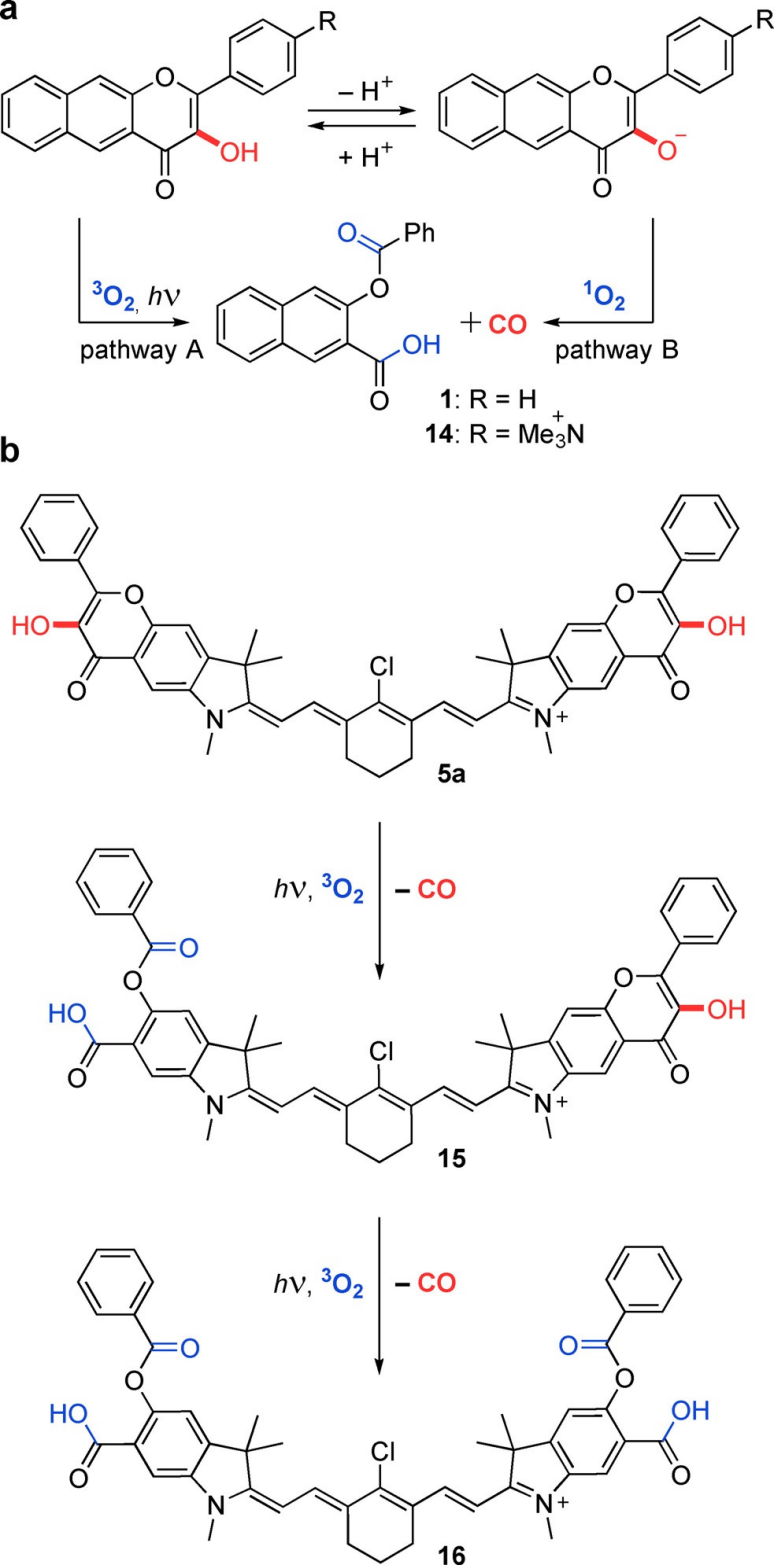
The proposed mechanism of photochemical CO release from **5 a**–**b**. (a) The major orthogonal photodecarbonylation pathways of model flavonols **1** and **14** under aerobic conditions. (b) Sequential photodecarbonylation from **5 a**–**b** at both terminal flavonol units.

In our recent mechanistic study of parent flavonol **1**, two orthogonal pathways of CO release were identified under aerobic conditions (Scheme 2).^29^ Both the triplet excited state of acid **1 A** and ground state of base **1 B** can undergo decarbonylation. The former species reacts with ground‐state oxygen (^3^O_2_, pathway A), whereas the latter reacts with singlet oxygen (^1^O_2_, pathway B). Both **5 a** and **5 b** exist as conjugate acids in DMSO, evidenced by the signal of phenolic groups in their ^1^H NMR spectra. In analogy to **1** (p*K*_a_≈9.3), we also assume that **5 a**–**b** in methanol exist exclusively as the conjugate acid. HRMS analysis of the sample of **5 a** in methanol (*c*≈4×10^−5^ m) irradiated at 820 nm for 2 h revealed the presence of the anticipated photoproducts (**15** and **16**; Figures S63–S64 in the Supporting Information), analogous to **1** (Scheme 2 a), arising from the expulsion of CO from either single or both terminal flavonol units, respectively (Scheme 2 b). As expected, the ratio of the **15** and **16** concentrations varied with the time of irradiation, and we found that the cleavable benzoate ester moiety in these photoproducts is spontaneously liberated (**17** and **18**; see the Supporting Information). The same thermal reactivity of the ester was observed for the photoproducts of parent flavonol **1**.^29^ A complex mixture of products is obtained upon exhaustive irradiation owing to the photodegradation of the cyanine scaffold (Figure S63 in the Supporting Information). However, we were able to detect some of the species formed by photooxidative cleavage of the cyanine scaffold at the C2‐C1′ and C2‐C3′ positions (Figure S65 in the Supporting Information), which is in excellent agreement with the extensive work of Schnermann and co‐workers.^41^

A detailed mechanistic study of **5 b** in aqueous media was complicated by its propensity to form aggregates. Hence, we decided to use simplified flavonol **14** as a model compound for this purpose. The p*K*_a_ of the OH group in **14** in water was determined to be p*K*_a_=7.98, which is lower than that of the parent flavonol **1** (p*K*_a_≈9.3)^29^ owing to the presence of an electron‐withdrawing group. Therefore, flavonol **14** exists as a mixture of conjugate acid **14 A** and base **14 B** (≈20 %) at pH 7.4 in PBS, and therefore, both decarbonylation pathways (Scheme 2 a) should be relevant. CO was released from **14** in PBS (pH 7.4, 10 mm, *I*=100 mm) upon irradiation at 400 nm in 81 % chemical yield and with a quantum yield of *Φ*_dec_=6.0×10^−3^. This value is consistent with that obtained for **1** in a DMSO/PBS mixture (pH 7.4, 1:1) by Berreau and co‐workers.^25^ Next, we estimated the efficiency of the photooxygenation of flavonol anion **14 B** by ^1^O_2_ from its *k*_r_ (*k*_r_=5.8×10^8^ m ^−1^ s^−1^), and the *Φ*_Δ_ value obtained for **5 b** under conditions identical to those employed in the irradiation experiments with **5 b** in PBS. The estimated value (*Φ*_calc_=3×10^−5^) was found to be lower by one order of magnitude than that of the Cy7 photobleaching in PBS (*Φ*_dec_≈1.8×10^−4^), suggesting that the pathway involving ^1^O_2_ (pathway B) is ineffective for **5 b**, as it cannot compete with the destruction of the Cy7 chromophore. Furthermore, the production of CO from **5 b** in methanol or PBS by irradiation at 820 nm was not suppressed in the presence of a large excess of diphenylisobenzofurane (DPBF, 1500 equiv) or furfuryl alcohol (5000 equiv), respectively, as singlet oxygen traps (Figures S59–S60 in the Supporting Information). Based on these considerations and in analogy to the behavior of **1**, we propose that the singlet oxygen decarbonylation pathway is inefficient for **5 a**–**b**, and the photorelease of CO occurs predominantly through the reaction of their triplet excited states with ground‐state oxygen (pathway A) in concentrations that are common in biological aqueous media under aerobic conditions.

## Conclusion

We have developed a new class of transition‐metal‐free photoCORMs by fusing an established CO‐releasing flavonol moiety with a NIR‐absorbing cyanine chromophore. The resulting hybrids liberate CO in high chemical yields upon activation with NIR light up to 820 nm and with excellent uncaging cross sections. Their photochemical performance, in vitro biocompatibility, minimal intrinsic phototoxicity as well as applicability in in vivo experimental settings, promise many avenues for their future applications. In a more general sense, we have shown that this concept can serve as an inspiration for the discovery of new types of photoCORMs, and we believe that it represents a crucial step towards light‐activated targeted delivery of CO for therapeutic purposes.

## Experimental Section

Animal studies: Male nude SKH1 mice were used. All studies in this work met the criteria for the care and use of animals and were approved by the Animal Research Committee of the 1st Faculty of Medicine, Charles University, Prague.

## Conflict of interest

The authors declare no conflict of interest.

## Supporting information

As a service to our authors and readers, this journal provides supporting information supplied by the authors. Such materials are peer reviewed and may be re‐organized for online delivery, but are not copy‐edited or typeset. Technical support issues arising from supporting information (other than missing files) should be addressed to the authors.

Supplementary
