# Supplementary material for: Cyanine‐Flavonol Hybrids for Near‐Infrared Light‐Activated Delivery of Carbon Monoxide
Source: Chemistry. 2020 Sep 4;26(58):13184–90. doi: 10.1002/chem.202003272 (PMC7693251; doi:10.1002/chem.202003272)
Supplement: Supplementary file 1 — Supplementary [file CHEM-26-13184-s001.pdf]

Chemistry–A European Journal

Supporting Information

Cyanine-Flavonol Hybrids for Near-Infrared Light-Activated Delivery of Carbon Monoxide

Lenka Štacková,[a] Marina Russo,[a] Lucie Muchová,[b] Vojtěch Orel,[a] Libor Vítek,[b] Peter Štacko,*[a] and Petr Klán*[a]

Supporting Information

Table of Contents

Materials and Methods	S2
Synthesis	S6
References	S11
NMR Spectra and Assignments	S12
HRMS Analysis	S51
UV-Vis Photochemical Experiments	S56
GC-Headspace Analysis	S59
Photoproducts of **5b**	S64
Emission of LEDs	S67
Spectroscopic Determination of pKa of **14**	S68
Irradiation of **14**	S68
Competition between CO Release and Cyanine Photooxygenation	S70
Cytotoxicity of **5b** and its Photoproducts	S71

Materials and Methods

Reagents and solvents of the highest purity available were used as purchased, or they were purified/dried using standard methods when necessary. Synthetic procedures were performed under an ambient atmosphere unless stated otherwise.

Flash column chromatography was performed using silica gel (230–400 mesh). ^{1}H NMR spectra were recorded on 300 or 500 MHz spectrometers; ^{13}C NMR were obtained on 125 MHz or 75 MHz instruments in $CDCl_3$, CD_3OD, and d_6-DMSO. ^{1}H chemical shifts are reported in ppm relative to tetramethylsilane (δ = 0.00 ppm) using the residual solvent signal as an internal reference. ^{13}C chemical shifts are reported in ppm with $CDCl_3$ (δ = 77.67 ppm) and CD_3OD (δ = 49.30 ppm) as internal references. The deuterated solvents were kept under nitrogen atmosphere.

UV-vis spectra were obtained with matched 1.0 cm quartz cuvettes. Fluorescence was measured on an automated luminescence spectrometer in 1.0 cm quartz fluorescence cuvettes at 26 ± 1 °C. The corresponding optical filters were used to avoid the second harmonic excitation/emission bands induced by the grating. Sample concentrations with the absorbance below 0.1 at the excitation wavelength at the absorption maxima were used. Each sample was measured five times, and the spectra were averaged. Emission and excitation spectra were normalized and smoothed using standard protocols.

The exact masses of the synthesized compounds were obtained using a triple quadrupole electrospray ionization mass spectrometer in a positive or negative mode coupled with direct-inlet or liquid chromatographies.

General Procedure for Irradiation in UV Cuvettes. A solution of a compound in the given solvent (3 mL) in a matched 1.0 cm quartz PTFE screw-cap cuvette equipped with a stirring bar was stirred and irradiated with a light source of 32 LEDs (λ_{max} = 770 or 820 nm at ~60 or 14 mW/cm^{-2}, respectively; Supplementary Fig. 67). The progress of the reactions was monitored at the given time intervals by UV–vis spectrometry using a diode-array spectrophotometer.

Determination of CO Yields. Stock solutions of **5a** in CH_2Cl_2 or **5b** in methanol or DMSO (c ~ 1×10^{-4} M) were diluted with methanol or PBS (pH 7.4, 10 mM, I = 100 mM) to give a final concentration of c ~3–50 × 10^{-6} M, so that the amount of a co-solvent does not exceed 2.5% (v/v). The solutions (100–1000 μL) in closed GC vials fitted with PTFE septa were irradiated with LEDs at 770 or 820 nm (~30 and ~7 mW cm^{-2}, respectively) for the given times or to complete conversion. The released CO was analyzed and quantified by a GC–headspace instrument (5Å molecular sieve packed column) equipped with a TIC/MS detector in a SIM mode, which was calibrated using the quantitative photoreaction of cyclopropenone photoCORM (50–600 μL, c ~ 1 × 10^{-5} M, in methanol).[1]

Determination of CO Release Quantum Yields. Solutions of **5a** or **5b** in methanol (1000 μL, c ~ 1 × 10^{-6} M) in closed GC vials fitted with PTFE septa were irradiated by a xenon short-arc lamp through a monochromtator set to 791 and 793 nm, respectively. The samples were

irradiated through the bottom of the vial to minimise reflection of light. The absolute photon flux was measured by a calibrated Si-photodiode. The total molar amount of the released CO was analyzed by GC–headspace as described above and used to calculate the quantum yields of CO release.

Fluorescence Measurements. Fluorescence and excitation spectra were measured using a fluorescence spectrometer in a 1.0 cm quartz fluorescence cuvette at 23 ± 1 °C. The sample concentrations were adjusted to keep the absorbance below 0.2 at the corresponding excitation wavelength. Each sample was measured five times, and the spectra were averaged. Emission and excitation spectra were normalized and corrected by the photomultiplier sensitivity function using correction files supplied by the manufacturer.

Singlet Oxygen Production Quantum Yields. Solutions of 1,3-diphenylisobenzofuran (DPBF; $c = 4 \times 10^{-5}$) and either one of **5a**–**b** ($c \sim 1 \times 10^{-6}$ M) or indocyanine green (ICG, $c = 6 \times 10^{-6}$ M) as photosensitizers in methanol were prepared. The stirred solution (3.5 mL) in a quartz cell (1 cm) was irradiated using LEDs at 770 nm, and the UV−vis spectra were recorded periodically. The overall irradiation time was selected to reach <10% conversion of DPBF. The procedure was repeated 3 times. The decomposition of DPBF monitored at 411 nm was fitted with a pseudo-first-order rate law. The data were corrected for light absorbed by the samples, calculated as an integral of the overlap between the absorption and LED emission spectra. The singlet oxygen formation quantum yield Φ_Δ was calculated using ICG as a reference (Φ_Δ = 0.008[2,3])

Quantum Yields of Decomposition. A solution of a commercially available IR-783 dye ($c = 6.5 \times 10^{-6}$ M, 3.0 mL) in PBS (pH 7.4, 10 mM, I = 100 mM) was stirred and left to equilibrate for 2–3 min at 20 °C. Afterwards, irradiation using LEDs at 730 or 770 nm was initiated, and UV−vis spectra were recorded periodically. The total irradiation time was chosen to reach a <10% conversion and to obtain 10 experimental points. The procedure was repeated 3 times. The quantum yield of decomposition Φ_{dec} was calculated using the equation Eq. S1:

$$\phi_{\mathrm{dec}} = \phi_{\mathrm{dec}}^{\mathrm{REF}} \frac{\Delta n}{I} \frac{I_{\mathrm{REF}}}{\Delta n_{\mathrm{REF}}} \qquad (\mathrm{Eq.\,S1})$$

where Φ^{REF} is the decomposition quantum yield of the reference compound, Δn is the number of moles of the photodecomposed IR-783 dye calculated from the absorbance change at λ_{max}, and I is the total amount of the light absorbed by a sample in the given time period, calculated according to the equation Eq. S2:

$$I = \int_0^t \int_0^\infty \left(1 - 10^{-A(\lambda,t)}\right) I_\lambda^{em}\, d\lambda\, dt \qquad (\mathrm{Eq.\,S2})$$

where $A(\lambda,t)$ is absorbance of the sample at the wavelength λ in time t, and I_λ^{em} is the emission intensity of a LED source at the wavelength λ. Due to the lack of available chemical actinometers, photooxygenation of DPBF by ICG (Φ_Δ = 0.008[2,3]) was used to measure the photon flux.

Reaction Rate with $^1O_2$. A solution containing **14** ($c = 5 \times 10^{-5}$ M) or **4** ($c = 4 \times 10^{-6}$ M) and rose bengal (RB, $c = 1 \times 10^{-5}$ M) as a singlet oxygen sensitizer in glycine buffer (pH 9.8, 20 mM) solution (3.0 mL) in a quartz cell (1.0 cm) was stirred and irradiated with LEDs at 545 nm. The UV-vis absorption spectra were recorded periodically. The total irradiation time was selected to reach a <10% conversion of **14B** (or **4**) and to obtain 10 experimental points. The procedure was repeated 3 times. The bimolecular reaction rate constant for **14B** (or **4**) with $^1O_2$ (k_r) was calculated from the equation Eq. S3:

$$\beta = \beta_{\mathrm{REF}} \frac{\Delta n}{cI} \frac{I_{\mathrm{REF}} c_{\mathrm{REF}}}{\Delta n_{\mathrm{REF}}}; \quad \beta = \frac{k_r c}{k_d + k_r c} \quad \text{(Eq. S3)}$$

where Δn is the number of moles of the photodecomposed **14B** or **4,** calculated from the absorbance change at $\lambda_{\max}$, c is the initial concentration of **14B** or **4**, k_d is the known rate constant of singlet oxygen quenching in methanol ($k_d = 9.7 \times 10^4$ s^{-1})[4] or PBS ($k_d = 2.5 \times 10^5$ s^{-1}), and I is the total amount of the light absorbed by the sample in the given time period calculated according to Eq. S2. The bimolecular rate constant k_r for **14B** and **4** was found to be 5.8×10^8 and 1.7×10^7 M^{-1} s^{-1}, respectively, determined relative to that of **1B** ($k_r = 4.7 \times 10^8$ M^{-1} s^{-1}).[5]

In Vitro Toxicity Determination. Human hepatoblastoma HepG2 cells were grown according to the standard protocol in a 96-well plate. After reaching 80% confluence, cells were treated with a solution of **5b** or its photoproducts in the concentration range of 6−200 μM (with 2% DMSO) for 2, 6, or 24 h. The cell viability was determined by a 3-(4,5-dimethylthiazol-2-yl)-2,5-diphenyltetrazolium bromide assay (MTT). The absorbance of a formazan-reduction product was measured at 545 nm with a standard ELISA reader. Compound **5b** was found not to interfere with the reduction of MTT.

In Vivo Experiments. Male nude SKH1 mice were allowed water and standard granulated diet ad libitum. Mice were anesthetised and then received an intraperitoneal injection of saline with 5% DMSO (a control group) or a solution of **5b** (50 μmol kg^{-1} of body weight) in saline (10 μL g^{-1}, with 5% DMSO). An experimental group of mice was irradiated with 780 nm LED (4 × 500 mW) focused to the abdominal area for 2 h. Subsequently, the animals were sacrificed. Blood from the superior vena cava of each animal was transferred to sodium heparin-coated test tubes containing aq. EDTA (2 μL, 0.5 M). The CO (as COHb) and total hemoglobin concentrations in the sample of blood (1 μL) were determined using GC and a Drabkin cyanmethemoglobin method described previously.[6] Selected organs of each animal were then harvested, washed, put into an ice-cold potassium phosphate buffer (pH 7.4, 100 mM) in a ratio of 1:4 (w/w) and homogenised by sonication. The homogenate (40 μL) was added to CO-free septum-sealed vials containing sulfosalicylic acid (5 μL, 60% v/w). After incubation on ice (30 min), the amount of the CO released into the vial headspace was determined by GC/RGA. All studies in this work met the criteria for the care and use of animals and were approved by the Animal Research Committee of the 1st Faculty of Medicine, Charles University, Prague.

Statistical Analysis. Normally distributed data are presented as the mean ± SD and analyzed by the Student t-test. The Mann–Whitney *U* test was used in skewed data expressed as a median ± interquartile range. Differences in $P < 0.05$ were considered significant.

Synthesis

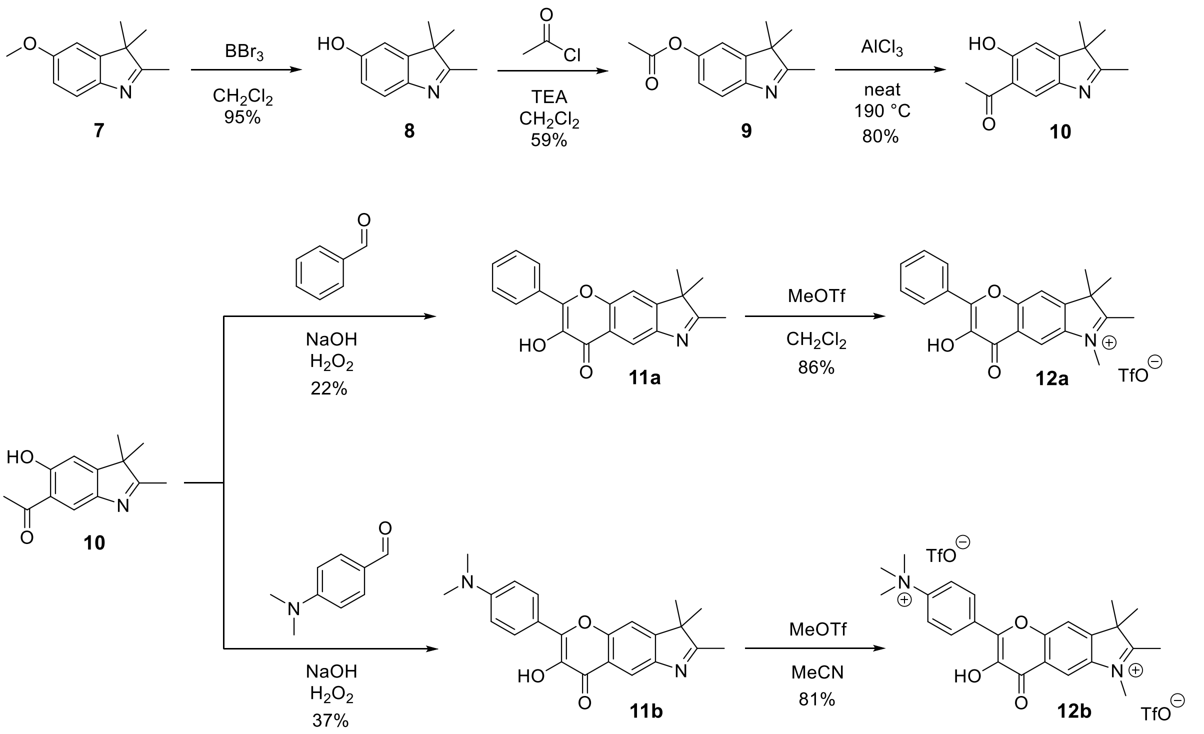

Scheme 1. Synthesis of the flavonol-based *N*-heterocycles **12a** and **12b**.

5-Methoxy-2,3,3-trimethyl-3*H*-indole (7).

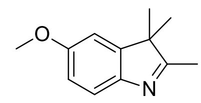

Following the published procedure,[7] 4-methoxyphenylhydrazine hydrochloride (15.0 g, 85.9 mmol) was added to a solution of methylbutan-2-one (14.8 g, 172 mmol) in glacial acetic acid (200 mL), and the reaction mixture was refluxed for 2 h. The solvent was evaporated at reduced pressure, and the crude product was purified by column chromatography (silica gel, hexane/ethyl acetate, 2 : 1). Yield: 15.7 g (97%). Brown viscous liquid. ^{1}H NMR (300 MHz, $CDCl_3$): δ (ppm) 7.42 (dd, J_1 = 8.0 Hz, J_2 = 0.5 Hz, 1H), 6.84−6.77 (m, 2H), 3.80 (s, 3H), 2.23 (s, 3H), 1.27 (s, 3H). ^{13}C NMR (75 MHz, $CDCl_3$): δ (ppm) 186.0, 158.1, 147.4, 147.3, 120.2, 112.2, 108.3, 55.8, 53.9, 23.3, 15.3. NMR spectra are consistent with published data.[8] HRMS (ESI+): calcd. for $C_{12}H_{16}NO^+$ [M + H^+] 190.1226, found 190.1228.

2,3,3-Trimethyl-3*H*-indol-5-ol (8).

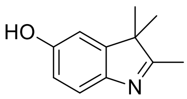

BBr_3 in CH_2Cl_2 (1 M, 99.1 mL, 99.1 mmol) was added dropwise to a solution of indolenine **7** (12.5 g, 66.1 mmol) in dry CH_2Cl_2 (130 mL) at 0 °C. The reaction mixture was stirred for 2 h at 0 °C, then it was allowed to warm to rt and stirred for additional 16 h. The mixture was then diluted with water (500 mL) and neutralized with sat. aq. $NaHCO_3$ (pH = 7). The solution was extracted with CH_2Cl_2 (3 × 500 mL), the combined organic extracts were dried with Na_2SO_4, and the solvents evaporated at reduced pressure. The product was obtained pure without any additional purification. Yield: 11.0 g (95%). Brown solid. Mp. 156.7−157.9 °C. ^{1}H NMR (300 MHz, d_6–DMSO): δ (ppm) 9.43 (brs, 1H), 7.23 (d, J = 8.2 Hz, 1H), 6.84 (d, J = 2.4 Hz, 1H), 6.70 (dd, J_1 = 2.4 Hz, J_2 = 8.3 Hz, 1H), 2.21 (s, 3H), 1.22 (s, 6H). ^{13}C NMR (75 MHz, d_6–DMSO): δ (ppm) 185.6, 155.9, 147.0, 143.4, 119.0, 113.8, 109.4, 53.1, 22.6, 14.7. HRMS (ESI+): calcd. for $C_{11}H_{14}NO^+$ [M + H^+] 176.1070, found 176.1071.

2,3,3-Trimethyl-3*H*-indol-5-yl Acetate (9).

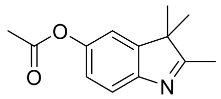

Acetyl chloride (5.8 g, 74.30 mmol) was added dropwise to a solution of **8** (10.9 g, 61.9 mmol) and triethylamine (8.2 g, 80.5 mmol) in CH_2Cl_2 (250 mL) under N_2 atmosphere. The reaction mixture was stirred at rt for 16 h, quenched with water (250 mL), and the mixture was extracted with CH_2Cl_2 (2 × 150 mL). Combined organic extracts were washed with sat. aq. $NaHCO_3$ (100 mL), dried with $MgSO_4$, and the solvents were evaporated at reduced pressure. The crude product was purified by column chromatography (silica gel, hexane/ethyl acetate, 2 : 1, then switched to 1 : 1). Yield: 7.9 g (59%). Orange-brown solid. Mp. 63.7−65.2 °C. ^{1}H NMR (300 MHz, $CDCl_3$): δ (ppm) 7.49 (d, J = 7.9 Hz, 1H), 7.05–6.95 (m, 2H), 2.29 (s, 3H), 2.26 (s, 2H), 1.29 (s, 6H). ^{13}C NMR (75 MHz, $CDCl_3$): δ (ppm) 188.3, 169.7, 151.4, 148.6, 147.0, 120.7, 120.3, 115.2, 54.2, 23.1, 21.2, 15.5. HRMS (ESI+): calcd. for $C_{13}H_{16}NO_2^+$ [M + H$^+$] 218.1176, found 218.1179.

1-(5-Hydroxy-2,3,3-trimethyl-3*H*-indol-6-yl)ethan-1-one (10).

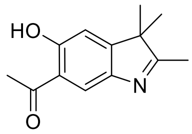

Indolenine **9** (6.78 g, 31.21 mmol) and $AlCl_3$ (20.8 g, 156.0 mmol) were mixed and heated without any solvent at 190 °C for 2 h. The mixture was then left to cool down to rt; ice (400 mL) and ethyl acetate (50 mL) were added, and the flask was sonicated until all the solid residue dissolved. The mixture was extracted with ethyl acetate (2 × 150 mL), the combined organic extracts were washed with $MgSO_4$, and the solvents were evaporated at reduced pressure. The crude product was purified by column chromatography (silica gel, hexane/ethyl acetate, 1 : 2). Yield: 5.4 g (80%). Brown solid. Mp. 107.3−109.2 °C. ^{1}H NMR (300 MHz, $CDCl_3$): δ (ppm) 12.55 (s, 1H, OH), 7.87 (s, 1H), 6.87 (s, 1H), 2.63 (s, 3H), 2.25 (s, 3H), 1.28 (d, 6H). ^{13}C NMR (75 MHz, $CDCl_3$): δ (ppm) 204.5, 186.8, 162.0, 155.1, 145.2, 120.5, 118.4, 112.0, 54.3, 26.9, 23.1, 15.2. HRMS (ESI+): calcd. for $C_{13}H_{16}NO_2^+$ [M + H$^+$] 218.1176, found 218.1178.

7-Hydroxy-2,3,3-trimethyl-6-phenylpyrano[2,3-*f*]indol-8(3*H*)-one (11a).

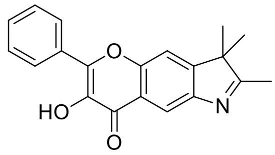

To a stirred solution of indolenine **10** (1.20 g, 5.52 mmol) and benzaldehyde (2.93 g, 27.6 mmol) in ethanol (20 mL) was added NaOH (1.10 g, 27.6 mmol) in water (2 mL). The resulting mixture was heated to 50 °C and stirred for 30 min. The solvents were evaporated at reduced pressure and the residue was redissolved in ethanol (20 mL). This process was repeated twice in order to shift the equilibrium towards the aldol product. Afterwards, hydrogen peroxide (0.19 g, 5.52 mmol) was added dropwise at 0 °C. The reaction mixture was stirred for 16 h, followed by neutralization with aq. HCl (pH = 5) and extraction with CH_2Cl_2 (3 × 40 mL). The combined organic extracts were washed with $MgSO_4$ and the solvents were evaporated at reduced pressure. The crude product was purified by column chromatography (silica gel, hexane : ethyl acetate – 1 : 1 then switched to 1 : 2). The product was then washed with minimal amount of methanol (0.5 mL) to give pure **11a**. Yield: 395 mg (22%). Slightly orange solid. Mp. 229.4−231.2 °C. ^{1}H NMR (300 MHz, $CDCl_3$): δ (ppm) 8.31 (s, 1H), 8.30−8.25 (m, 2H), 7.59−7.46 (m, 4H), 7.05 (brs, 1H), 2.35 (s, 3H), 1.41 (s, 6H). ^{13}C NMR (75 MHz, $CDCl_3$): δ (ppm) 189.1, 173.8, 154.5, 153.0, 151.1, 144.7, 138.4, 131.4, 130.2, 128.8, 127.8, 120.7, 115.0, 111.9, 54.3, 23.4, 15.7. HRMS (ESI+): calcd. for $C_{20}H_{18}NO_3^+$ [M + H$^+$] 320.1281, found 320.1283.

6-(4-(Dimethylamino)phenyl)-7-hydroxy-2,3,3-trimethylpyrano[2,3-*f*]indol-8(3*H*)-one (11b).

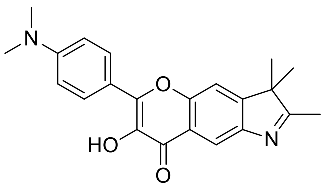

NaOH (0.92 g, 23.0 mmol) in water (2 mL) was added to a stirred solution of indolenine **10** (1.00 g, 4.60 mmol) and 4-(dimethylamino)benzaldehyde (2.06 g, 13.8 mmol) in methanol (40 mL). The resulting mixture was heated to 50 °C and stirred for 30 min. The solvents were evaporated at reduced pressure, and the residue was redissolved in methanol (40 mL). This process was repeated twice to shift the equilibrium toward the aldol product. Afterward, the mixture was cooled to 0 °C, and hydrogen peroxide (30%, 1.00 mL, 9.20 mmol) was added dropwise. The reaction mixture was stirred for 16 h, followed by neutralization with aq. HCl (pH = 7) and extraction with CH_2Cl_2 (3 × 40 mL). The combined organic extracts were washed with $MgSO_4$, and the solvents were evaporated at reduced pressure. The crude product was purified by column chromatography (silica gel, hexane/ethyl acetate, 1 : 1, then switched to 1 : 2) to give **11b**. Yield: 0.62 g (37%). Orange solid. Mp. 234.7−235.8 °C. ^{1}H NMR (300 MHz, $CDCl_3$): δ (ppm) 8.29 (s, 1H), 8.20 (d, J = 9.2 Hz, 2H), 7.46 (s, 1H), 6.95 (brs, 1H), 6.82 (d, J = 9.2 Hz), 3.08 (s, 6H), 2.34 (s, 3H), 1.40 (s, 6H). ^{13}C NMR (75 MHz, $CDCl_3$): δ (ppm) 188.7, 172.9, 154.2, 152.1, 151.5, 150.8, 146.4, 136.9, 129.3, 120.9, 118.6, 114.9, 111.8, 111.7, 54.2, 40.3, 23.5, 15.7. HRMS (ESI+): calcd. for $C_{22}H_{23}N_2O_3^+$ [M + H$^+$] 363.1703, found 363.1706.

7-Hydroxy-1,2,3,3-tetramethyl-8-oxo-6-phenyl-3,8-dihydropyrano[2,3-*f*]indol-1-ium Trifluoromethanesulfonate (12a).

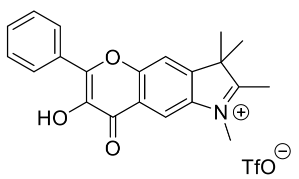

Flavonol **11a** (180 mg, 0.56 mmol) was dissolved in dry CH_2Cl_2 (5 mL) under N_2 atmosphere. Methyl trifluoromethanesulfonate (139 mg, 96 µL, 0.845 mmol) was added dropwise, and the mixture was stirred for 16 h at rt. Afterward, precipitate was filtered, washed with CH_2Cl_2 (2 × 2 mL) and dried to provide **12a**. Yield: 235 mg (86%). Beige solid. Mp. 273.1−275.6 °C. ^{1}H NMR (500 MHz, d_6–DMSO): δ (ppm) 9.90 (brs, 1H, OH), 8.53 (s, 1H), 8.36 (S, 1H), 8.24 (d, J = 7.7 Hz, 2H), 7.63–7.51 (m, 3H), 4.10 (s, 3H), 2.82 (s, 3H), 1.62 (s, 6H). ^{13}C NMR (125 MHz, d_6–DMSO): δ (ppm) 197.1, 172.6, 154.8, 146.5, 145.8, 139.11, 139.06, 130.9, 130.2, 128.6, 127.6, 121.9, 121.4, 119.4, 114.6, 111.2, 54.1, 35.1, 21.9, 14.3. ^{19}F NMR (282 MHz, d_6–DSMO): δ (ppm) –77.71 (s). HRMS (ESI+): calcd. for $C_{21}H_{20}NO_3^+$ [M$^+$] 334.1438, found 334.1440.

7-Hydroxy-1,2,3,3-tetramethyl-8-oxo-6-(4-(trimethylammonio)phenyl)-3,8-dihydropyrano[2,3-*f*]indol-1-ium Trifluoromethanesulfonate (12b).

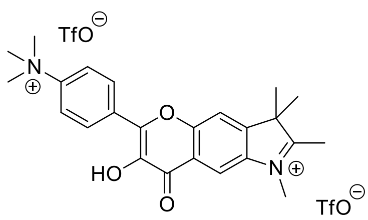

Flavonol **11b** (166 mg, 0.46 mmol) was dissolved in acetonitrile (15 mL) and CH_3I (0.71 mL, 11.5 mmol) was added. The mixture was heated in a glass pressure tube at 100 °C for 4 h. After cooling down to rt, the resulting precipitate was filtered, washed with acetonitrile (3 × 2 mL) and diethyl ether (3 × 2 mL). The precipitate (236 mg, 0.37 mmol) was subsequently redissolved in methanol (40 mL). AgOTf (188 mg, 0.73 mmol) in methanol (1 mL) was added dropwise, and the mixture was stirred for 1 h. The resulting precipitate was filtered off, washed with methanol (5 mL) and discarded. The filtrate was collected, and the solvent was evaporated at reduced pressure to afford **12b** in a mixture with its tautomer in variable ratios. The equilibrium can be shifted toward **12b** by the addition of H_2SO_4 (Figure S14–S17). The hydrogens of the methyl group were promptly exchanged for deuteriums in CD_3OD. Yield: 250 mg (81%) in 2 steps.

Beige solid. Mp. 286.1−290.3 °C. ^{1}H NMR (500 MHz, d_6–DMSO): δ (ppm) 10.31 (brs, 1H, OH), 8.55 (s, 1H), 8.42 (d, J = 8.8 Hz, 2H), 8.39 (s, 1H), 8.20 (d, J = 8.8 Hz, 2H), 4.11 (s, 3H), 3.69 (s, 9H), 2.84 (s, 3H), 1.63 (s, 6H). ^{13}C NMR (125 MHz, d_6–DSMO): δ (ppm) 197.3, 172.8, 154.9, 147.6, 146.7, 143.8, 139.9, 139.2, 132.5, 129.0, 121.5, 120.9, 114.7, 111.3, 56.4, 54.1, 35.2, 21.9, 14.5. ^{19}F NMR (282 MHz, d_6–DSMO): δ (ppm) –77.71 (s). HRMS (ESI+): calcd. for $C_{24}H_{27}N_2O_3^+$ [M$^+$] 391.2016, found 391.2019.

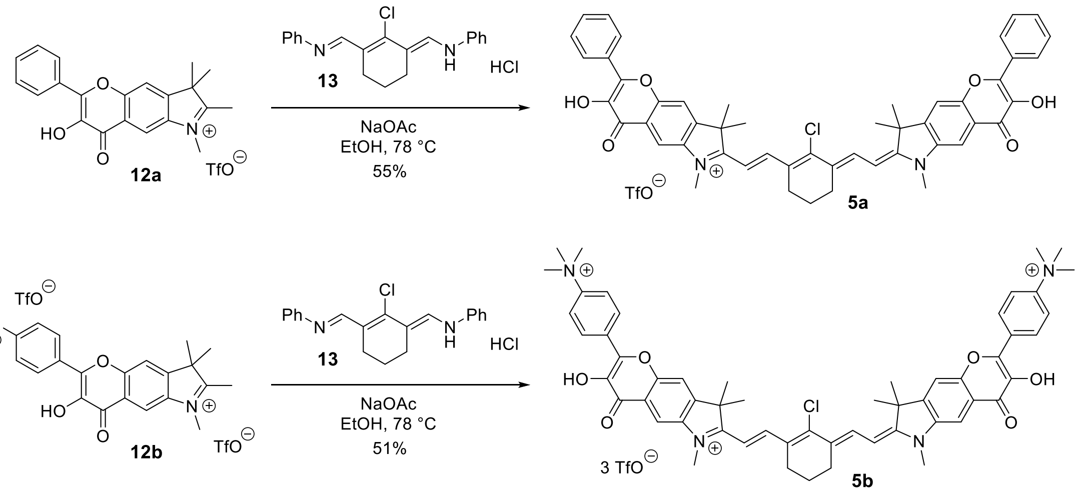

Scheme 2. Preparation of the cyanine-flavonol hybrids **5a** and **5b.**

2-((*E*)-2-((*E*)-2-Chloro-3-((*E*)-2-(7-hydroxy-1,3,3-trimethyl-8-oxo-6-phenyl-3,8-dihydropyrano[2,3-*f*]indol-2(1*H*)-ylidene)ethylidene)cyclohex-1-en-1-yl)vinyl)-7-hydroxy-1,3,3-trimethyl-8-oxo-6-phenyl-3,8-dihydropyrano[2,3-*f*]indol-1-ium Trifluoromethanesulfonate (5a).

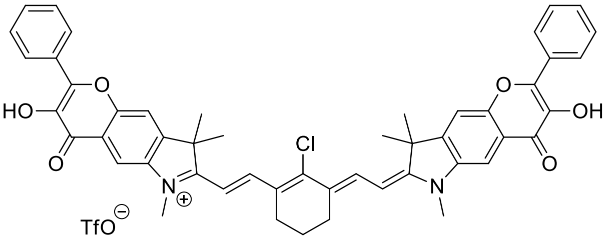

A mixture of indolenine **12a** (108 mg, 0.22 mmol), **13** (40 mg, 0.11 mmol), and sodium acetate (27 mg, 0.33 mmol) in ethanol (2 mL) was heated to reflux for 2 h. The reaction mixture was cooled down to rt, the precipitate was filtered off, washed with water (3 × 1 mL), diethyl ether (2 × 1 mL), methanol (2 × 1 mL), and acetone (1 mL) and dried on air to give the final product **5a**. Yield: 58 mg (55%). Dark green solid. Mp. 254.9−258.1 °C (decomp.). ^{1}H NMR (500 MHz, CD_2Cl_2): δ (ppm) 8.49 (d, J = 14.1 Hz, 2H), 8.29 (d, J = 7.4 Hz, 4H), 7.89 (s, 2H), 7.69 (s, 2H), 7.61−7.52 (m, 6H), 7.03 (s, 2H), 6.18 (d, J = 14.0 Hz, 2H), 3.72 (s, 6H), 2.73 (m, 4H), 2.00 (m, 2H), 1.85 (s, 12H). ^{13}C NMR (125 MHz, CD_2Cl_2): δ (ppm) 173.3, 173.1, 154.5, 152.9, 148.2, 145.9, 145.6, 140.7, 138.9, 131.3, 131.1, 129.31, 129.29, 128.2, 121.5, 114.0, 105.5, 102.3, 50.0, 32.6, 28.6, 26.9, 21.2. ^{19}F NMR (282 MHz, CD_2Cl_2): –78.88 (s). HRMS (ESI+): calcd. for $C_{50}H_{44}ClN_2O_6^+$ [M$^+$] 803.2882, found 803.2879.

2-((*E*)-2-((*E*)-2-Chloro-3-((*E*)-2-(7-hydroxy-1,3,3-trimethyl-8-oxo-6-(4-(trimethylammonio)phenyl)-3,8-dihydropyrano[2,3-*f*]indol-2(1*H*)-ylidene)ethylidene)cyclohex-1-en-1-yl)vinyl)-7-hydroxy-1,3,3-trimethyl-8-oxo-6-(4-(trimethylammonio)phenyl)-3,8-dihydropyrano[2,3-*f*]indol-1-ium Trifluoromethanesulfonate (5b).

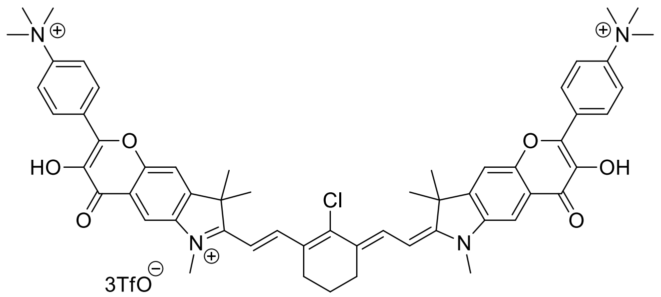

A mixture of indolenine **12b** (252 mg, 0.36 mmol), **13** (66 mg, 0.18 mmol), and sodium acetate (45 mg, 0.55 mmol) in methanol (5 mL) was heated to reflux for 2 h. The reaction mixture was cooled down to rt, the precipitate was filtered off, washed with diethyl ether (2 × 2 mL), water (3 × 2 mL), methanol (2 mL), acetone (2 ml), and CH_2Cl_2 (2 × 2 mL) and dried on air to give the final product **5b**. Yield: 128 mg (51%). A dark green solid with red metallic gloss. Mp. 261.1−263.8 °C (decomp.). ^{1}H NMR (300 MHz, d_6–DMSO): δ (ppm) 10.12 (brs, 2H, OH), 8.42 (d, J = 9.3 Hz, 4H), 8.31 (d, J = 14.1 Hz, 2H), 8.18 (d, J = 9.3 Hz, 4H), 8.18 (s, 2H), 7.96 (s, 2H), 6.40 (d, J = 14.2 Hz, 4H), 3.82 (s, 6H), 3.68 (s, 18H), 2.76 (m, 4H), 1.90 (m, 2H), 1.79 (s, 12H). ^{13}C NMR (125 MHz, d_6–DMSO): δ (ppm) 172.6, 172.2, 153.1, 148.4, 147.8, 147.5, 143.2 (2 × C based on HSQC and HMBC), 140.3, 139.7, 132.7, 128.8, 127.7, 121.9 (TfO^-), 121.6, 120.9, 119.4 (TfO^-), 113.6, 150.1, 102.6, 56.4, 49.0, 32.1, 27.3, 25.9, 20.3 (from HSQC). ^{19}F NMR (282 MHz, d_6–DMSO): –77.71 (s). HRMS (ESI+): calcd. for $C_{56}H_{60}ClN_4O_6^{3+}$ [M^{3+}] 306.4728, found 306.4729.

2-(4-(Dimethylamino)phenyl)-3-hydroxy-4*H*-benzo[*g*]chromen-4-one (17).

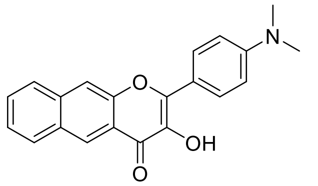

NaOH (1.7 g, 43 mmol) in water (5 mL) was added to a solution of 1-(3-hydroxynaphthalen-2-yl)ethan-1-one[9] (2.0 g, 10.7 mmol) and 4-(dimethylamino)benzaldehyde (2.1 g, 13.9 mmol) in methanol (30 mL). The resulting mixture was heated to 50 °C and stirred for 30 min. The solvents were evaporated at reduced pressure, and the residue was redissolved in methanol (30 mL). This process was repeated twice to shift the equilibrium toward the aldol product. Afterward, the mixture was cooled to 0 °C, and hydrogen peroxide (30%, 3.1 mL, 29.0 mmol) was added dropwise. The reaction mixture was stirred for 16 h, followed by neutralization with aq. HCl (pH = 7). The resulting precipitate was filtered off, washed with water (20 mL) and methanol (2 × 15 mL) to afford flavonol **17**. Yield: 3.0 g (84%). Yellow powder. Mp. 219–221 °C. ^{1}H NMR (500 MHz, d_6-DMSO): δ (ppm) 9.13 (s, 1H), 8.78 (s, 1H), 8.22 (s, 1H), 8.20–8.25 (m, 3H), 8.06 (d, 1H, J = 8.4 Hz), 7.65 (dd, 1H, J_1 = 7.5 Hz, J_2 = 7.5 Hz), 7.55 (dd, 1H, J_1 = 7.4 Hz, J_2 = 7.4 Hz), 6.87 (d, 2H, J = 9.1 Hz), 3.04 (s, 6H). ^{13}C NMR (125 MHz, d_6-DMSO): δ (ppm) 172.6, 151.1, 150.7, 147.6, 136.0, 135.0, 129.3, 129.2 (2×C), 128.4, 127.1, 125.6, 125.4, 121.1, 118.0, 113.7, 111.4, 111.0, 39.6. HRMS (APCI): calcd. for $C_{21}H_{18}NO_3$ [M + H^+] 332.1281, found 332.1281.

4-(3-Hydroxy-4-oxo-4*H*-benzo[*g*]chromen-2-yl)-*N*,*N*,*N*-trimethylbenzenammonium trifluoromethansulfonate (14).

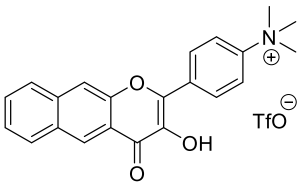

Flavonol **17** (0.49 g, 1.5 mmol) was dissolved in dry CH_2Cl_2 (10 mL) and stirred at 20 °C under N_2 atmosphere. Methyl trifluormethansulfonate (0.26 ml, 2.3 mmol) was added dropwise, and the reaction mixture was stirred at 20 °C for 16 h. The resulting precipitate was filtered off, washed with CH_2Cl_2 (3 × 8 mL), and dried on air to give flavonol **14**. Yield: 0.65 g (87%). Dark yellow powder. Mp. 240–242 °C. ^{1}H NMR (500 MHz, d_6-DMSO): δ (ppm) 9.97 (brs, 1H), 8.87 (s, 1H), 8.47 (d, 2H, J = 9.2 Hz), 8.34 (s, 1H), 8.27 (d, 1H, J = 8.4 Hz), 8.19 (d, 2H, J = 9.2 Hz), 8.09 (d, 1H, J = 8.4 Hz), 7.70 (dd, 1H, J_1 = 7.6 Hz, J_2 = 7.2 Hz), 7.59 (dd, 1H, J_1 = 7.5 Hz, J_2 = 7.5 Hz), 3.69 (s, 9H). ^{13}C NMR (125 MHz, d_6-DMSO): δ (ppm) 174.1, 150.8, 147.5, 143.9, 138.5, 135.5, 133.1, 129.44, 129.42, 129.16, 129.0, 127.1, 126.1, 126.0, 124.5 (TfO^-), 121.9 (TfO^-), 120.83, 120.80, 119.4 (TfO^-), 116.8 (TfO^-), 114.3, 56.4. HRMS (ESI+): calcd. for $C_{22}H_{20}NO_3^+$ [M^+] 346.1438, found 346.1440.

References

1. Martínek, M., Filipová, L., Galeta, J., Ludvíková, L. & Klán, P. Photochemical Formation of Dibenzosilacyclohept-4-yne for Cu-Free Click Chemistry with Azides and 1,2,4,5-Tetrazines. *Org. Lett.* **18,** 4892–4895 (2016).
2. James, N. S., Chen, Y., Joshi, P., Ohulchanskyy, T. Y., Ethirajan, M., Henary, M., Strekowski, L. & Pandey, R. K. Evaluation of polymethine dyes as potential probes for near infrared fluorescence imaging of tumors: Part - 1. *Theranostics* **3,** 692–702 (2013).
3. Shi, C., Wu, J. B. & Pan, D. Review on near-infrared heptamethine cyanine dyes as theranostic agents for tumor imaging, targeting, and photodynamic therapy. *J. Biomed. Opt.* **21,** 050901 (2016).
4. Young, R. H., Brewer, D. & Keller, R. A. Determination of Rate Constants of Reaction and Lifetimes of Singlet Oxygen in Solution by a Flash Photolysis Technique. *J. Am. Chem. Soc.* **95,** 375–379 (1973).
5. Russo, M., Štacko, P., Nachtigallová, D. & Klán, P. Mechanisms of Orthogonal Photodecarbonylation Reactions of 3-Hydroxyflavone-Based Acid–Base Forms. *J. Org. Chem.* **85,** 3527–3537 (2020).
6. Vreman, H. J., Kwong, L. K. & Stevenson, D. K. Carbon monoxide in blood: An improved microliter blood-sample collection system, with rapid analysis by gas chromatography. *Clin. Chem.* **30,** 1382–1386 (1984).
7. Balmond, E. I., Tautges, B. K., Faulkner, A. L., Or, V. W., Hodur, B. M., Shaw, J. T. & Louie, A. Y. Comparative Evaluation of Substituent Effect on the Photochromic Properties of Spiropyrans and Spirooxazines. *J. Org. Chem.* **81,** 8744–8758 (2016).
8. Potisek, S. L., Davis, D. A., Sottos, N. R., White, S. R. & Moore, J. S. Mechanophore-linked addition polymers. *J. Am. Chem. Soc.* **129,** 13808–13809 (2007).
9. Anderson, S. N., Richards, J. M., Esquer, H. J., Benninghoff, A. D., Arif, A. M. & Berreau, L. M. A Structurally-Tunable 3-Hydroxyflavone Motif for Visible Light-Induced Carbon Monoxide-Releasing Molecules (CORMs). *ChemistryOpen* **4,** 590–594 (2015).
10. Martínek, M., Filipová, L., Galeta, J., Ludvíková, L. & Klán, P. Photochemical Formation of Dibenzosilacyclohept-4-yne for Cu-Free Click Chemistry with Azides and 1,2,4,5-Tetrazines. *Org. Lett.* **18,** 4892–4895 (2016).

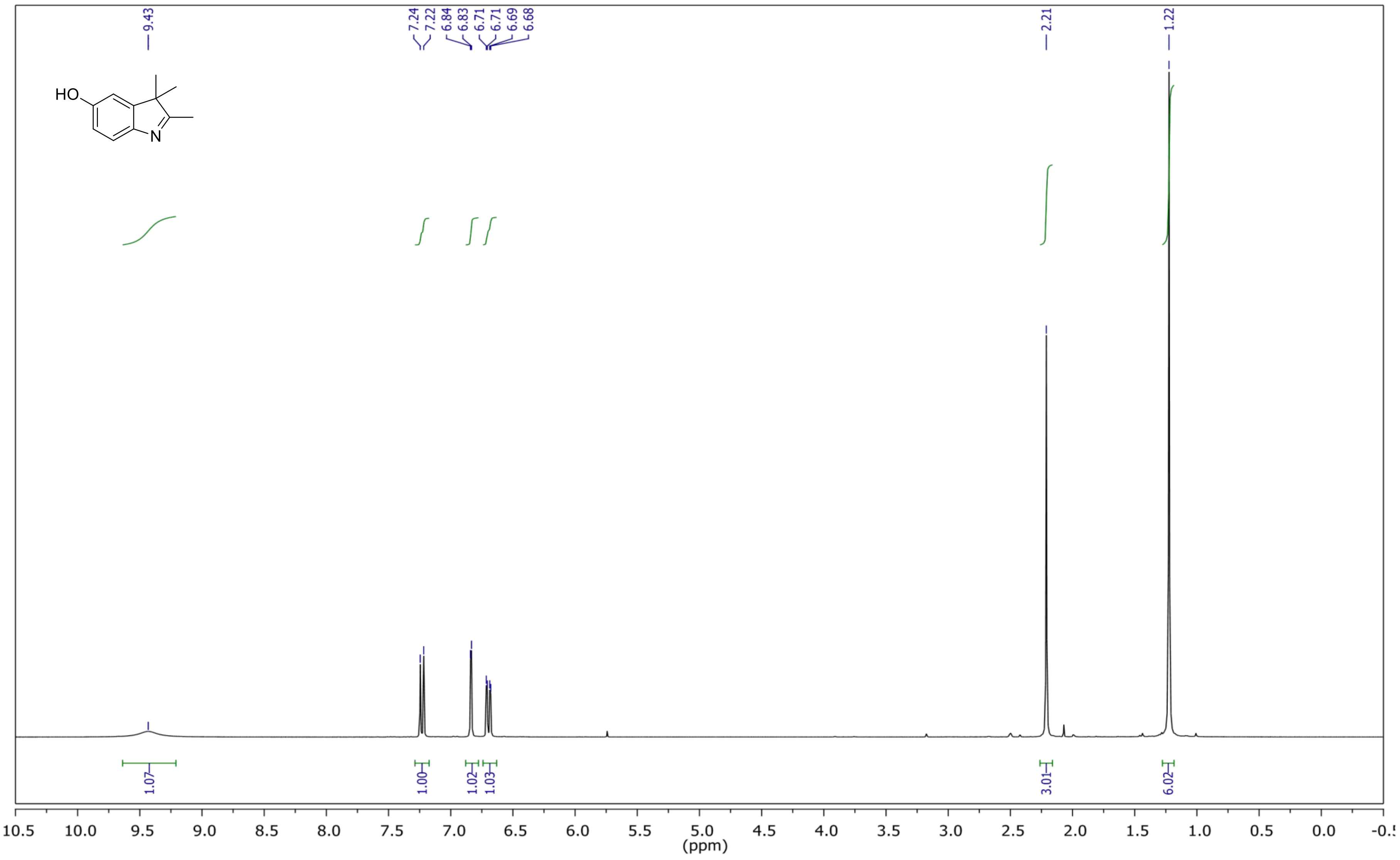

Figure S1. ^{1}H NMR (300 MHz, d_6–DMSO): **8**

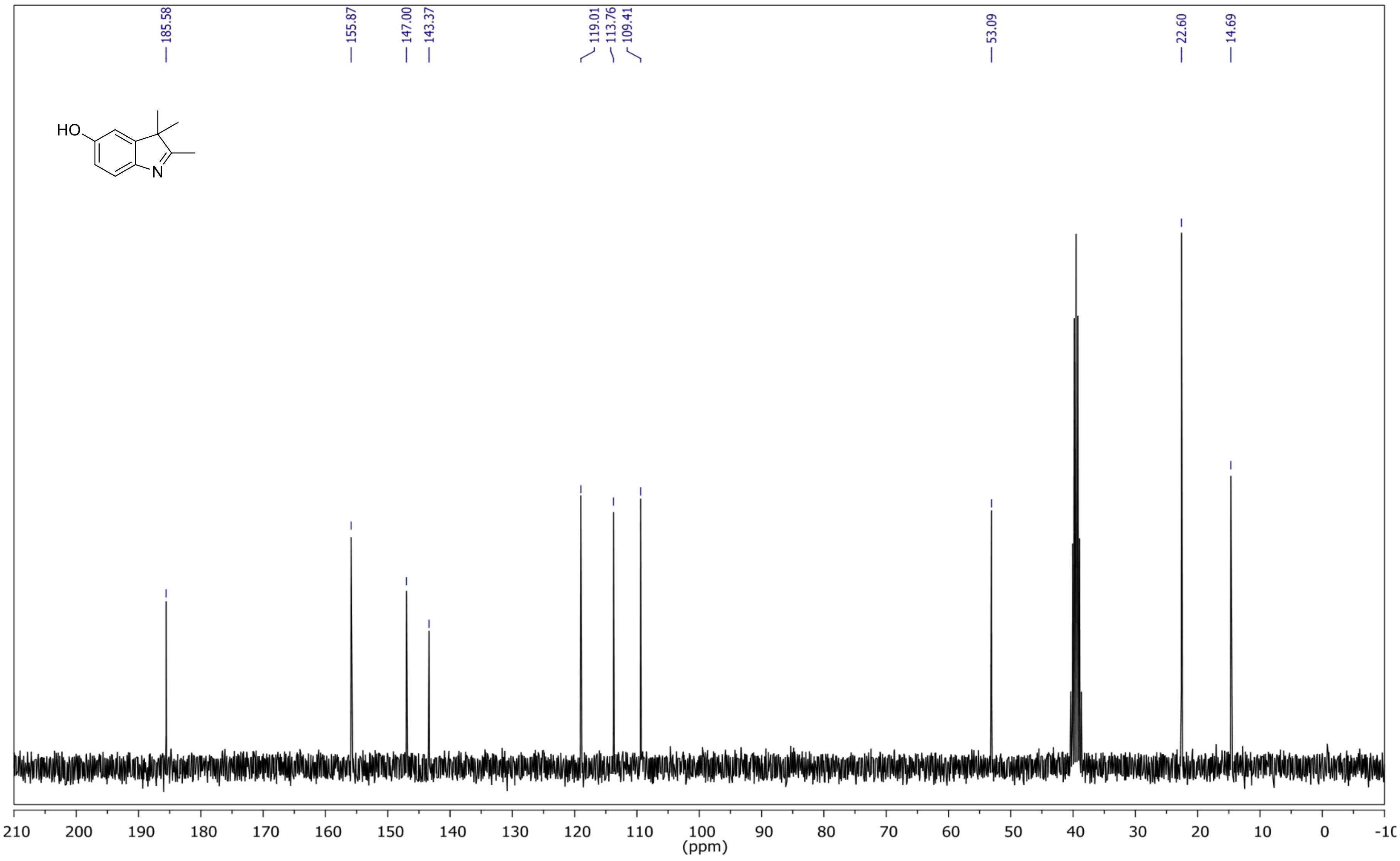

Figure S2. ^{13}C NMR (75 MHz, d_4–DMSO): **8**

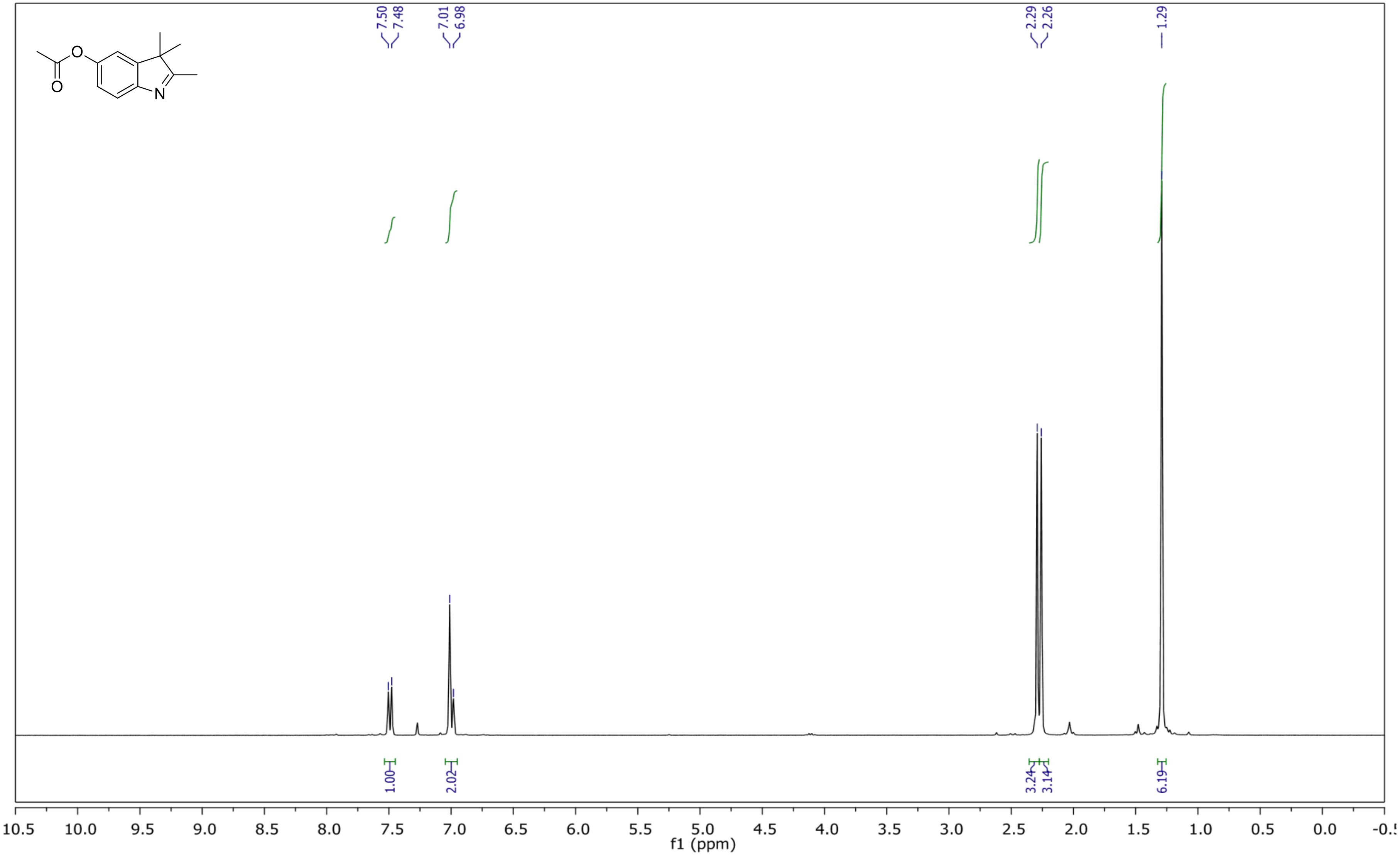

Figure S3. ^{1}H NMR (300 MHz, $CDCl_3$): **9**

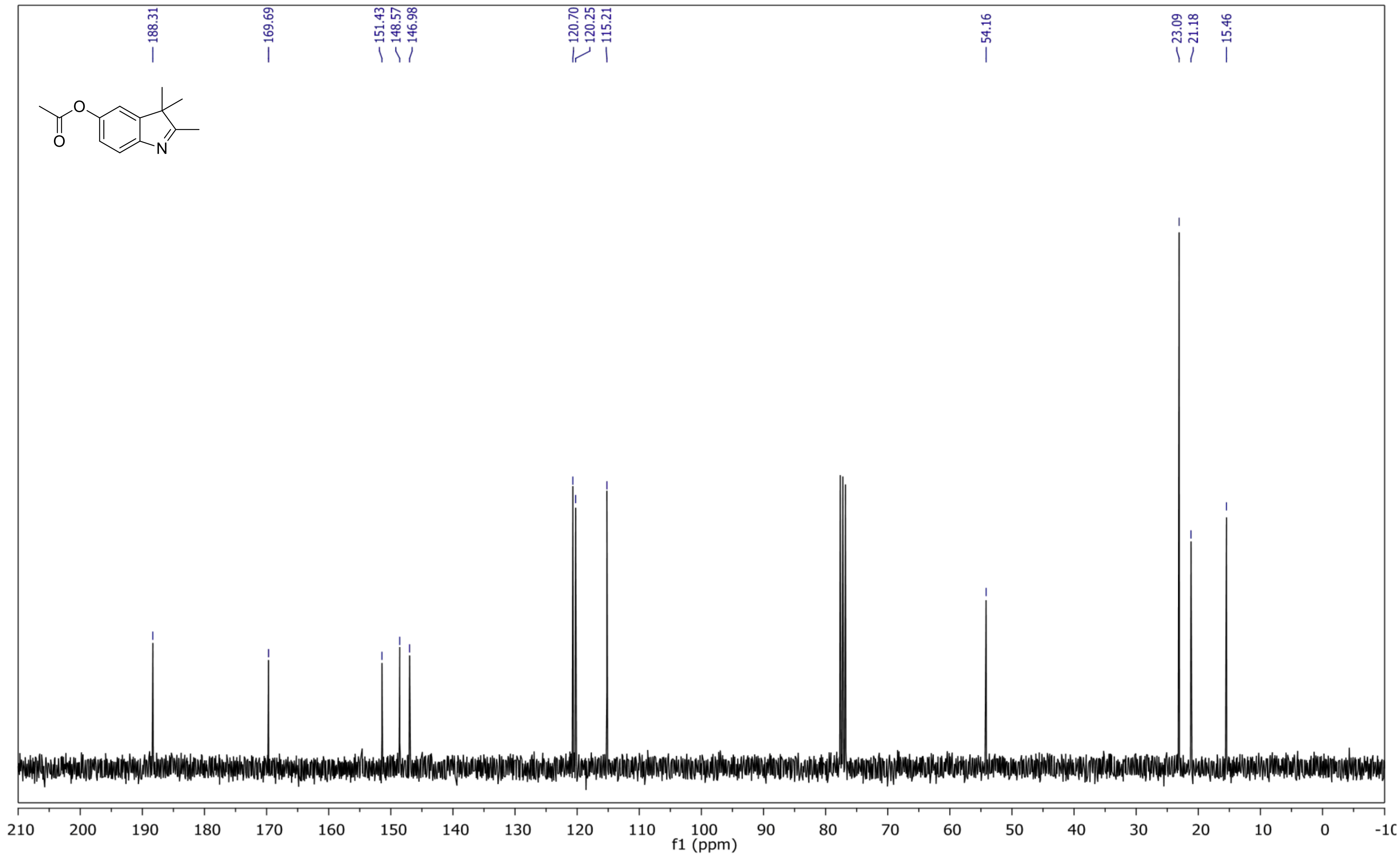

Figure S4. ^{13}C NMR (75 MHz, $CDCl_3$): **9**

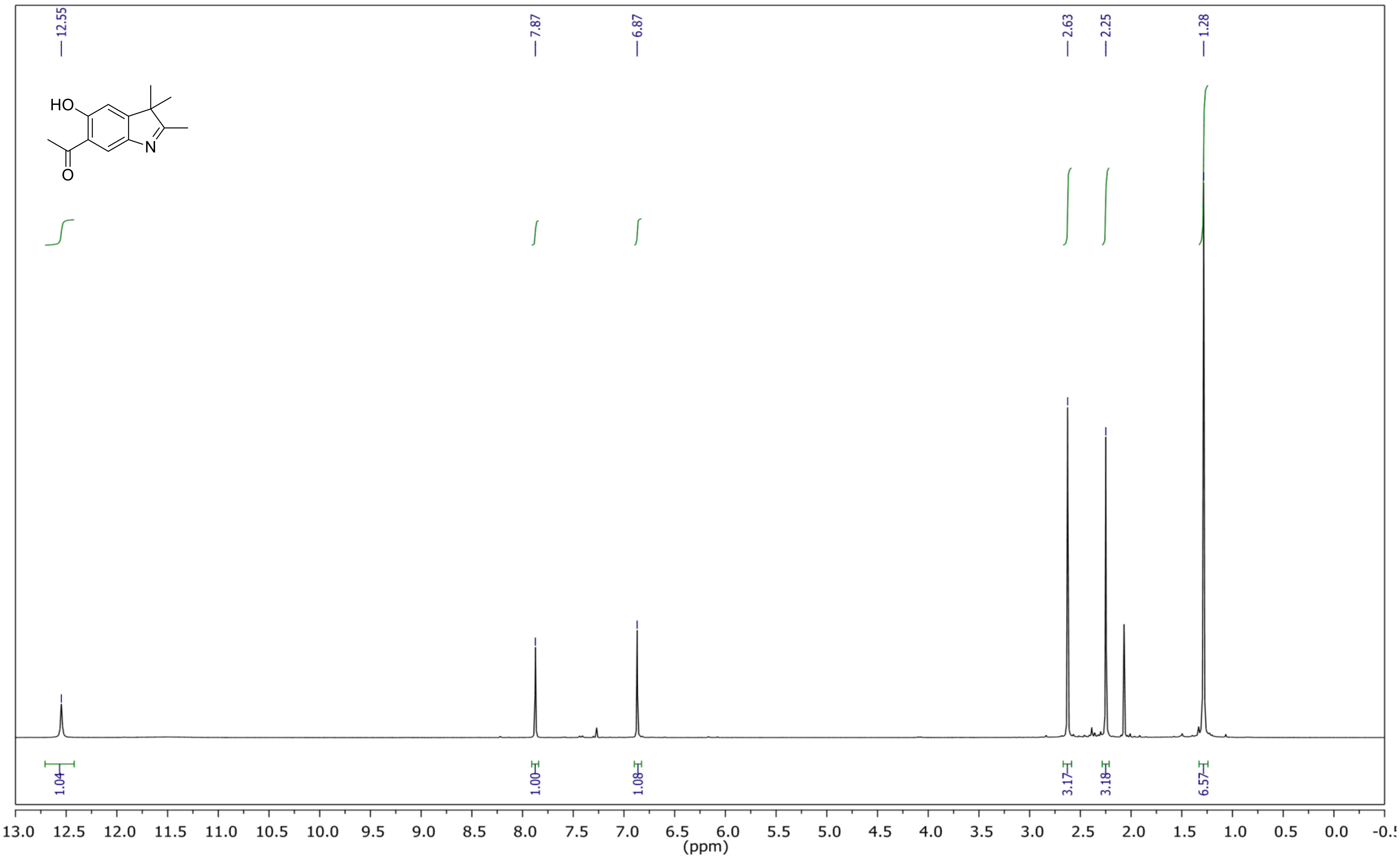

Figure S5. ^{1}H NMR (300 MHz, $CDCl_3$): **10**

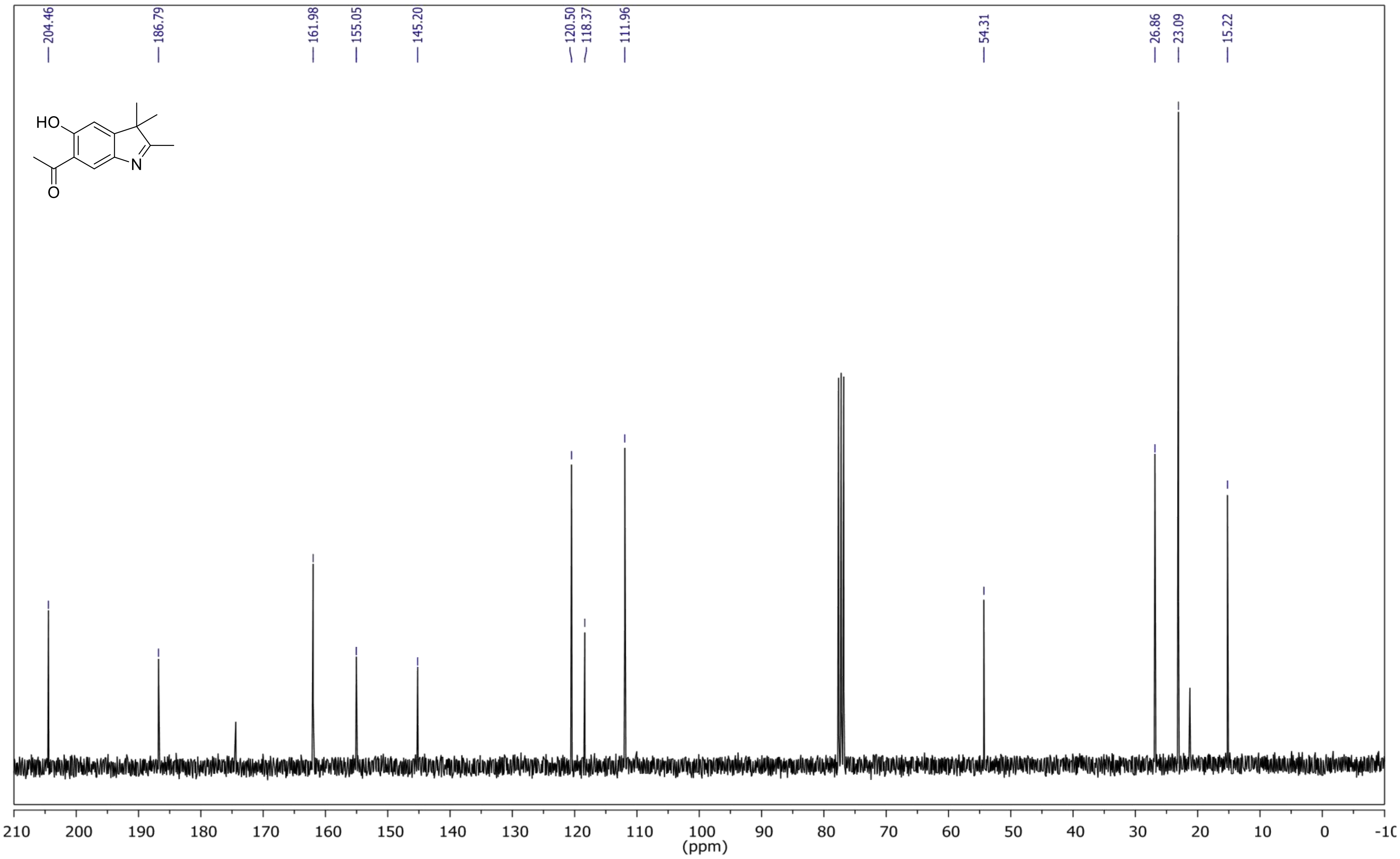

Figure S6. ^{13}C NMR (75 MHz, $CDCl_3$): **10**

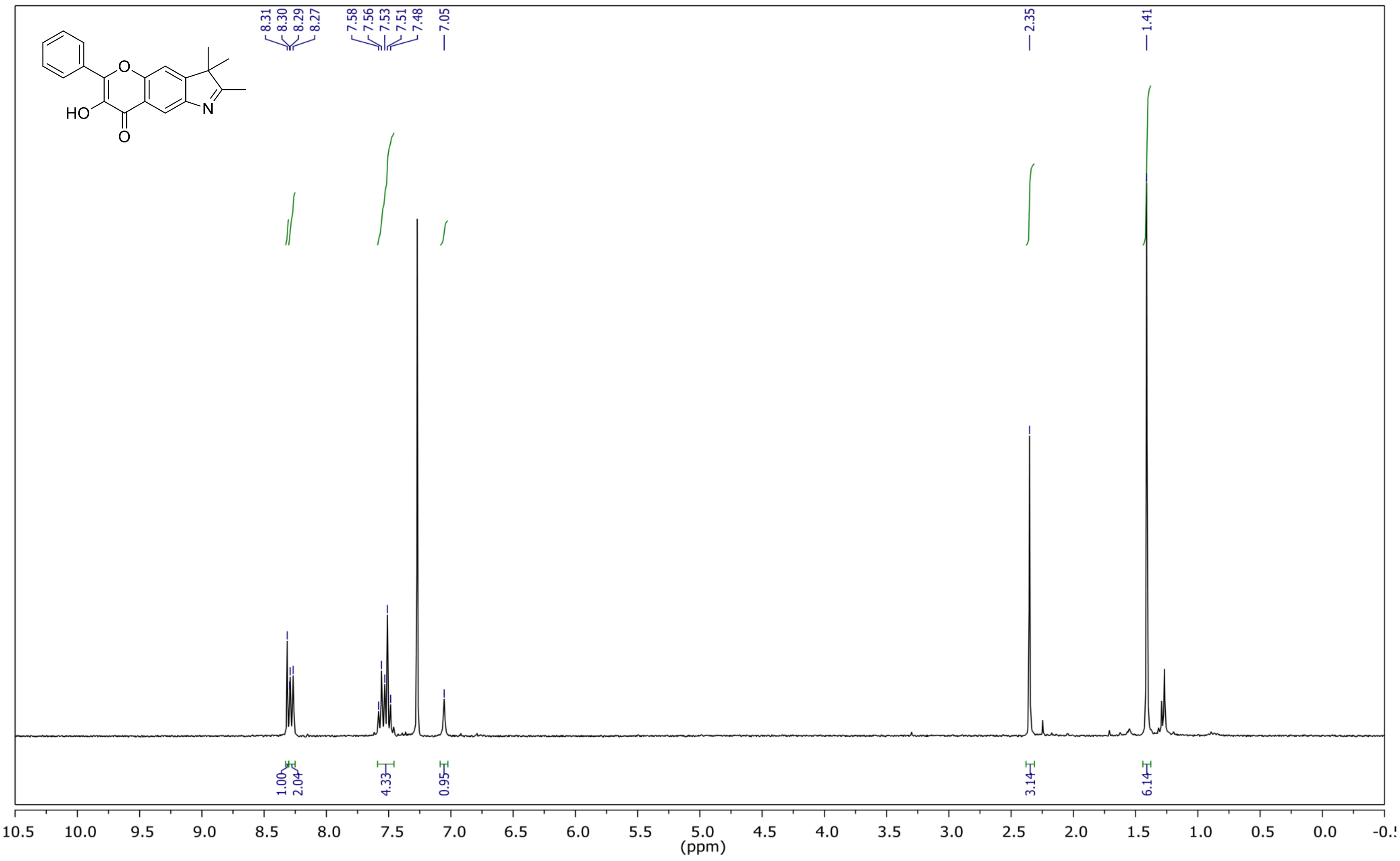

Figure S7. ^{1}H NMR (300 MHz, $CDCl_3$): **11a**

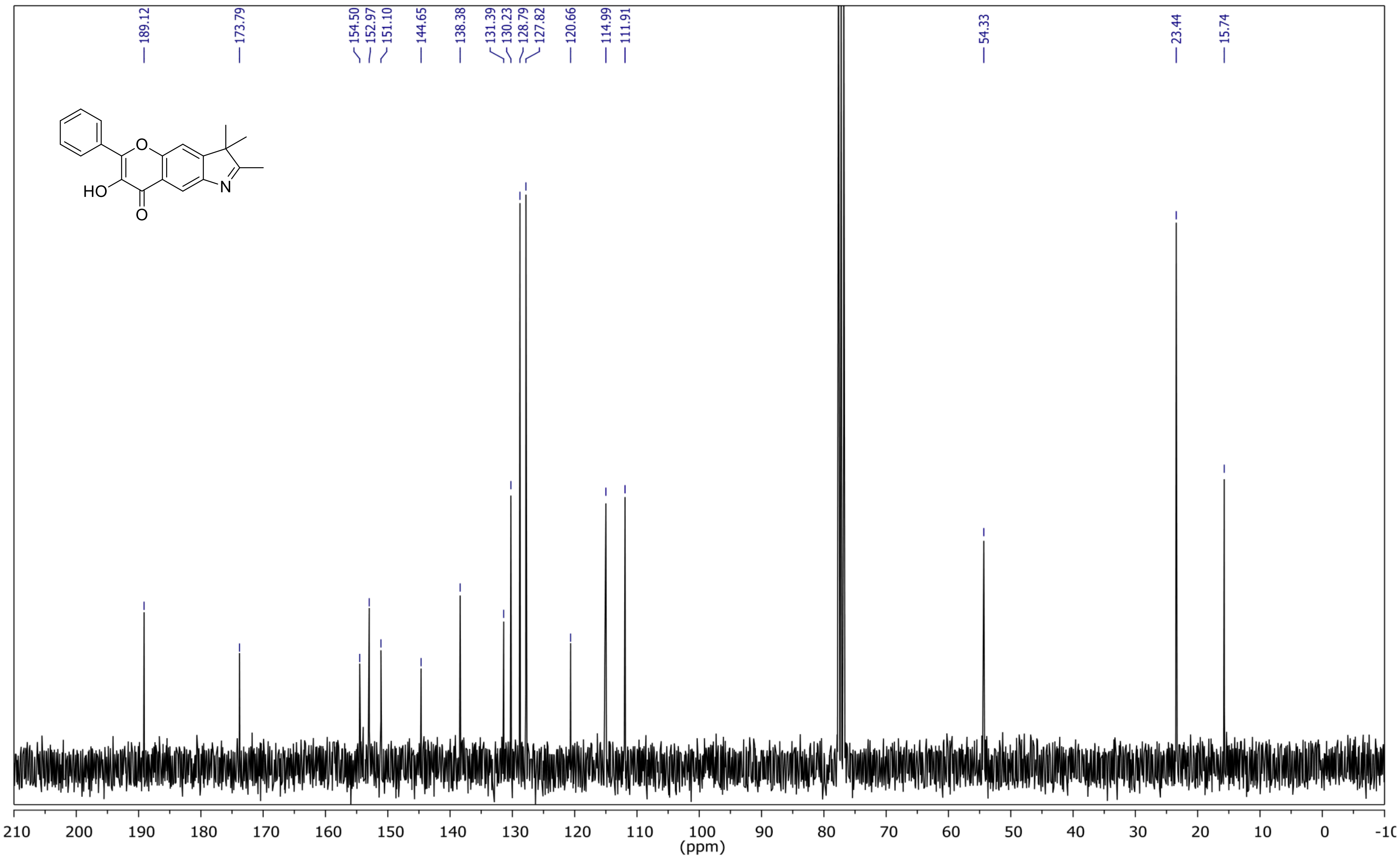

Figure S8. ^{13}C NMR (75 MHz, $CDCl_3$): **11a**

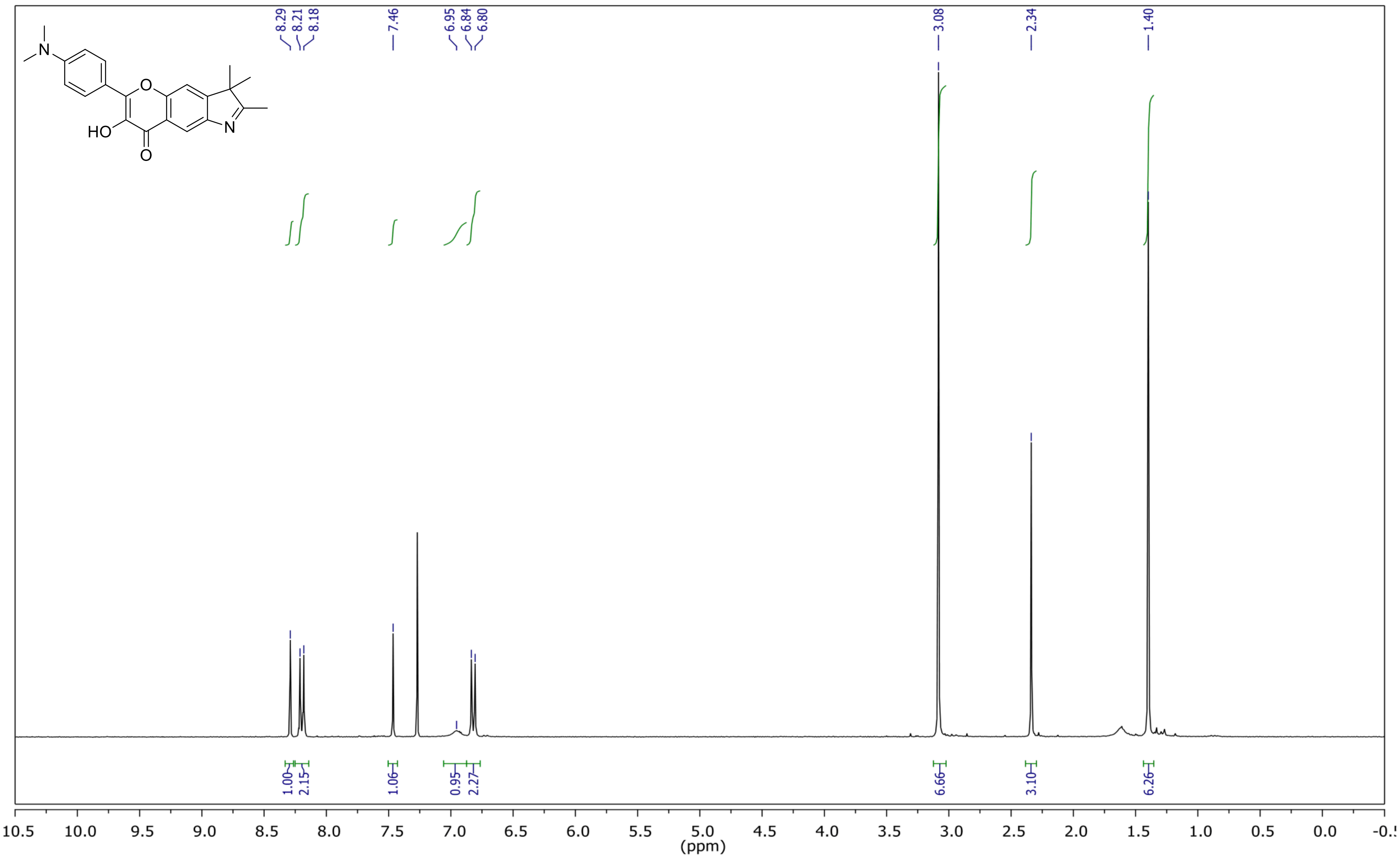

Figure S9. ^{1}H NMR (300 MHz, $CDCl_3$): **11b**

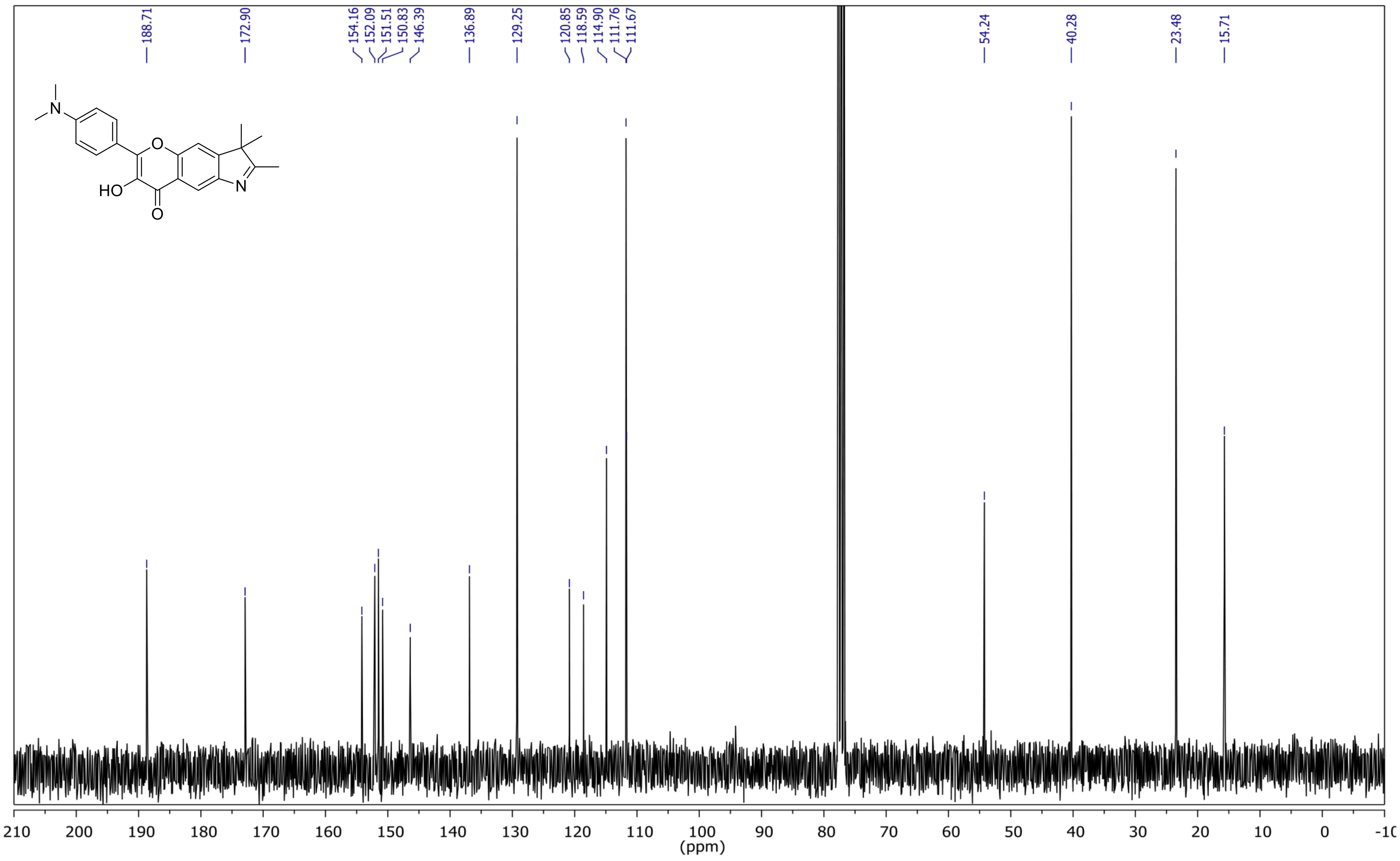

Figure S10. ^{13}C NMR (75 MHz, $CDCl_3$): **11b**

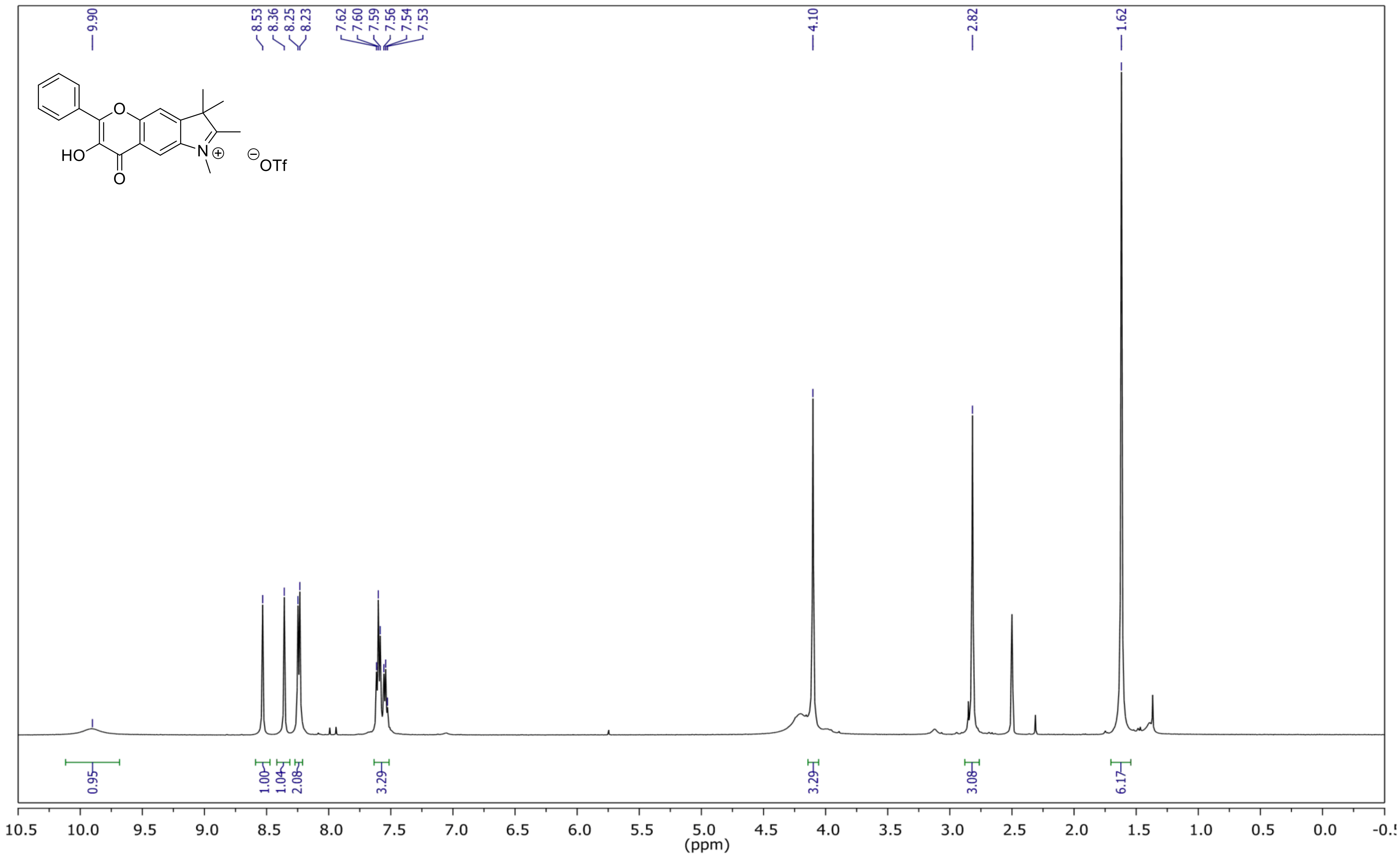

Figure S11. ^{1}H NMR (500 MHz, d_6–DMSO): **12a**

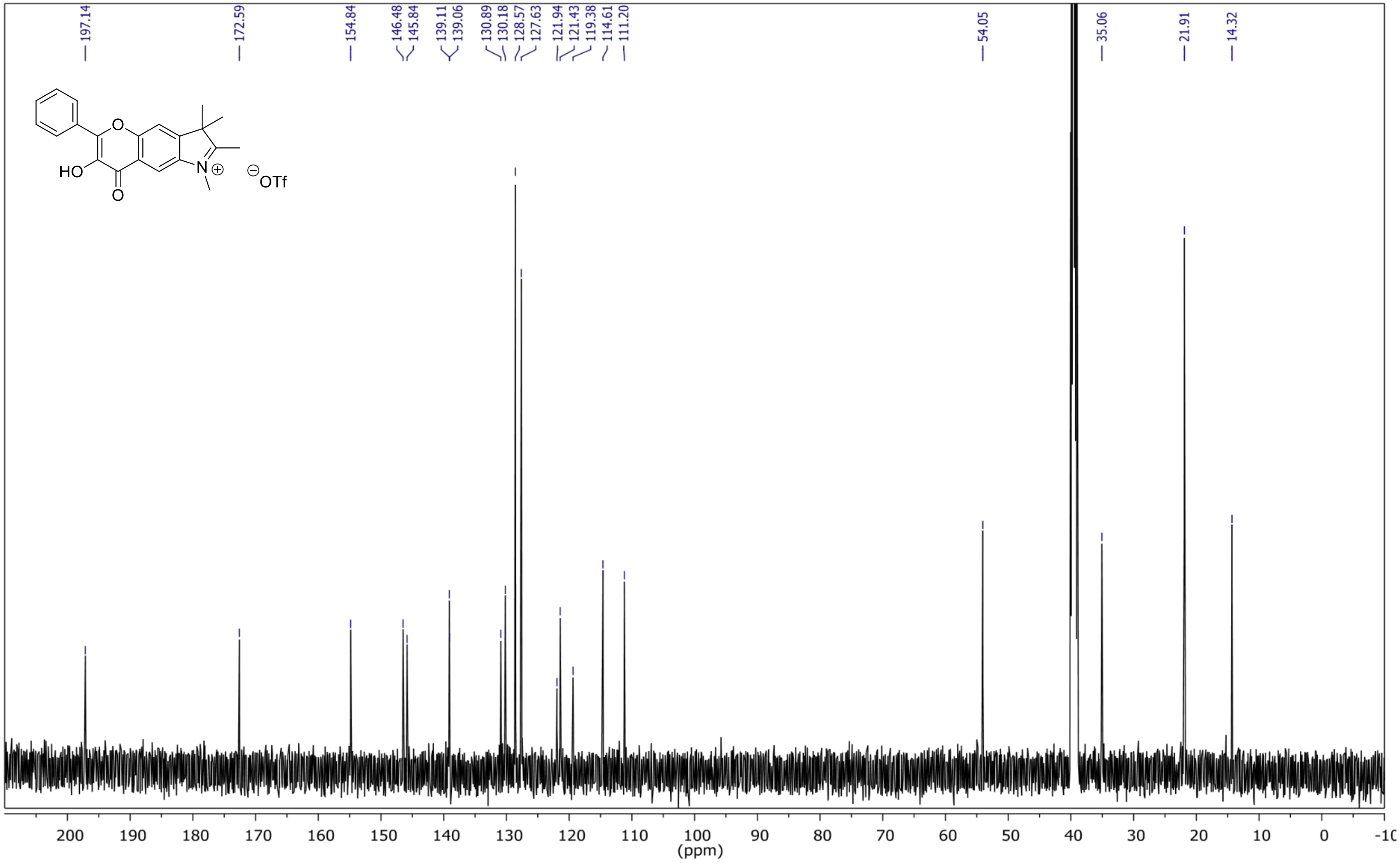

Figure S12. ^{13}C NMR (125 MHz, d_6–DMSO): **12a**

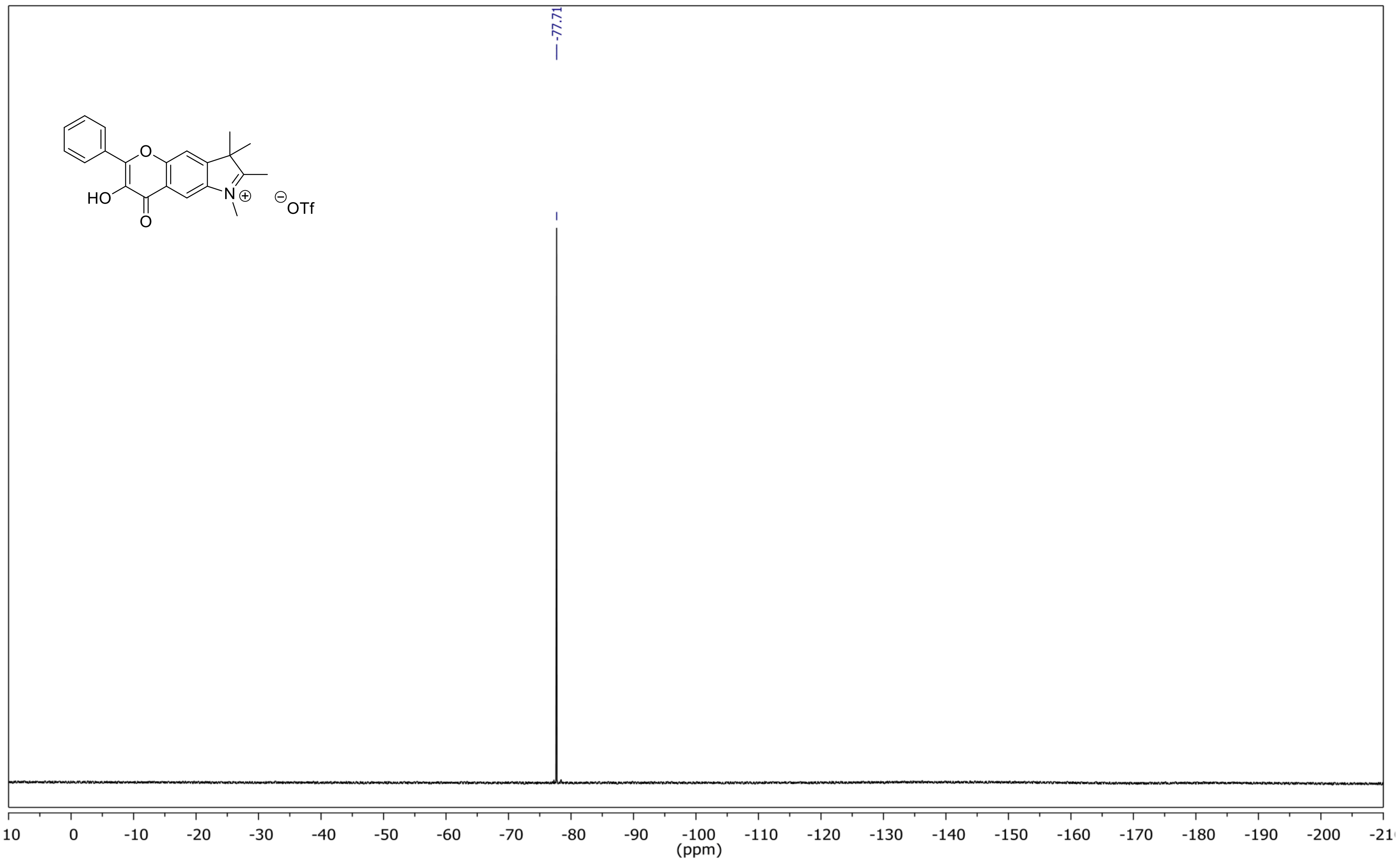

Figure S13. ^{19}F NMR (282 MHz, d_6–DMSO): **12a**

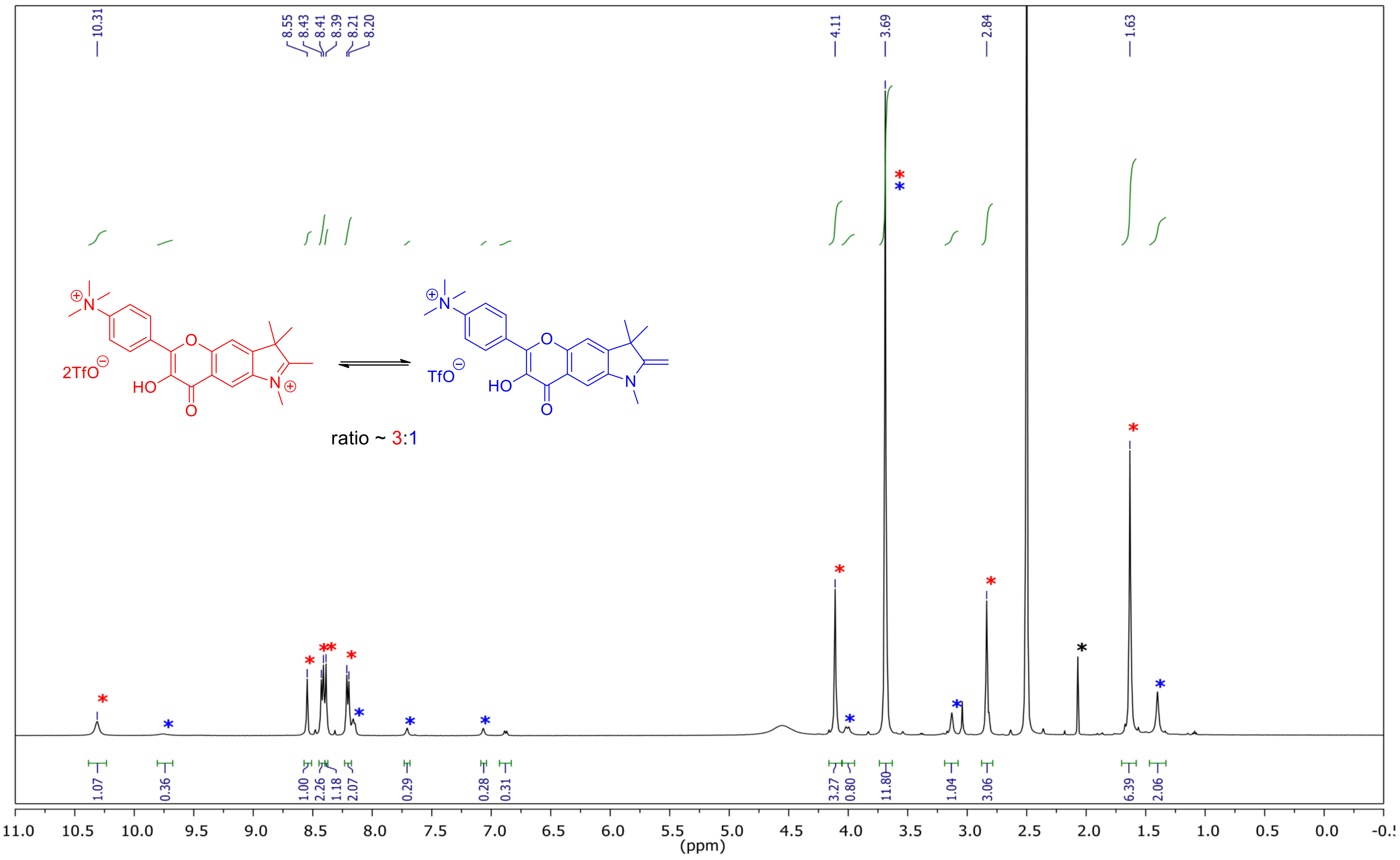

Figure S14. ^{1}H NMR (500 MHz, d_6–DMSO): **12b** and its tautomer (Black asterisk denotes residual CH_3CN in d_6–DMSO)

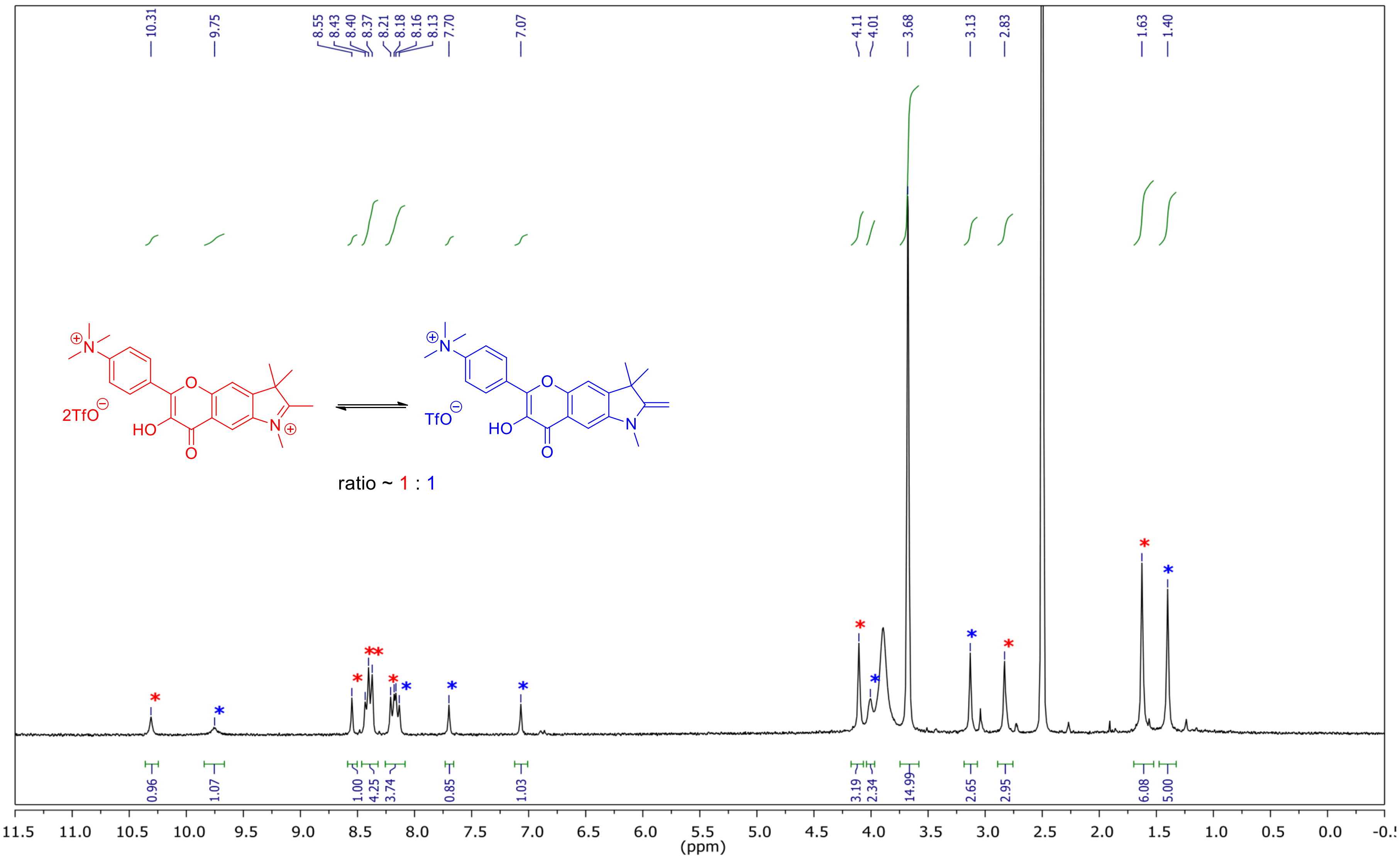

Figure S15. ^{1}H NMR (300 MHz, d_6–DMSO): **12b** and its tautomer

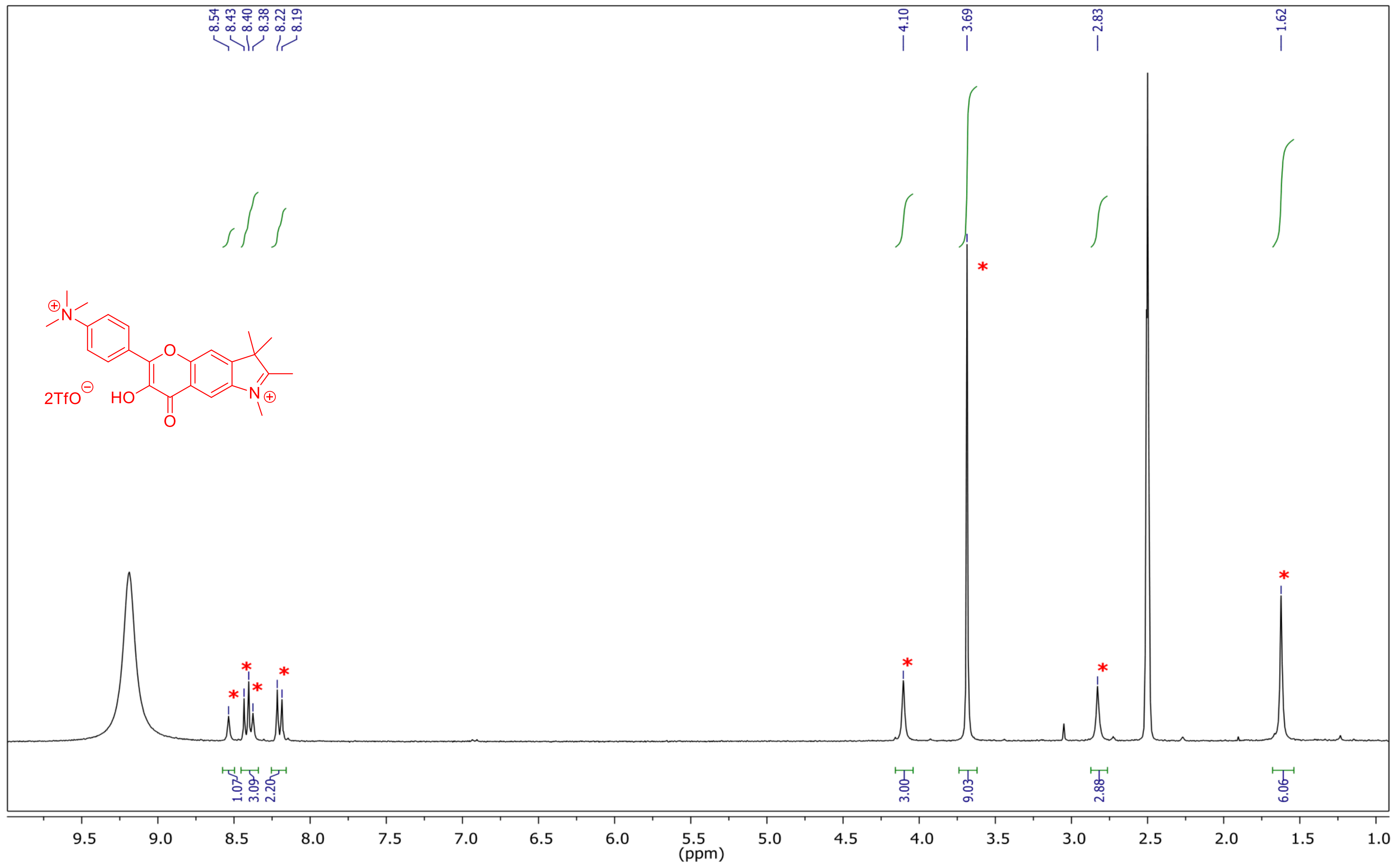

Figure S16. ^{1}H NMR (300 MHz, d_6–DMSO + 1µL H_2SO_4): **12b**

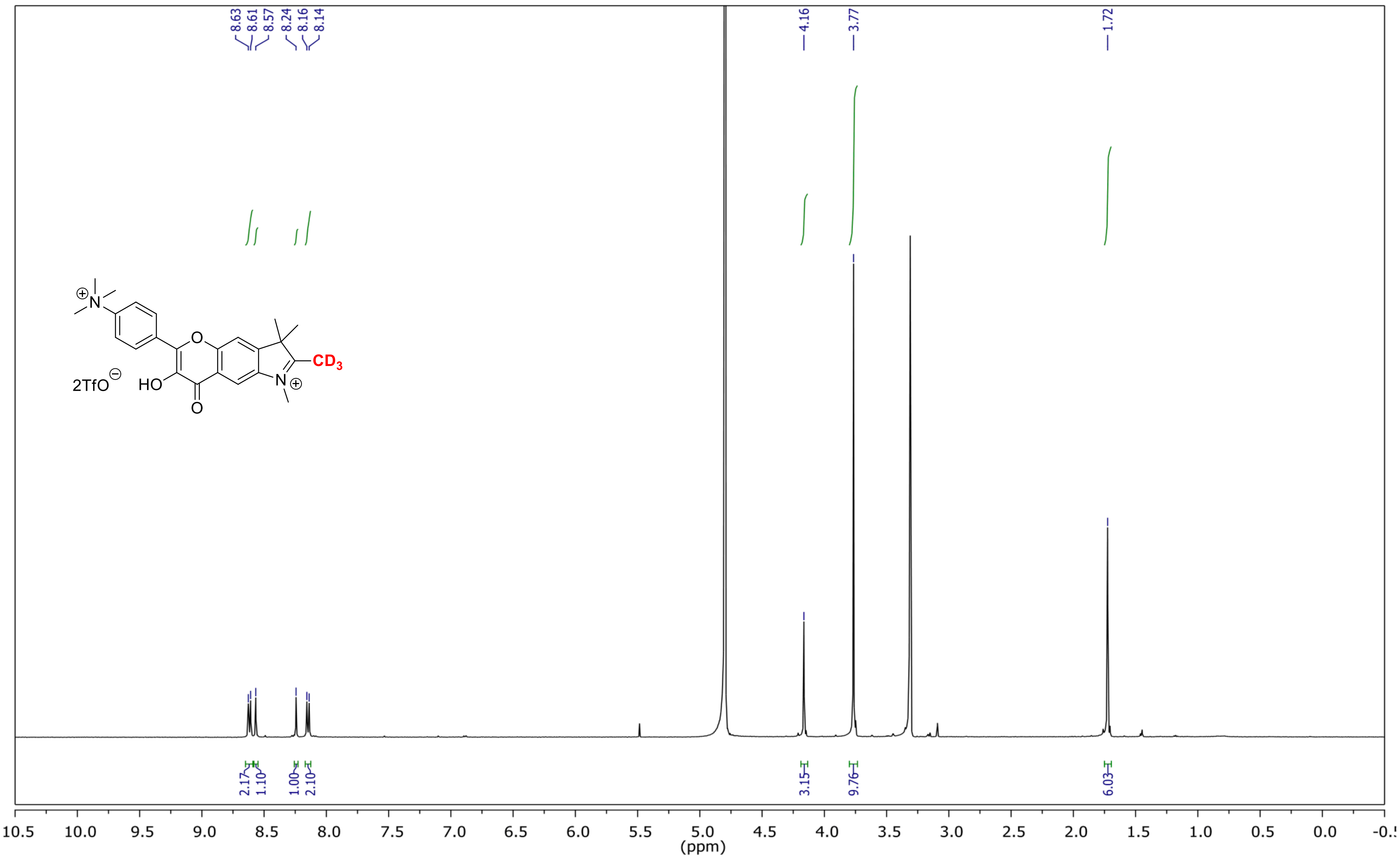

Figure S17. ^{1}H NMR (300 MHz, CD_3OD): d_3-**12b** produced in deuterated methanol

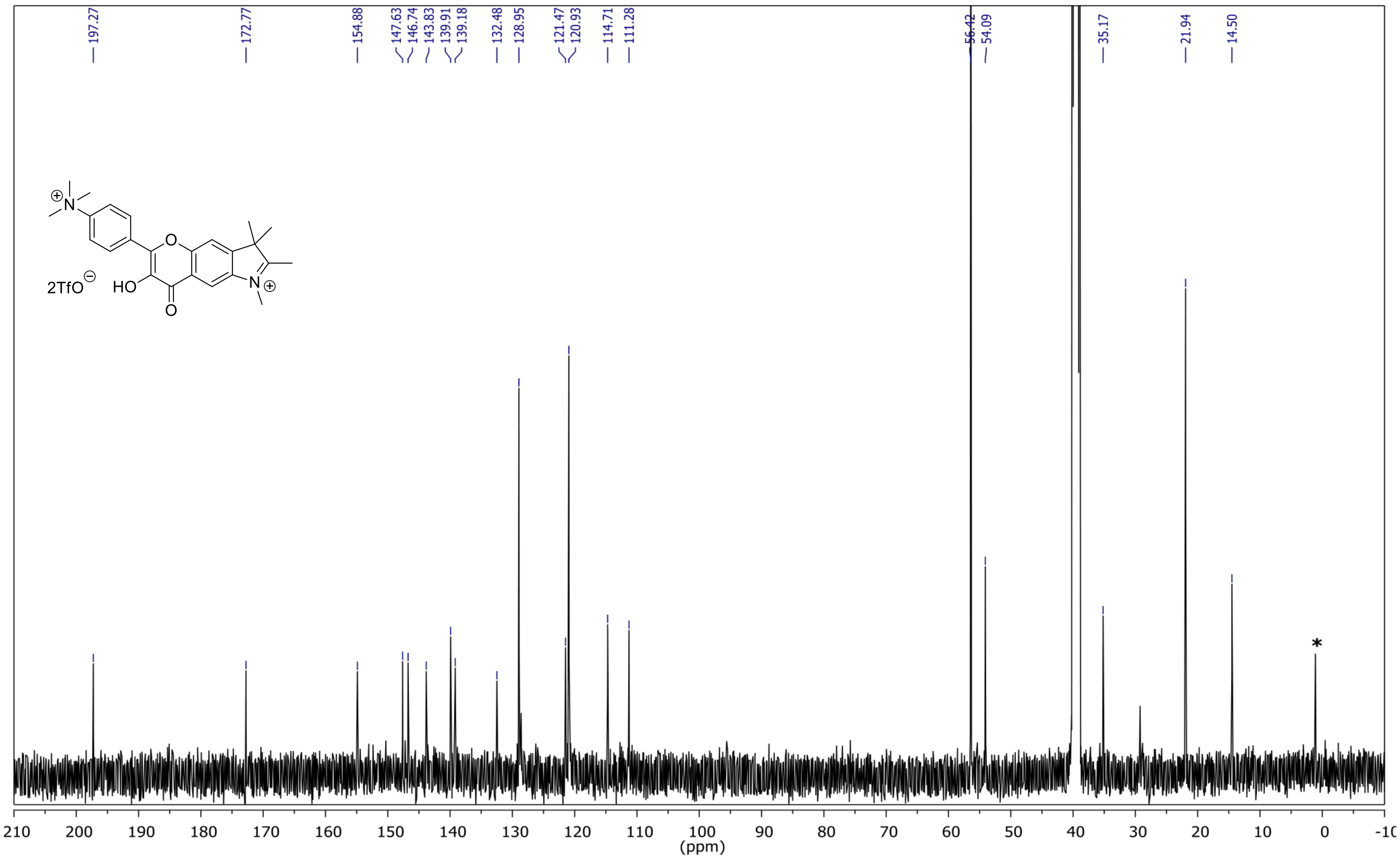

Figure S18. ^{13}C NMR (125 MHz, d_6–DMSO): **12b** (a black asterisk denotes residual CH_3CN in d_6–DMSO)

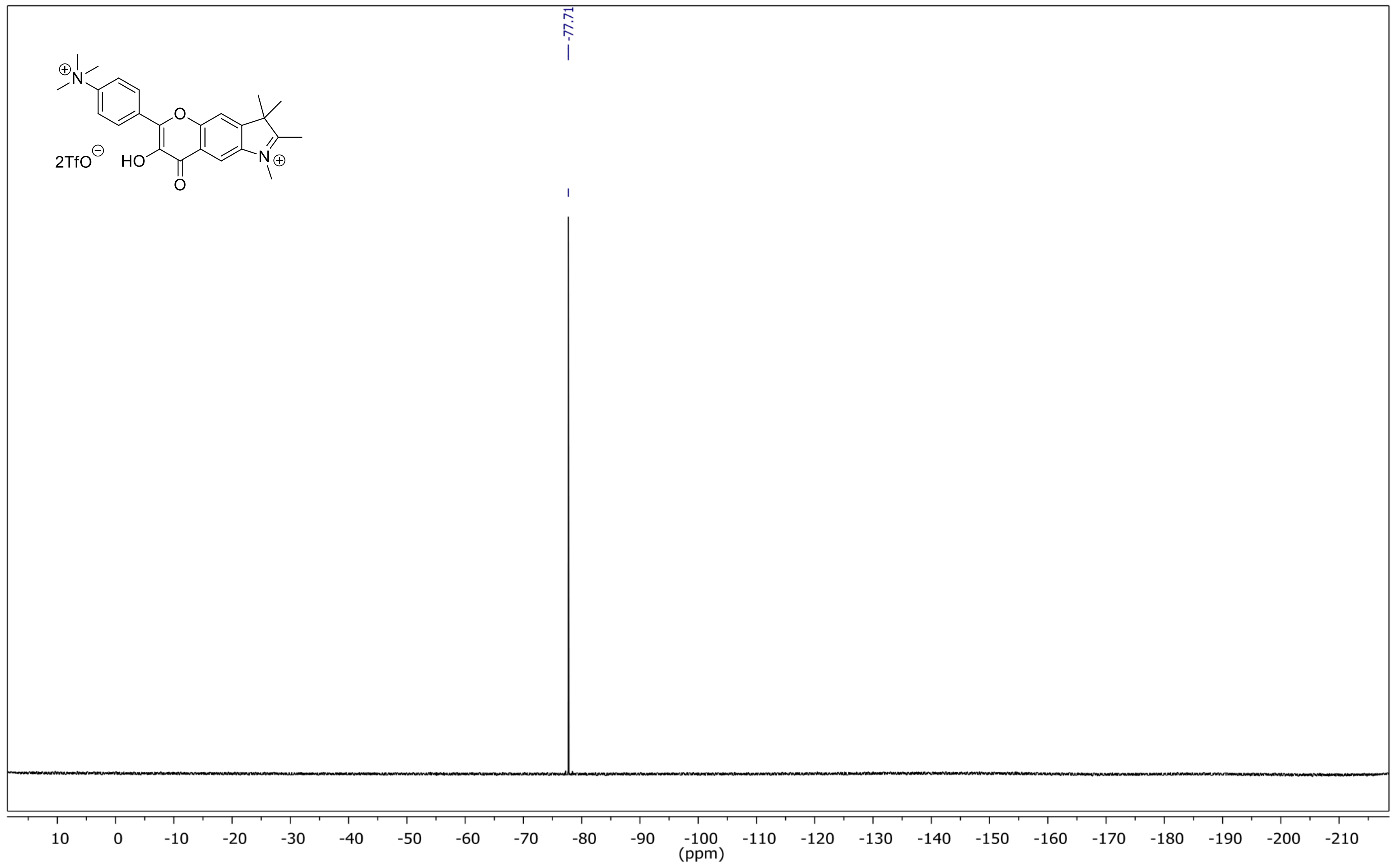

Figure S19. ^{19}F NMR (282 MHz, d_6–DMSO): **12b**

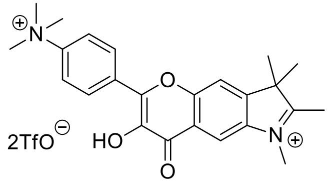

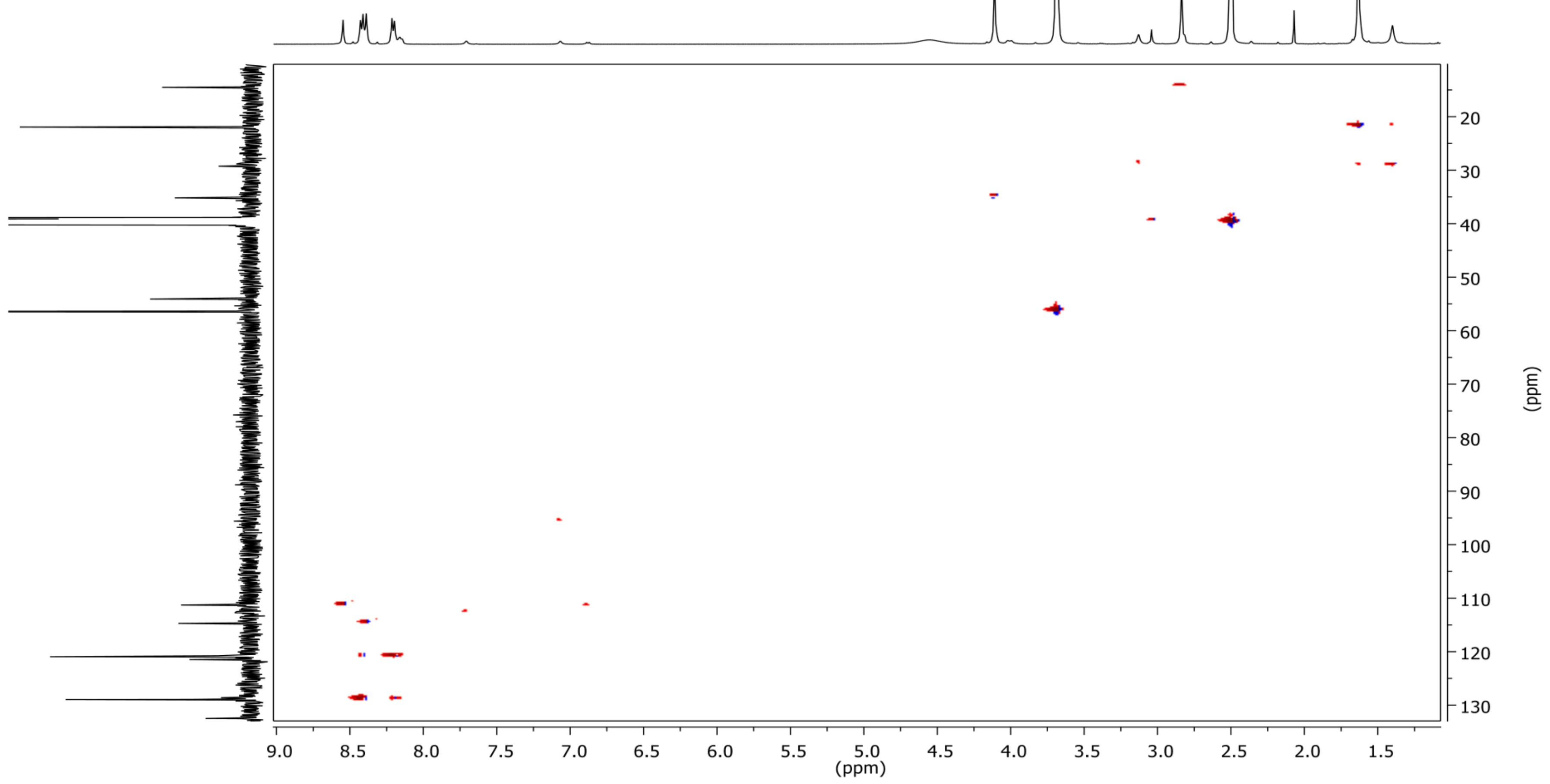

Figure S20. ¹H–¹³C gHSQC (500 MHz, d_6–DMSO): **12b**

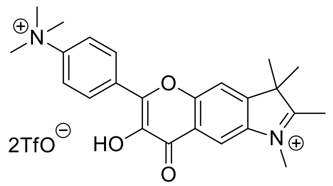

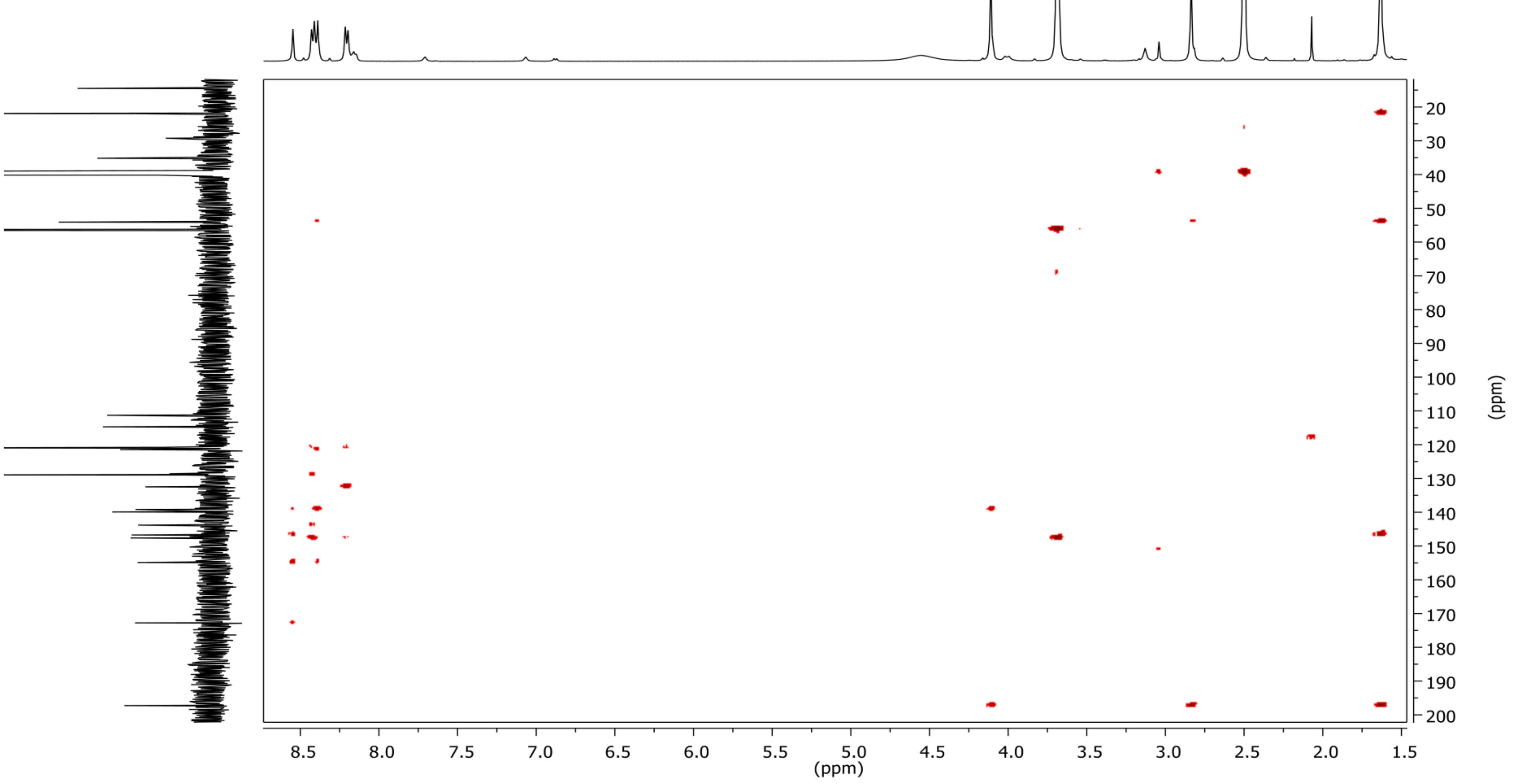

Figure S21. ^{1}H–^{13}C gHMBC (500 MHz, d_6–DMSO): **12b**

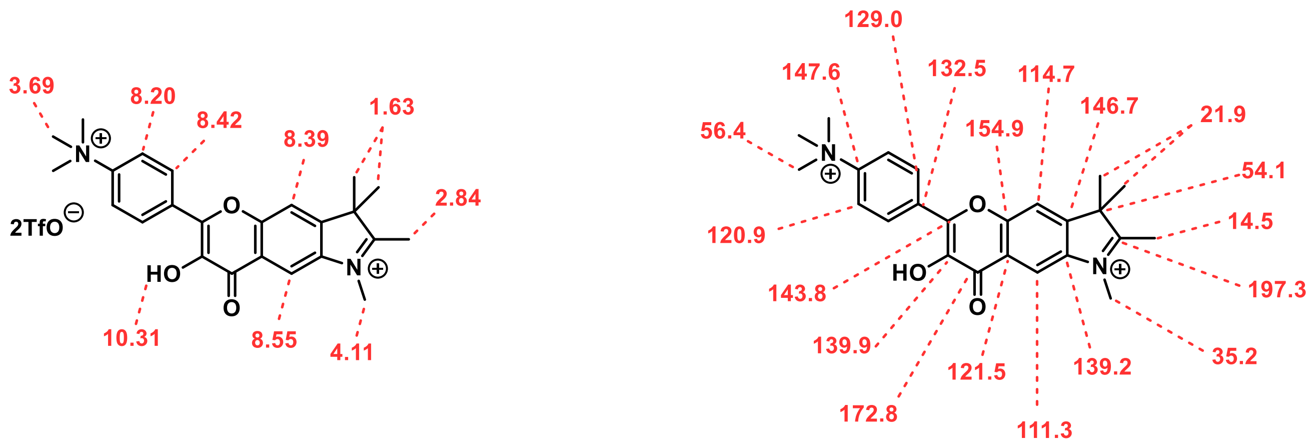

Figure S22. ^{1}H (left) and ^{13}C (right) NMR chemical shift assignments for **12b**.

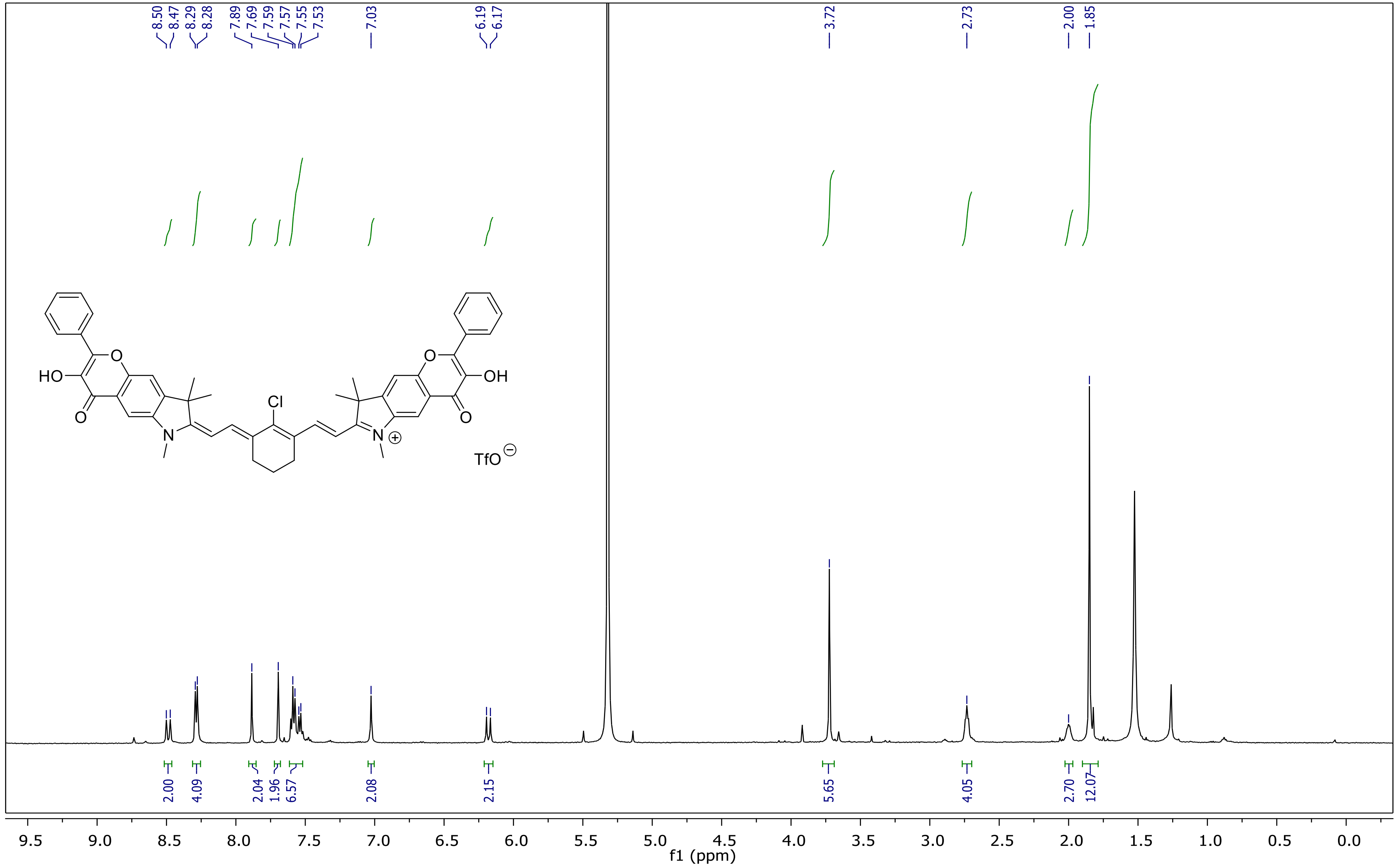

Figure S23. ^{1}H NMR (500 MHz, CD_2Cl_2): **5a**

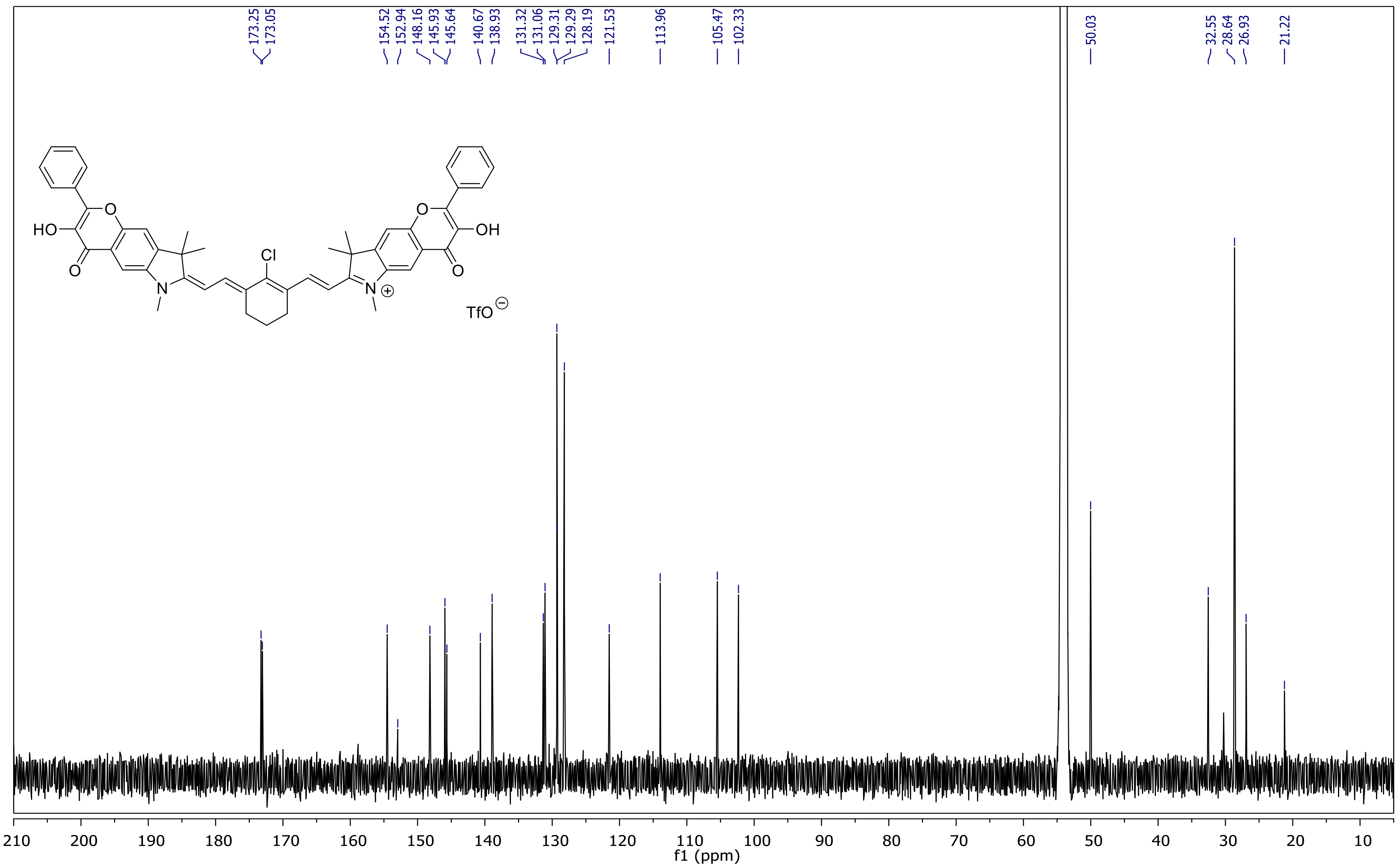

Figure S24. ^{13}C NMR (125 MHz, CD_2Cl_2): **5a**

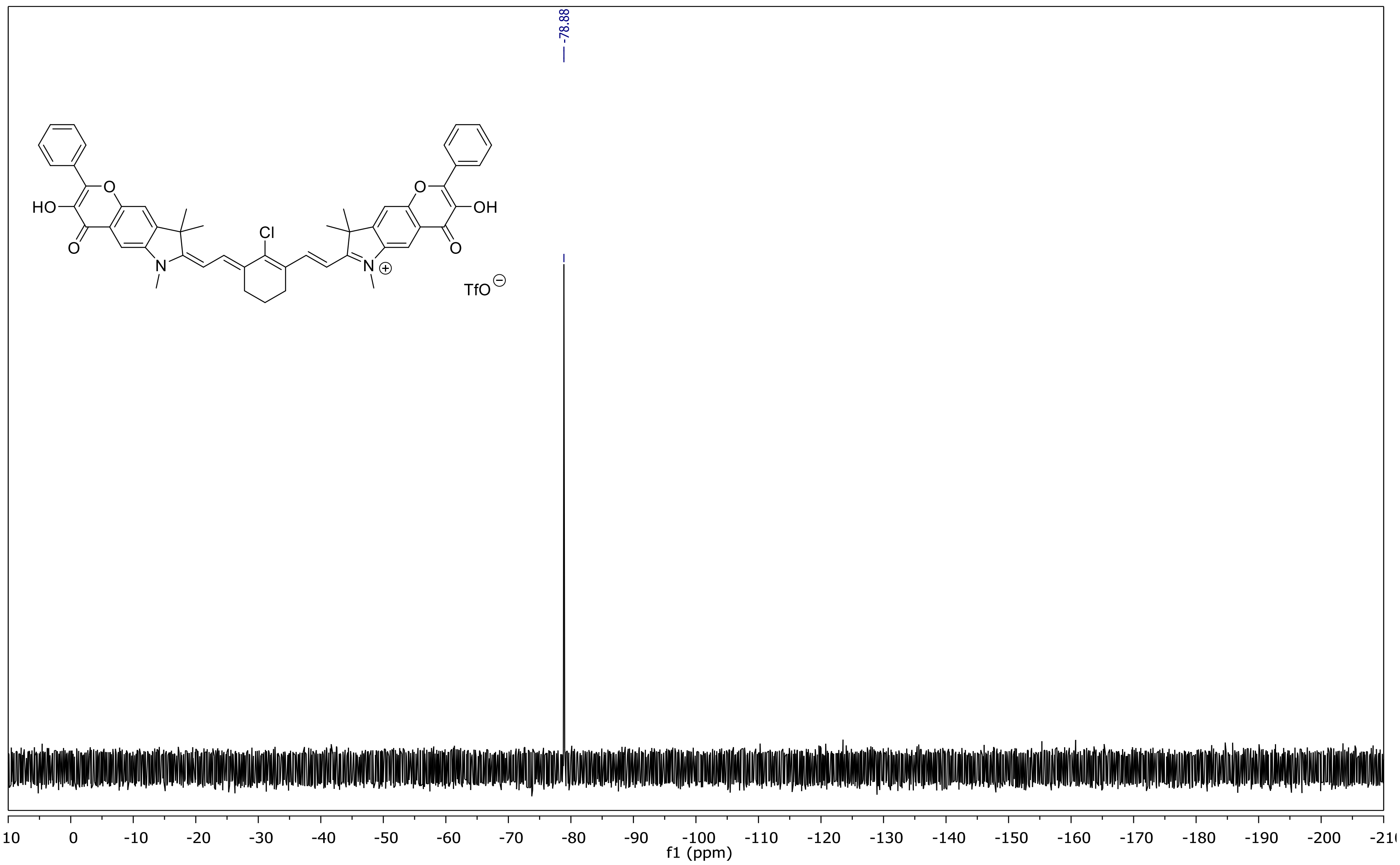

Figure S25. ^{19}F NMR (292 MHz, CD_2Cl_2): **5a**

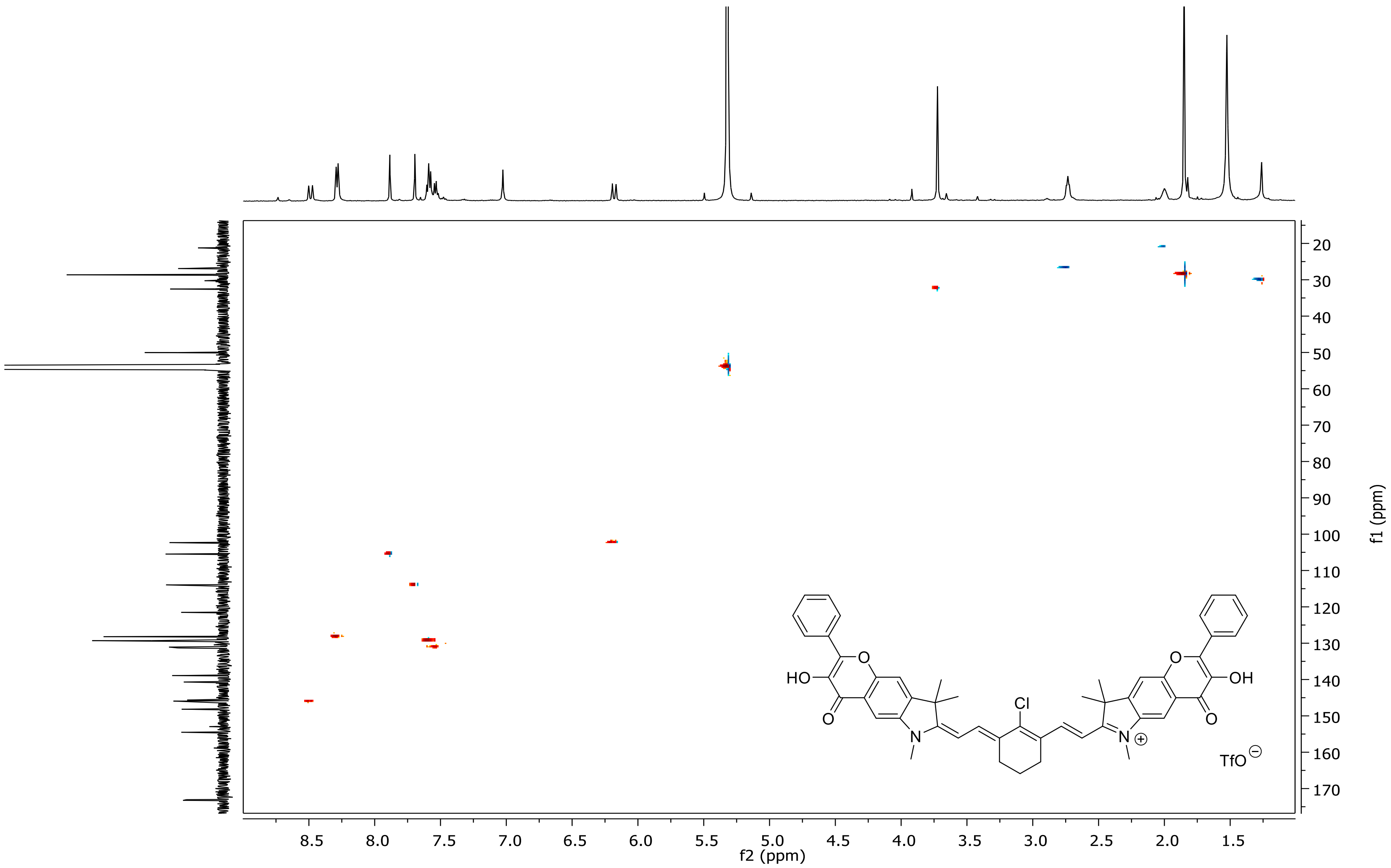

Figure S26. ^{1}H–^{13}C gHSQC (500 MHz, CD_2Cl_2): **5a**

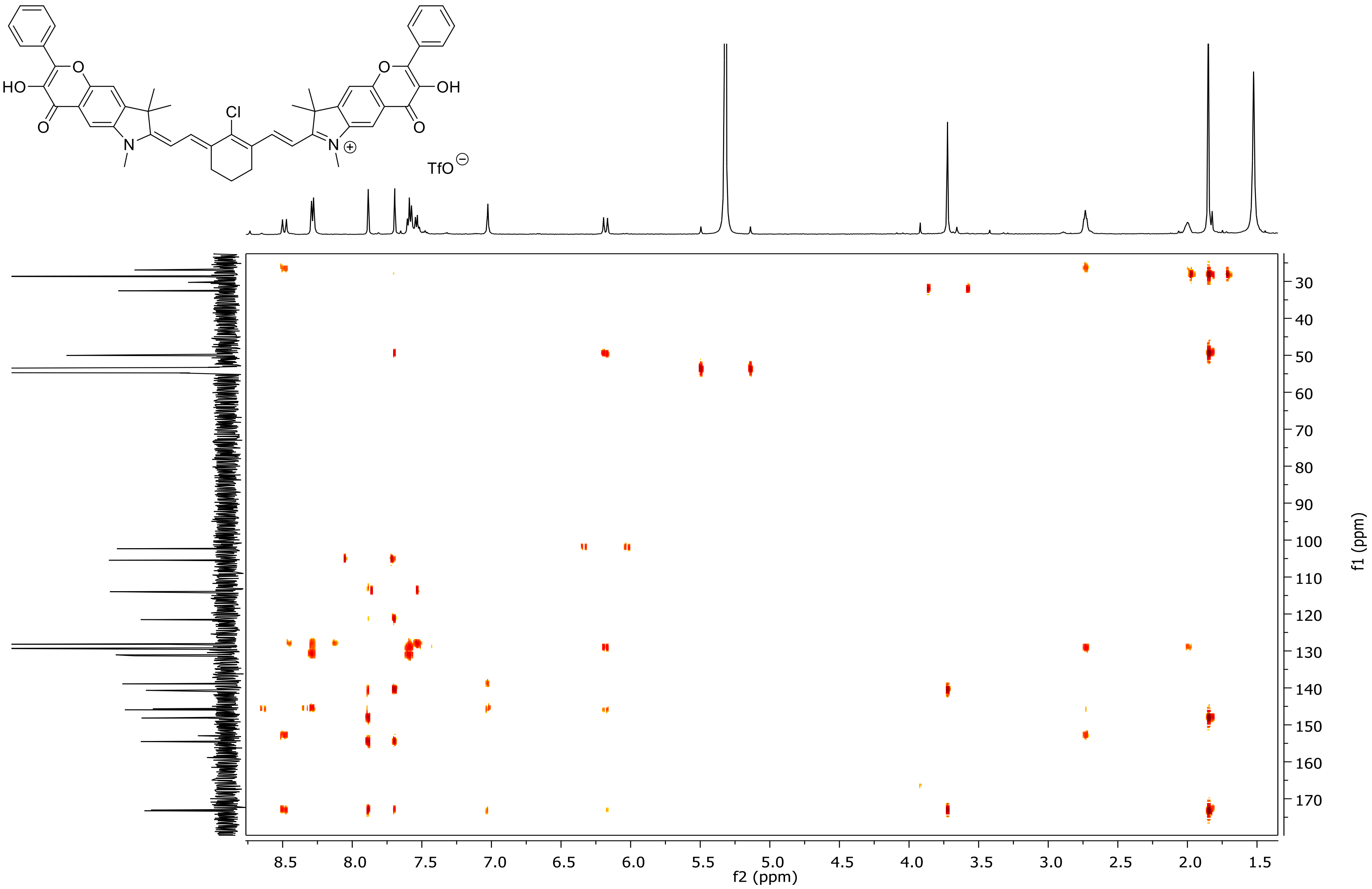

Figure S27. ^{1}H–^{13}C gHMBC (500 MHz, CD_2Cl_2): **5a**

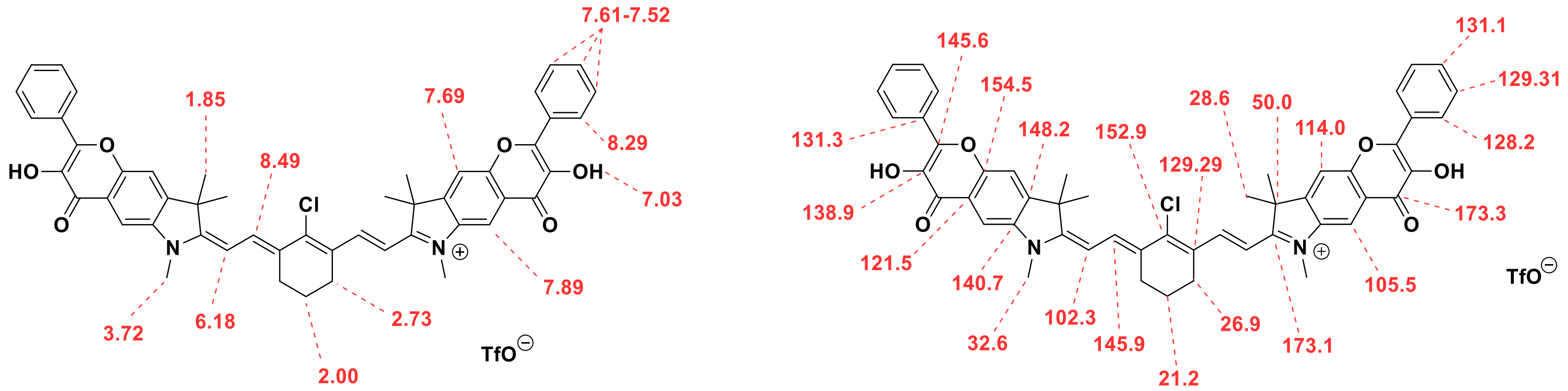

Figure S28. ^{1}H (left) and ^{13}C (right) NMR chemical shift assignments for **5a**

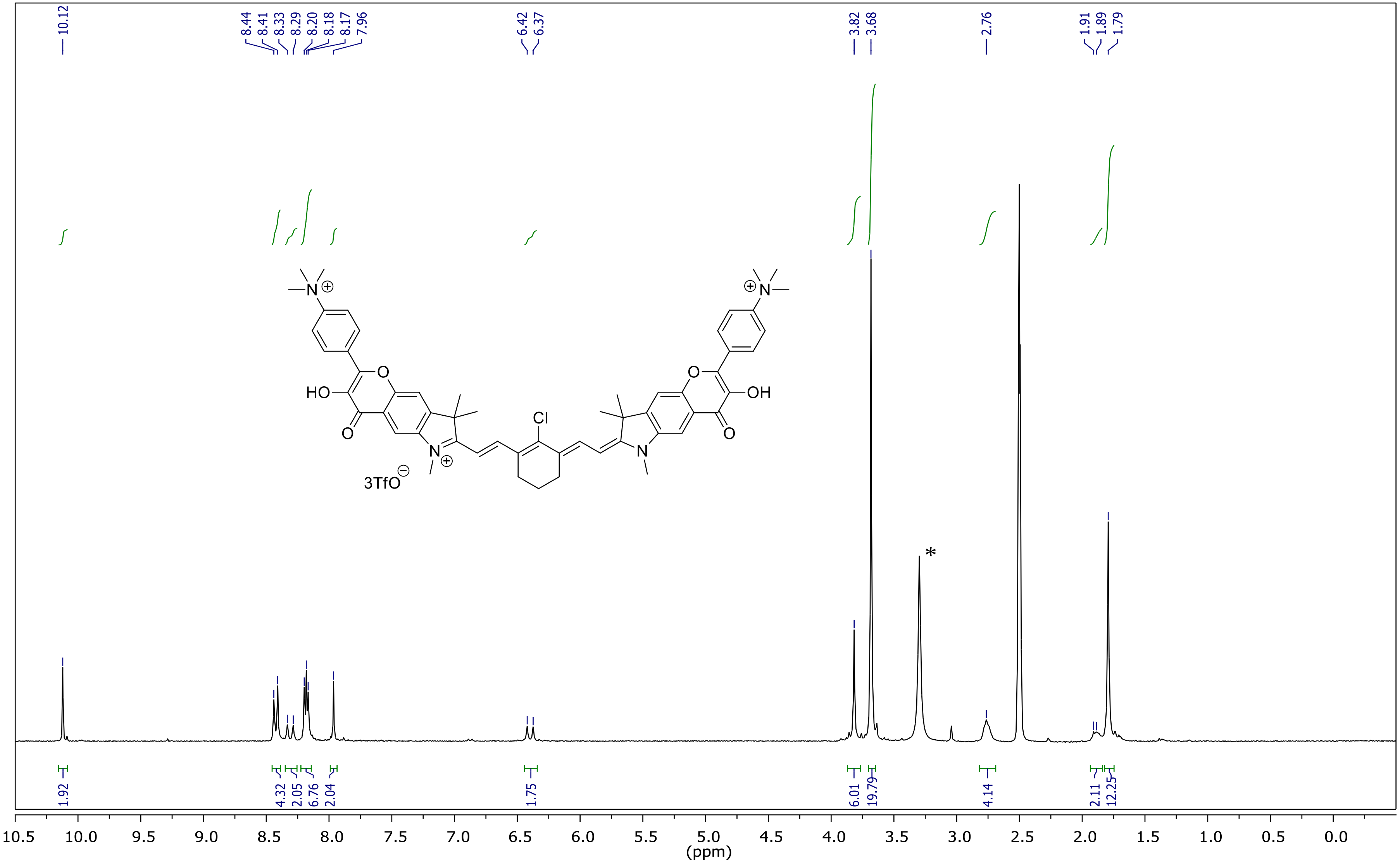

Figure S29. ^{1}H NMR (300 MHz, d_6–DMSO): **5b**. Asterisk denotes residual water from d_6–DMSO.

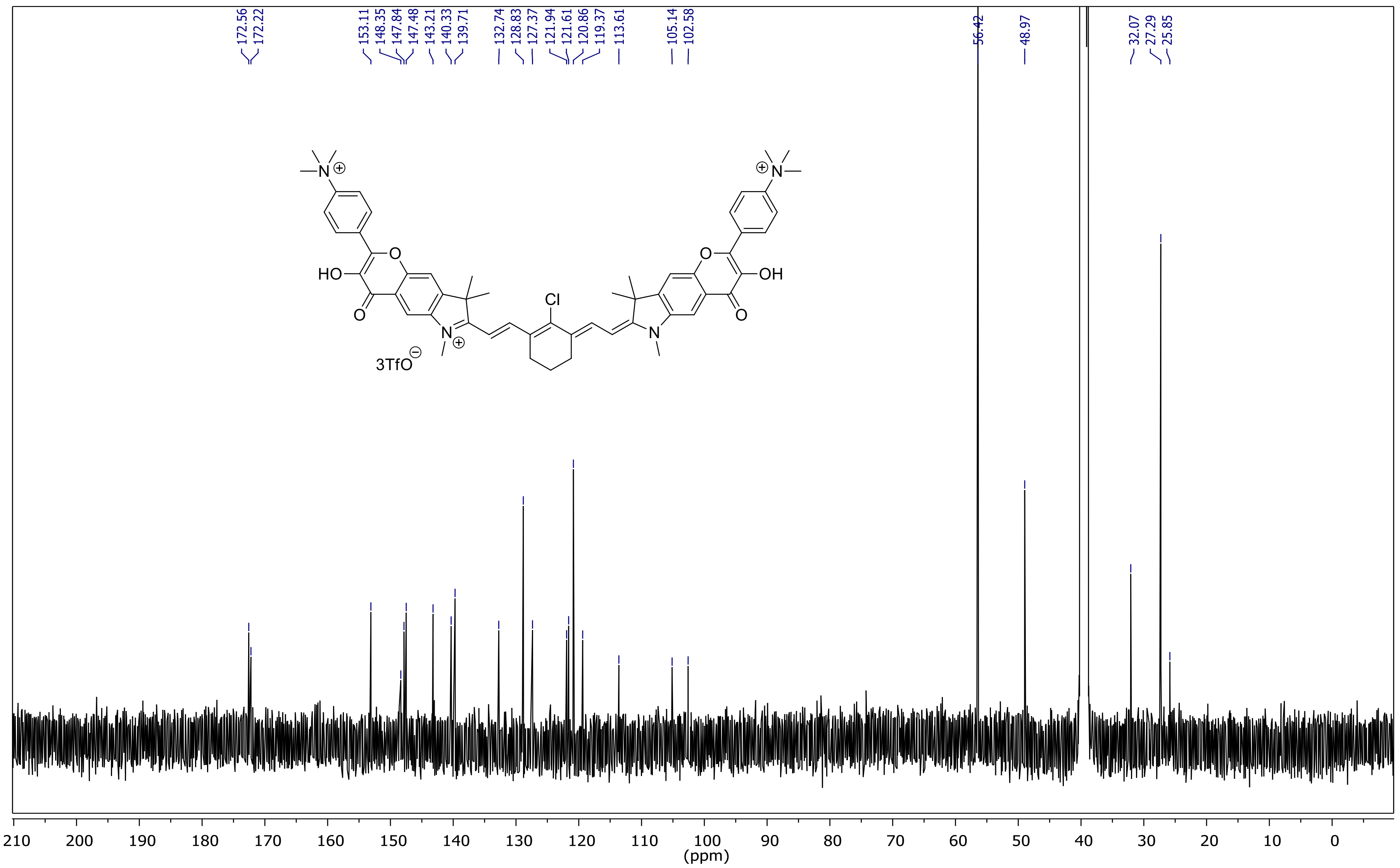

Figure S30. ^{13}C NMR (125 MHz, d_6–DMSO): **5b**

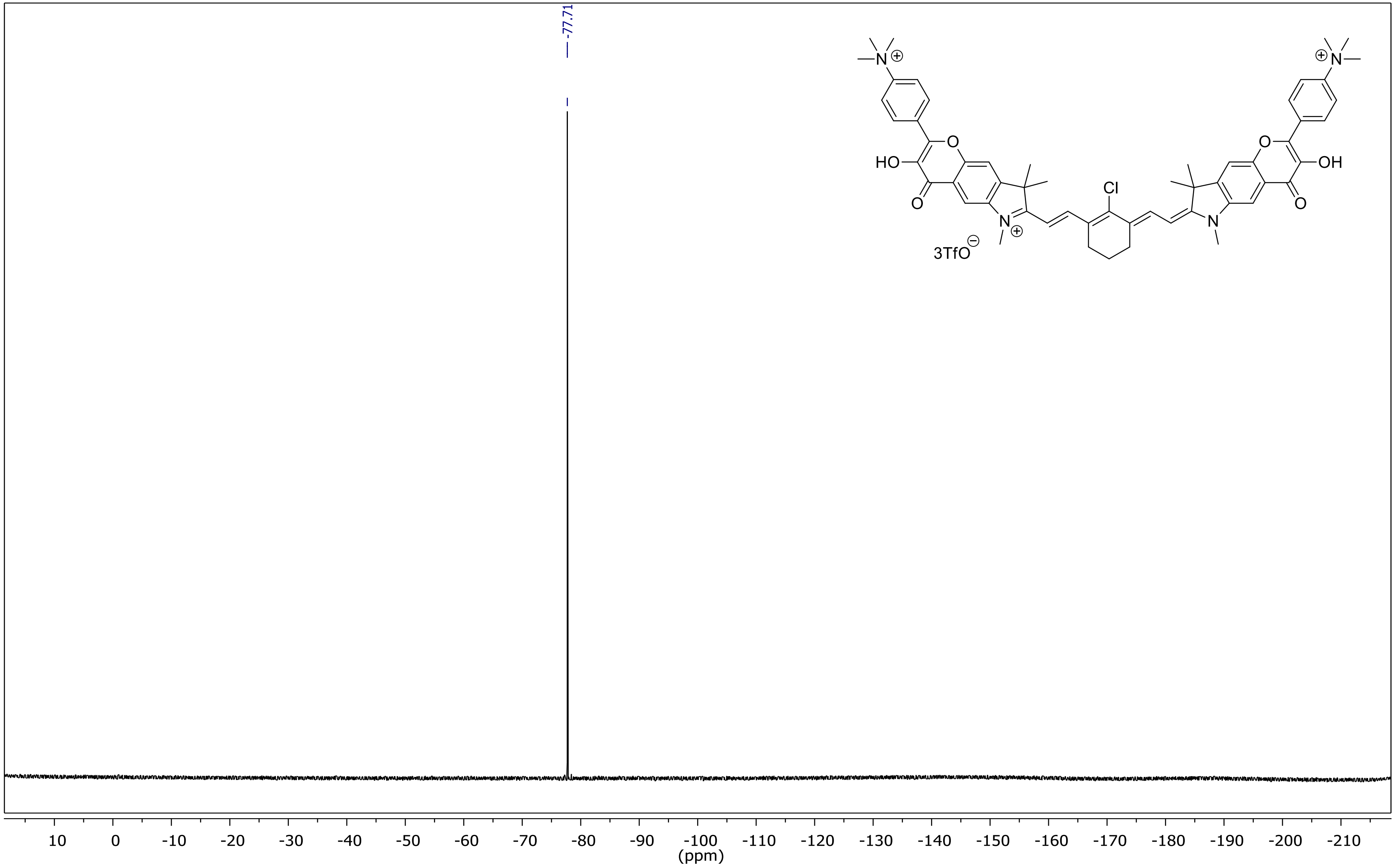

Figure S31. ^{19}F NMR (282 MHz, d_6–DMSO): **5b**

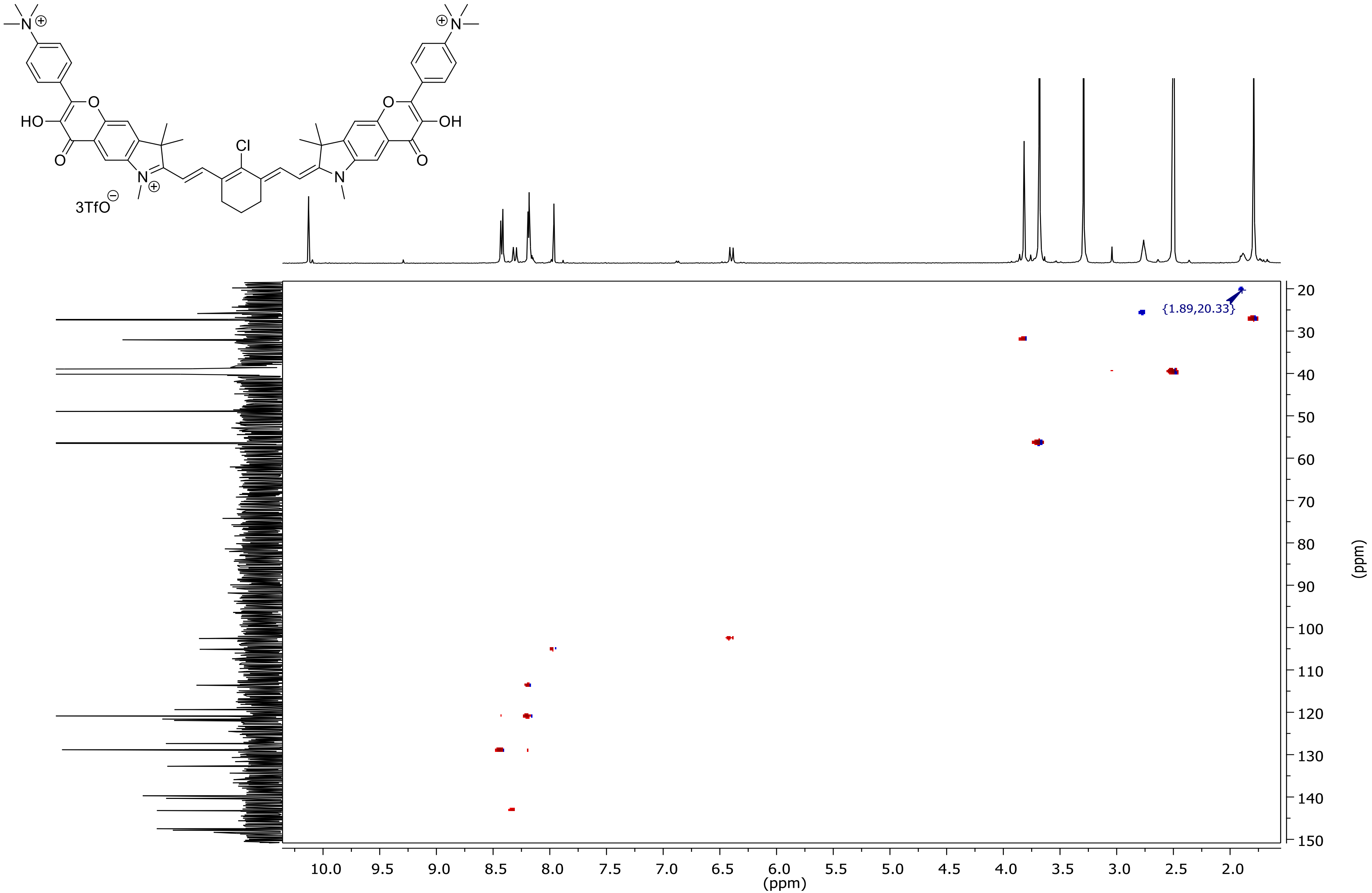

Figure S32. ^{1}H–^{13}C gHSQC (500 MHz, d_6–DMSO): **5b**

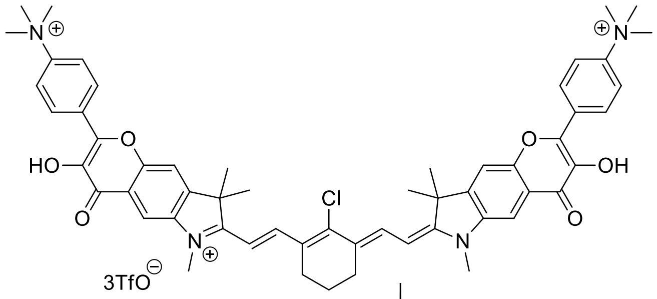

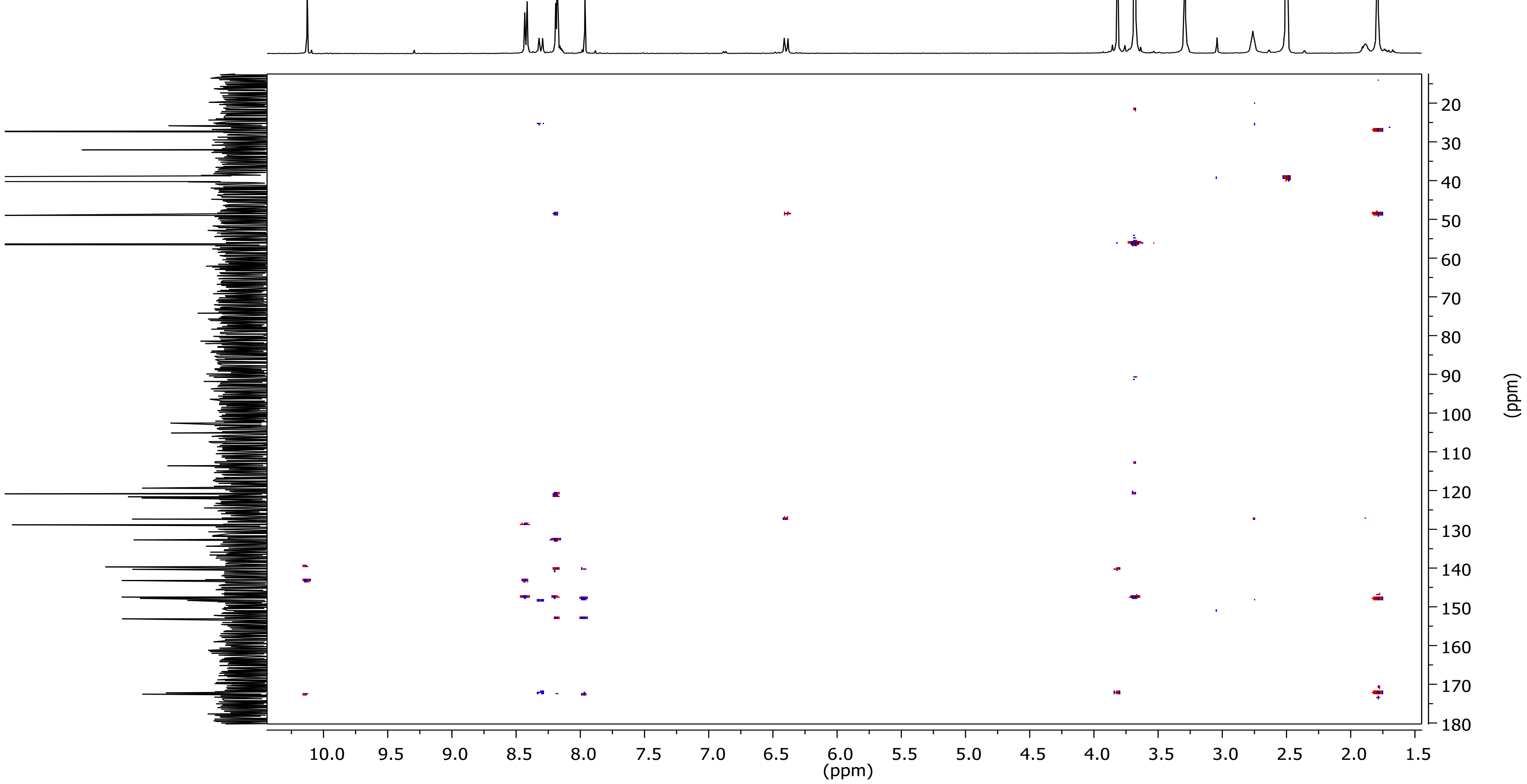

Figure S33. ^{1}H–^{13}C gHMBC (500 MHz, d_6–DMSO): **5b**

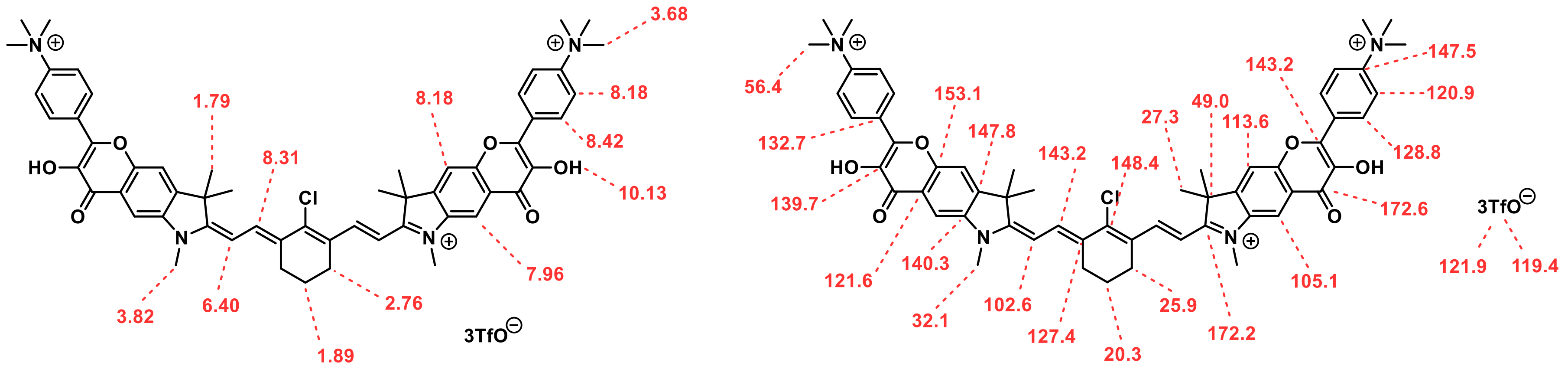

Figure S34. ^{1}H (left) and ^{13}C (right) NMR chemical shift assignments for **5b**.

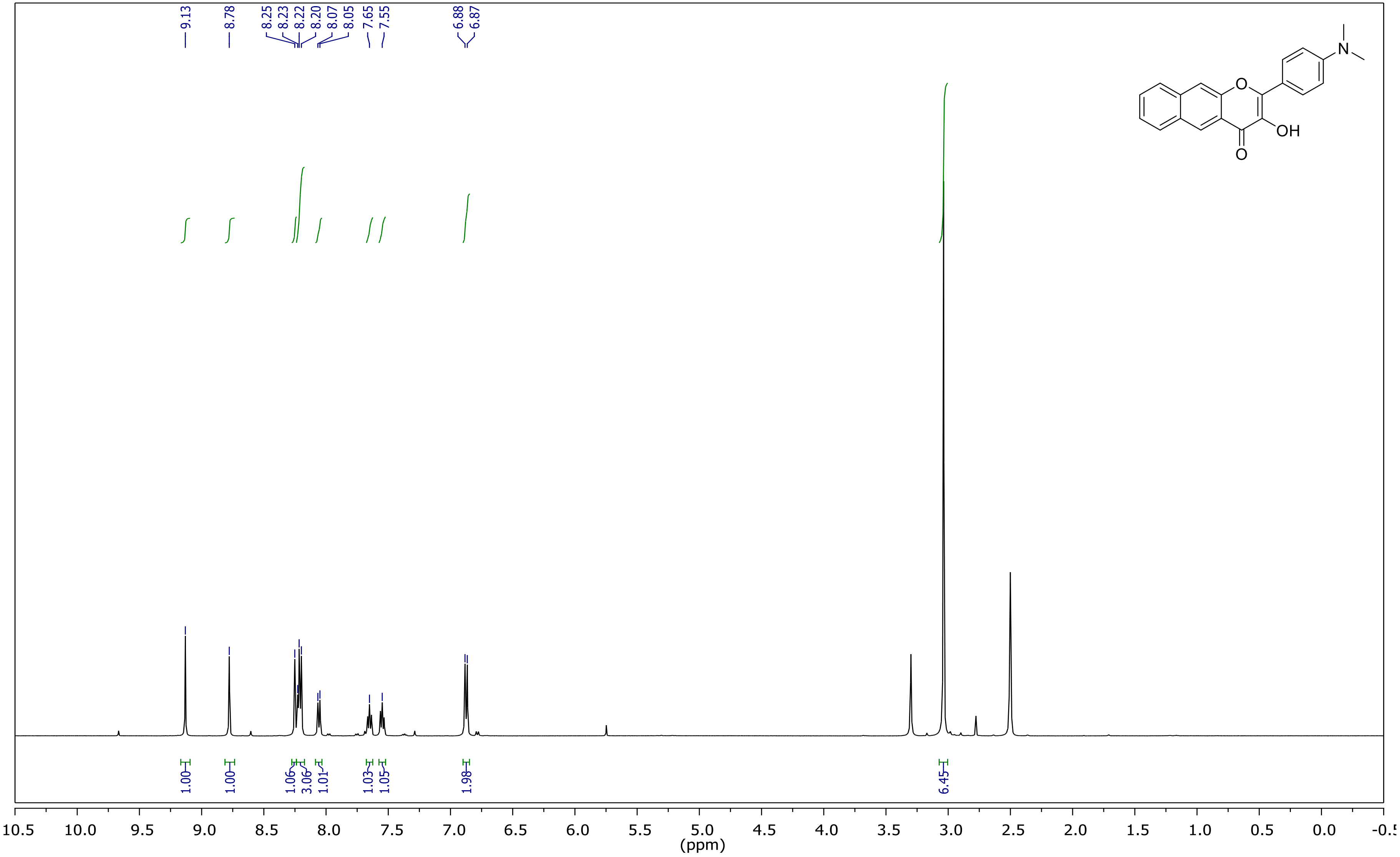

Figure S35. ^{1}H NMR (500 MHz, d_6-DMSO): **17**

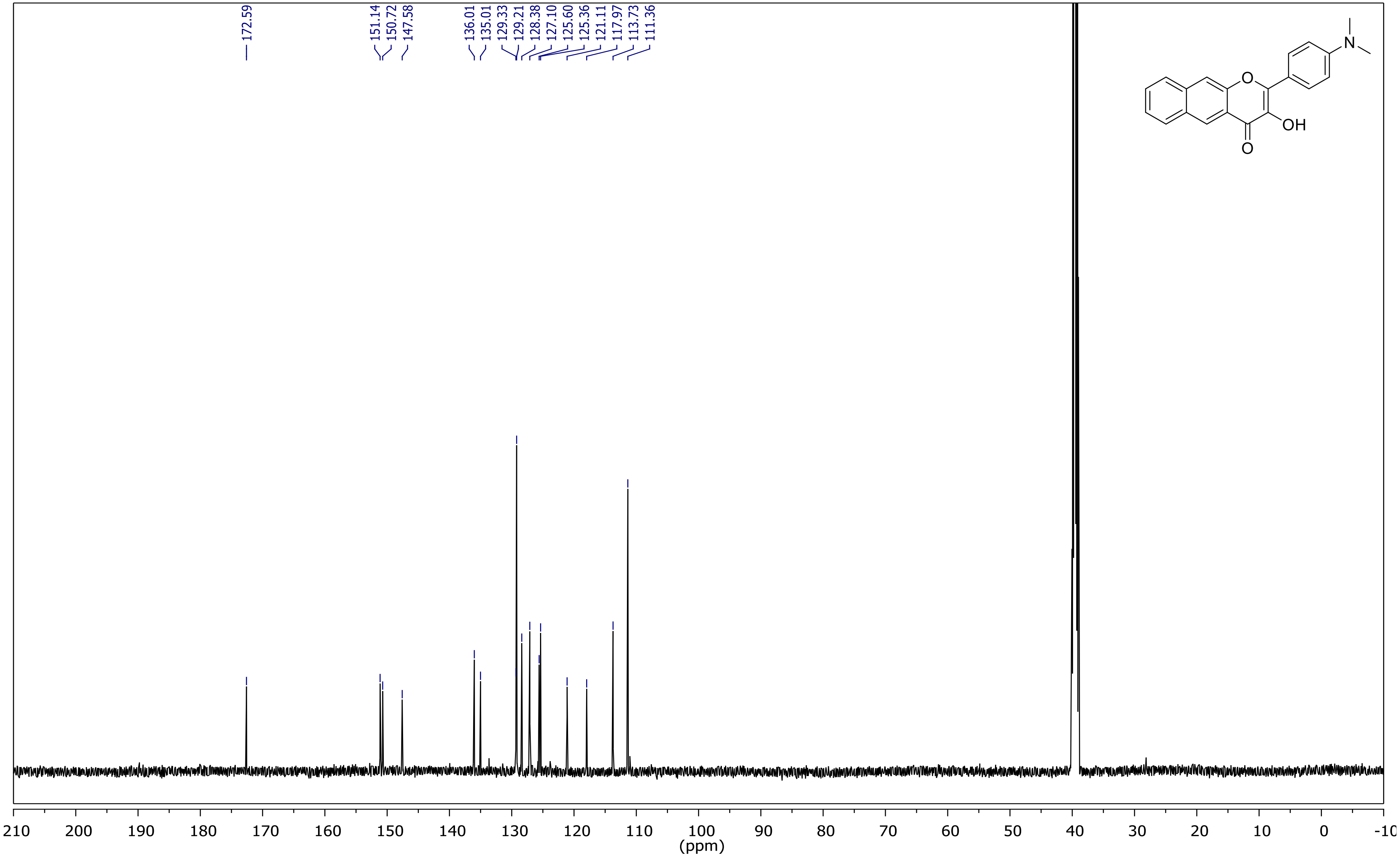

Figure S36. ^{13}C NMR (125 MHz, d_6-DMSO): **17**

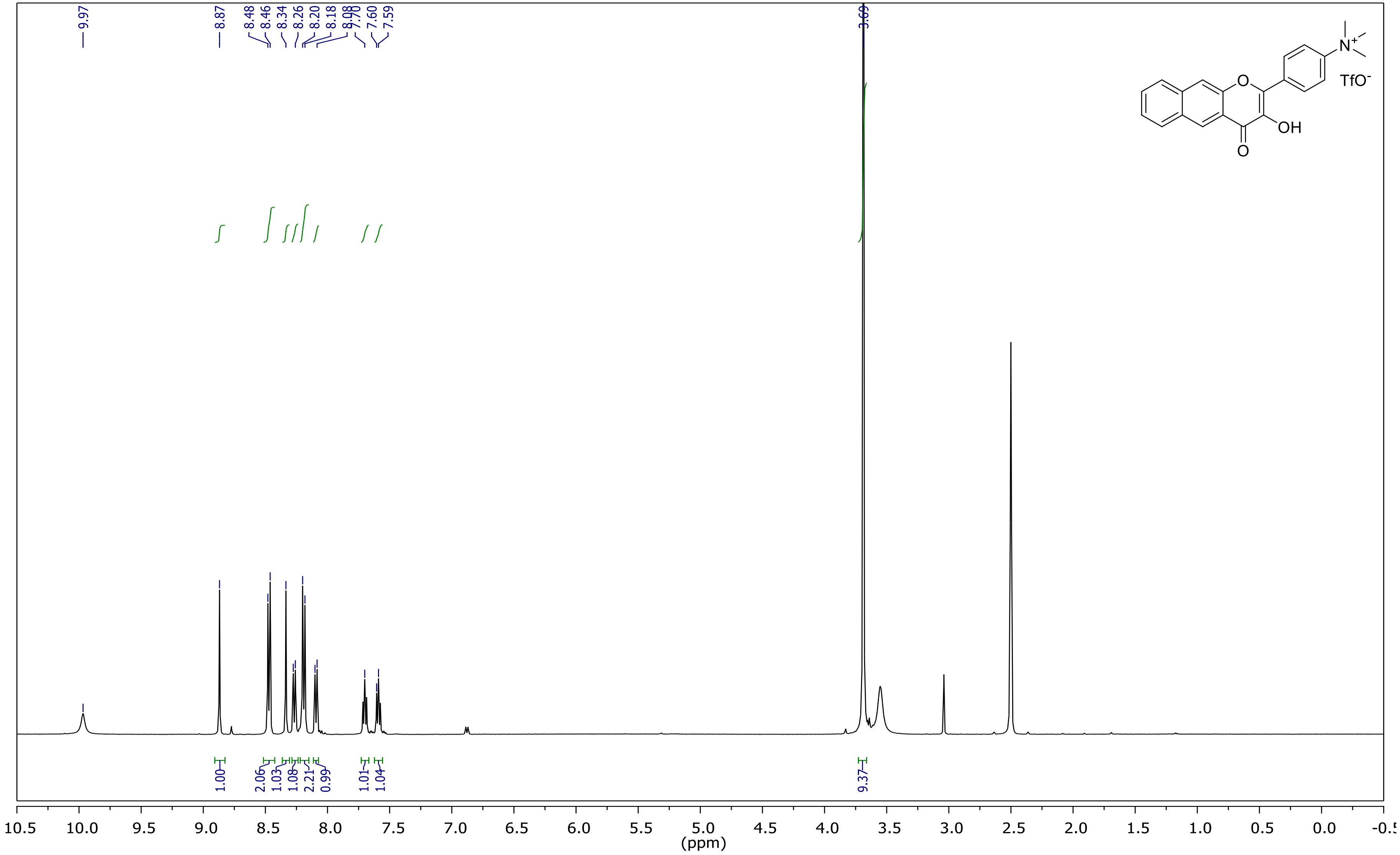

Figure S37. ^{1}H NMR (500 MHz, d_6-DMSO): **14**

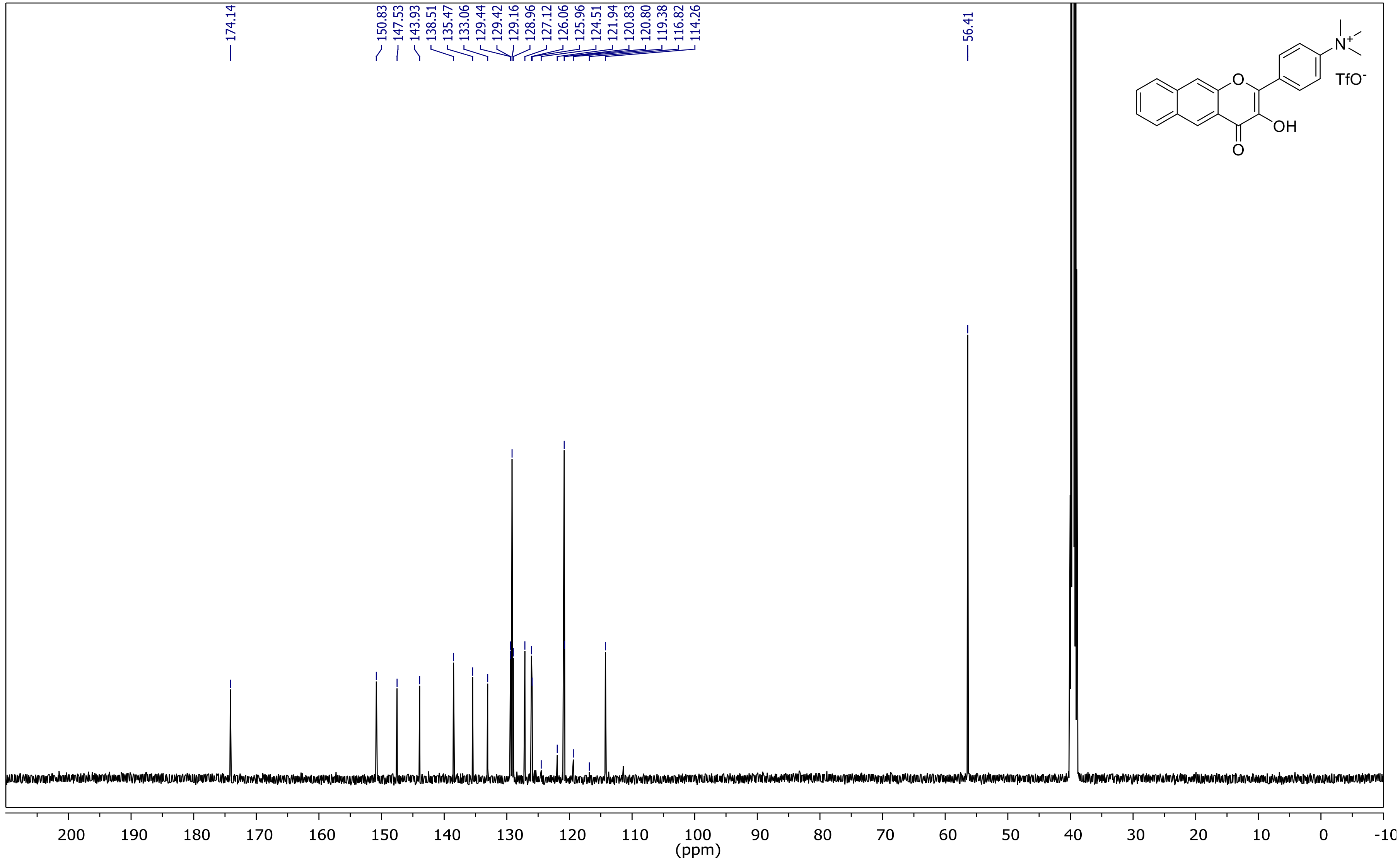

Figure S38. ^{13}C NMR (125 MHz, d_6-DMSO): **14**

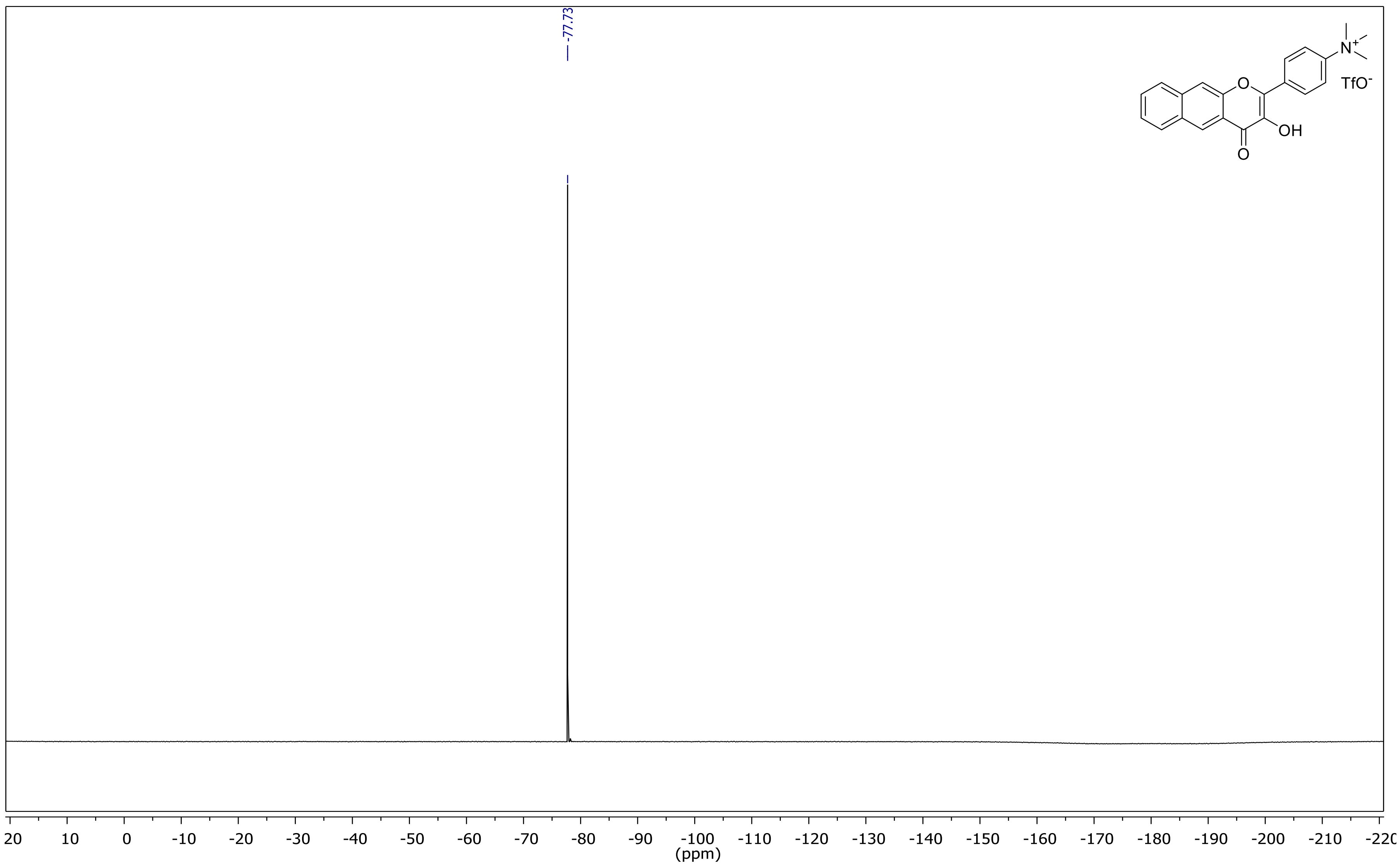

Figure S39. ^{19}F NMR (476 MHz, d_6-DMSO): **14**

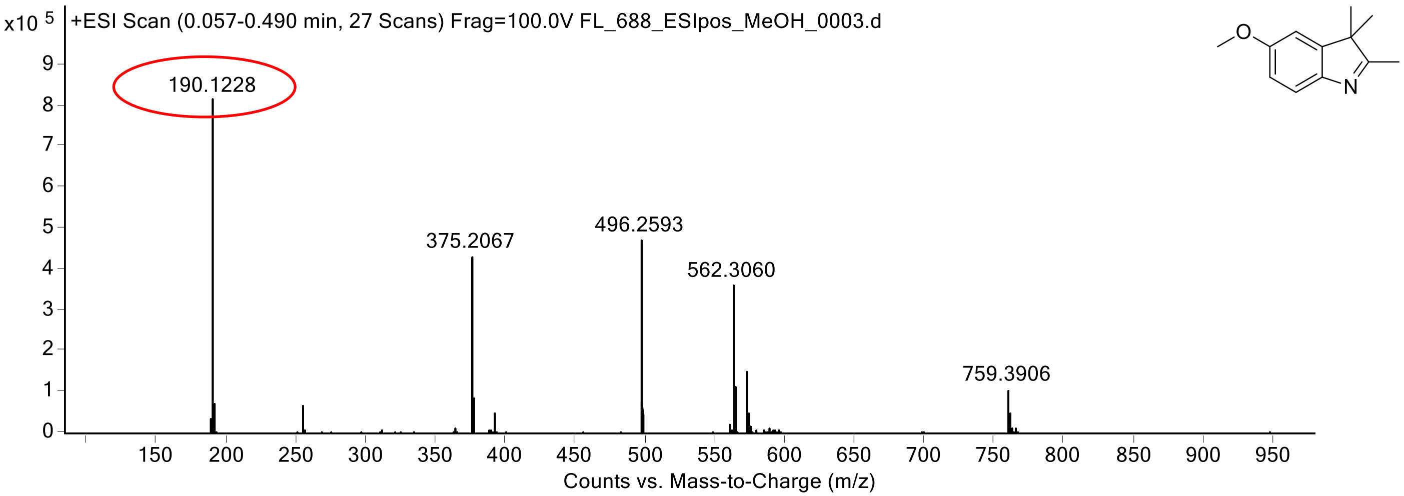

Figure S40. HRMS (ESI+): **7** (calcd. for $C_{12}H_{16}NO^+$ [M + H$^+$] 190.1226, found 190.1228.)

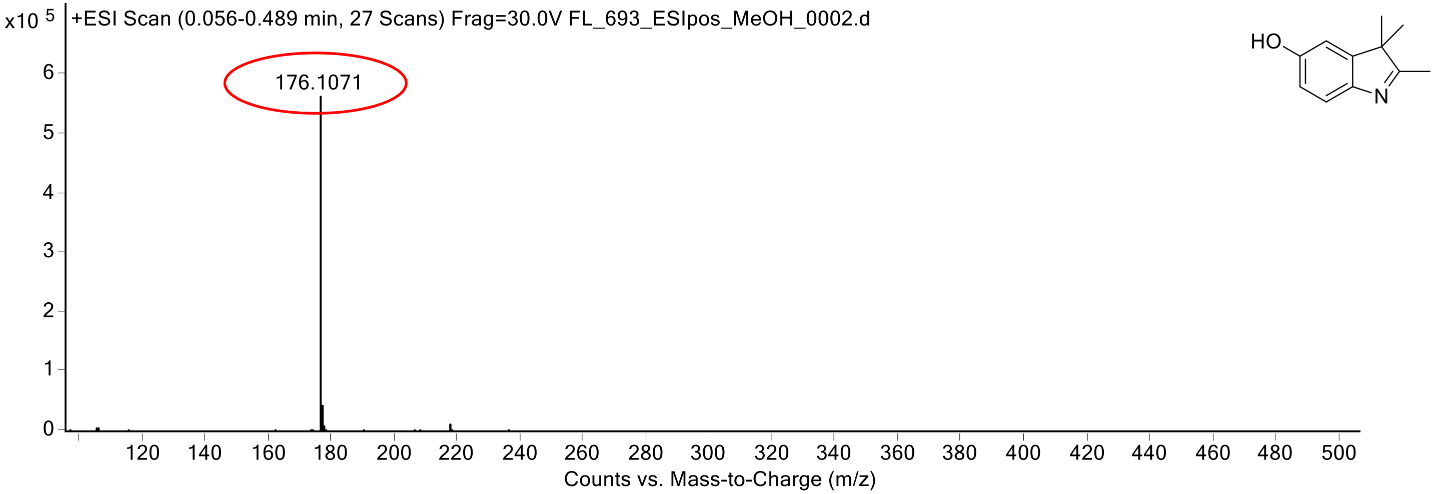

Figure S41. HRMS (ESI+): **8** (calcd. for $C_{11}H_{14}NO^+$ [M + H$^+$] 176.1070, found 176.1071)

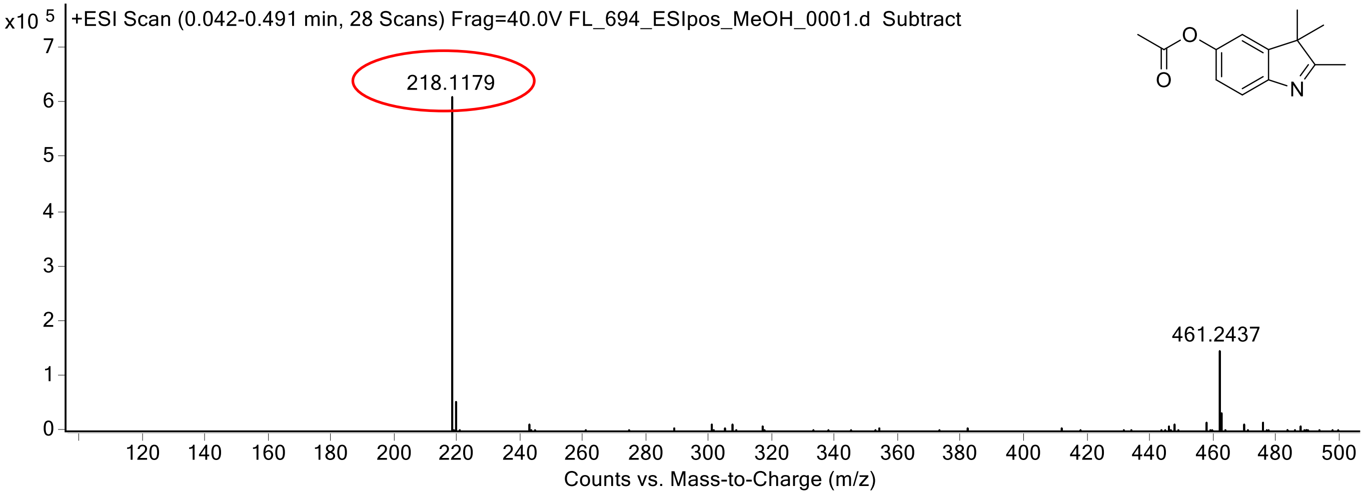

Figure S42. HRMS (ESI+): **9** (calcd. for $C_{13}H_{16}NO_2^+$ [M + H$^+$] 218.1176, found 218.1179)

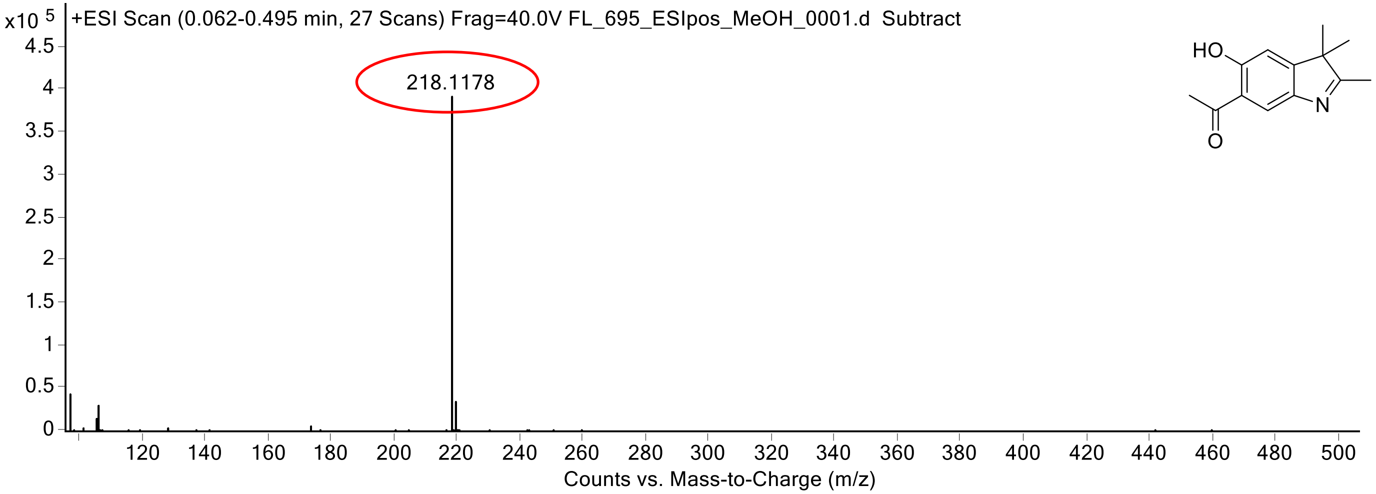

Figure S43. HRMS (ESI+): **10 (**calcd. for $C_{13}H_{16}NO_2^+$ [M + H$^+$] 218.1176, found 218.1178)

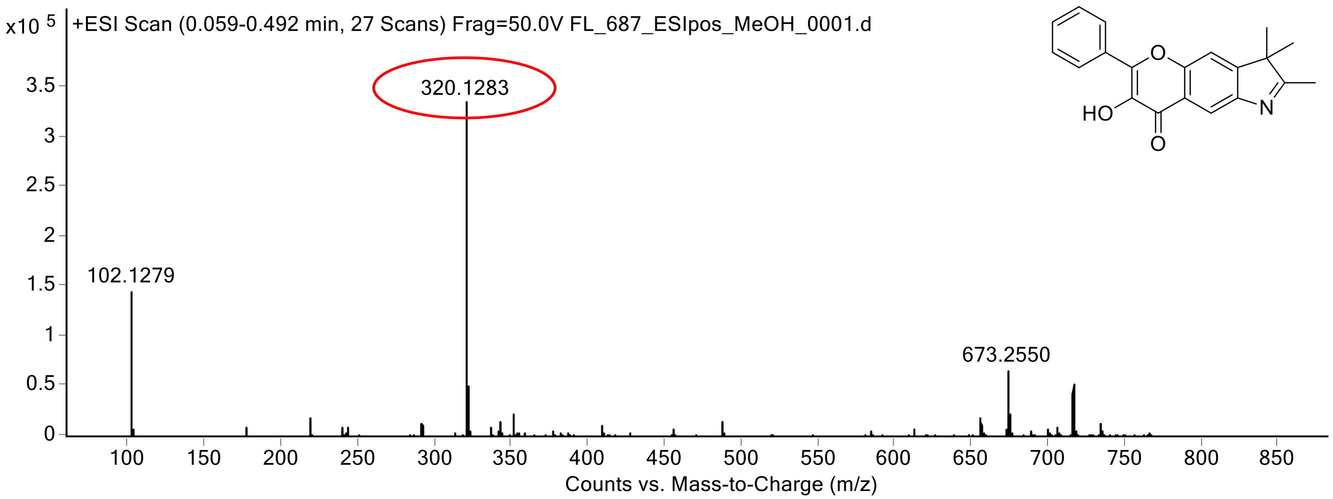

Figure S44. HRMS (ESI+): **11a (**calcd. for $C_{20}H_{18}NO_3^+$ [M + H$^+$] 320.1281, found 320.1283)

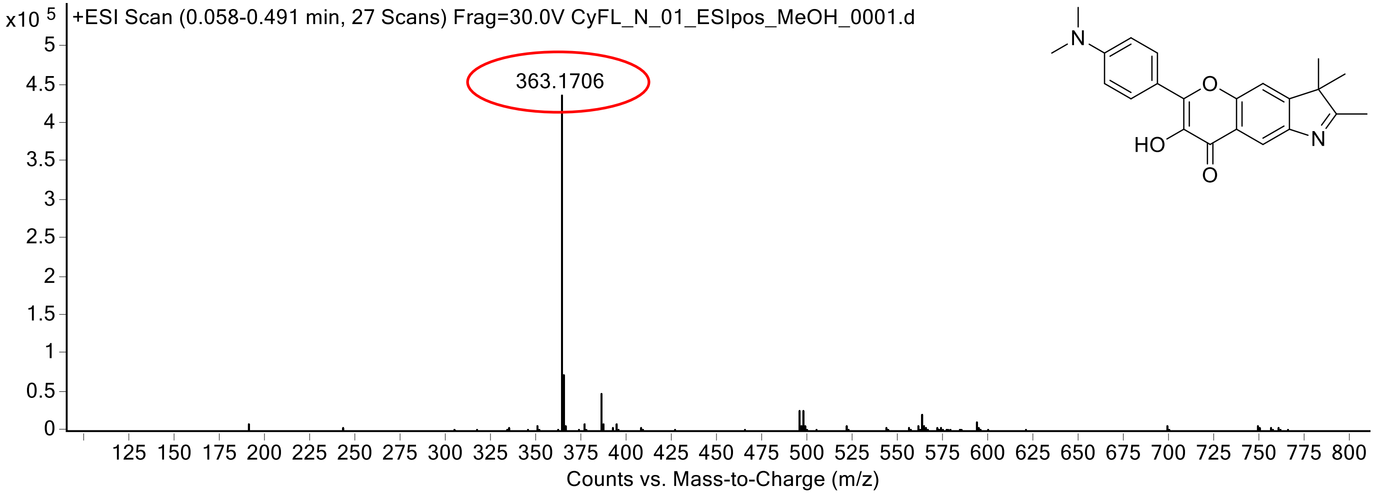

Figure S45. HRMS (ESI+): **11b (**calcd. for $C_{22}H_{23}N_2O_3^+$ [M + H$^+$] 363.1703, found 363.1706)

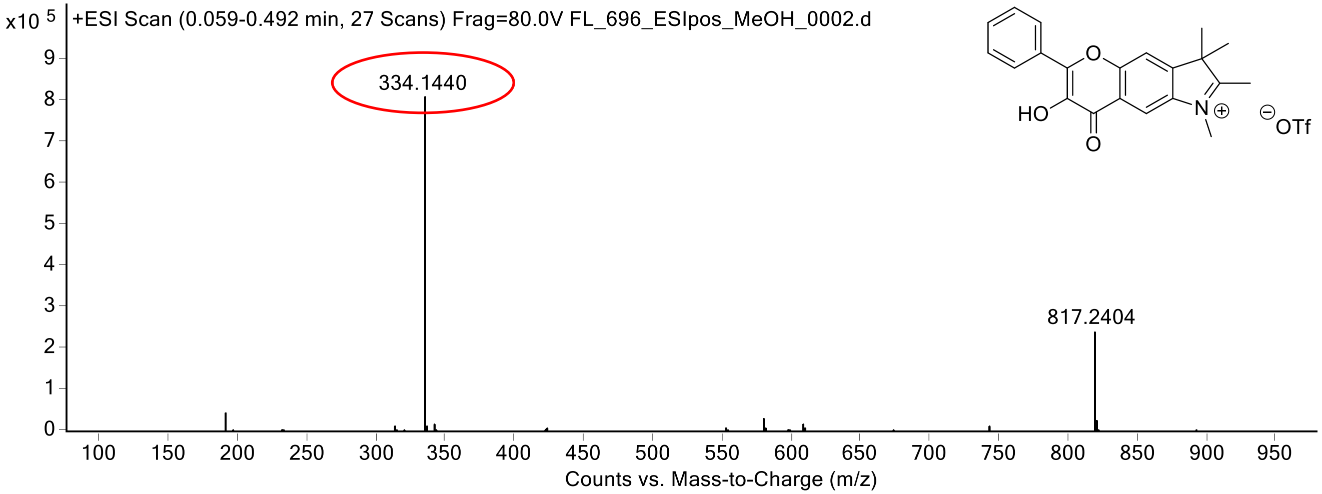

Figure S46. HRMS (ESI+): **12a (**calcd. for $C_{21}H_{20}NO_3^+$ [M^+] 334.1438, found 334.1440)

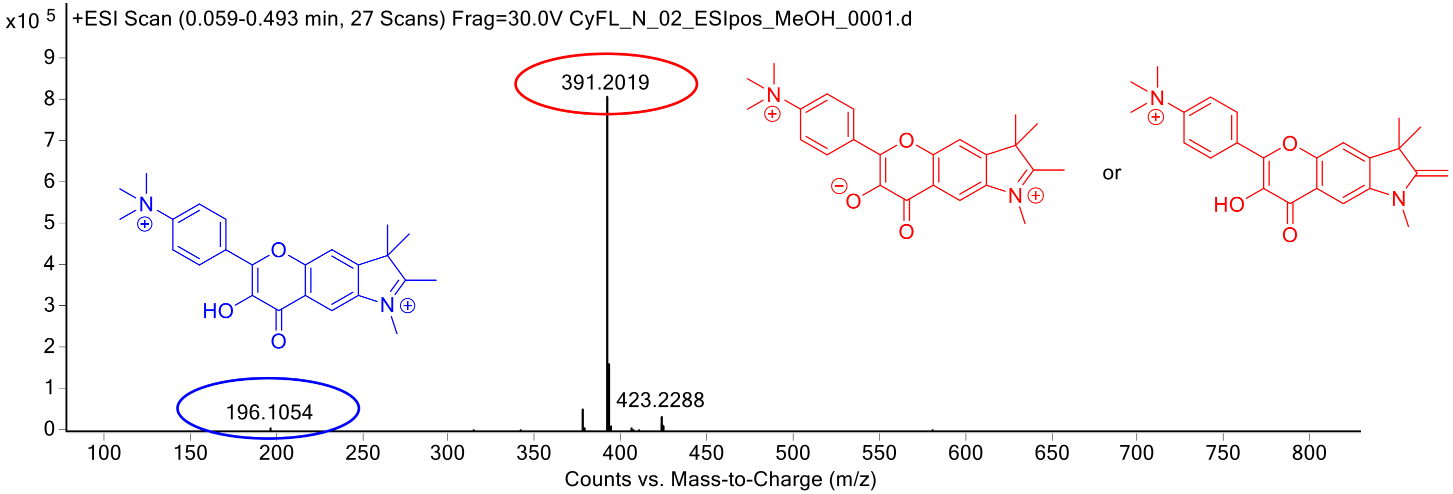

Figure S47. HRMS (ESI+): **12b (**calcd. for $C_{24}H_{27}N_2O_3^+$ [M^+] 391.2016, found 391.2019)

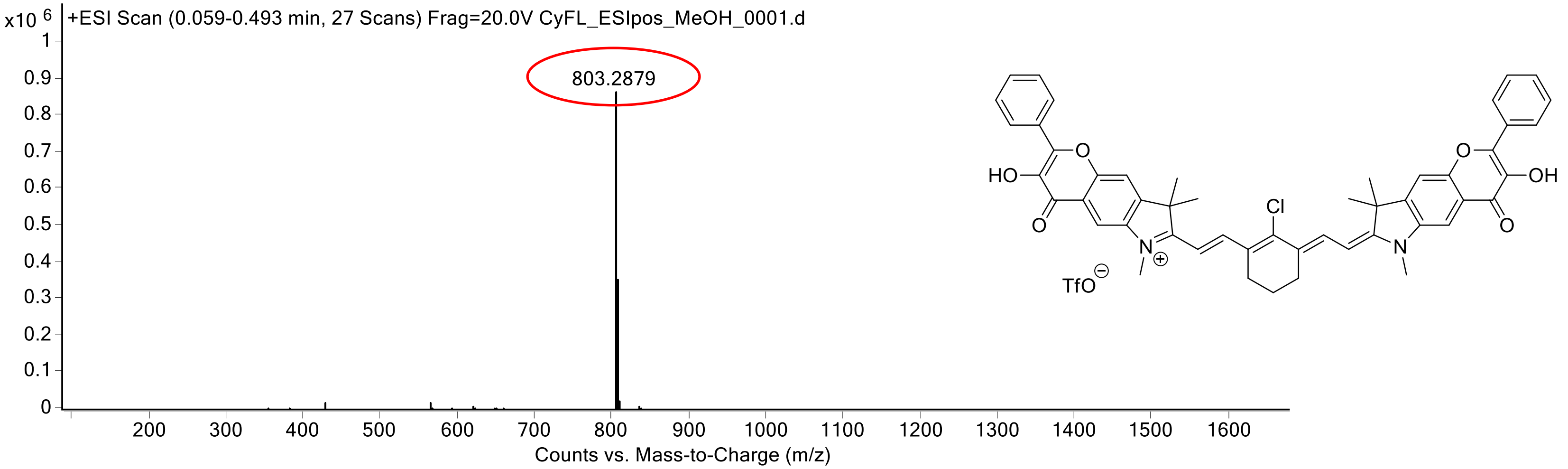

Figure S48. HRMS (ESI+): **5a** (calcd. for $C_{50}H_{44}ClN_2O_6^+$ [M^+] 803.2882, found 803.2879)

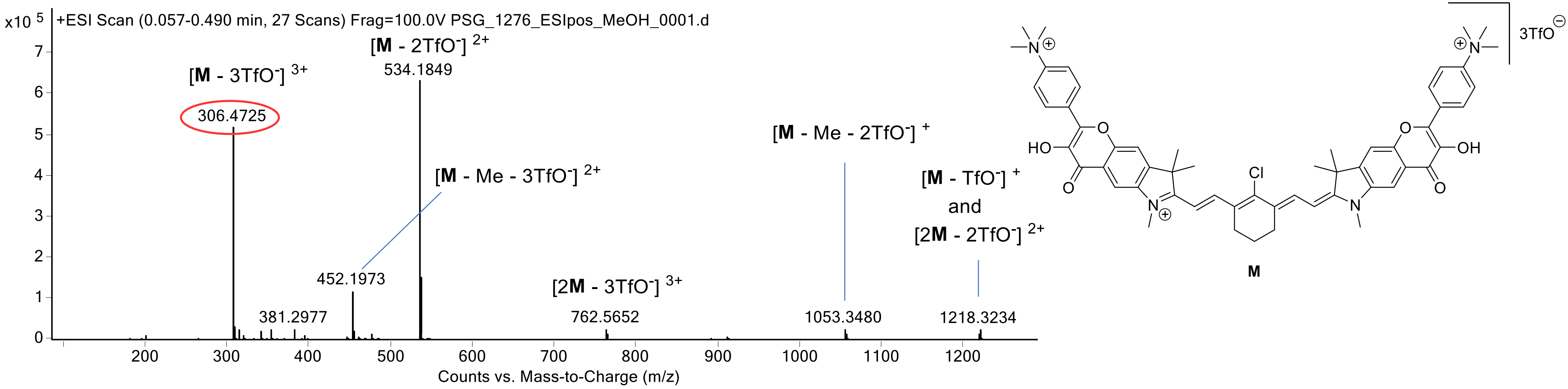

Figure S49. HRMS (ESI+): **5b** (calcd. for $C_{56}H_{60}ClN_4O_6^{3+}$ [M^{3+}] 306.4728, found 306.4729)

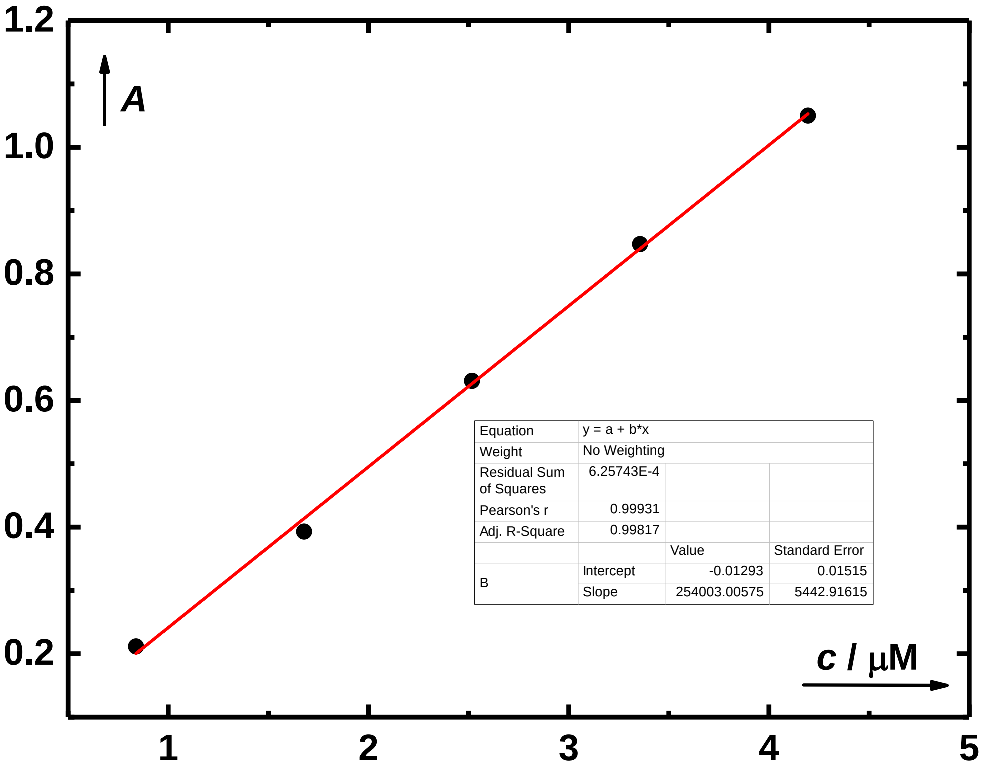

Figure S50. Dependence of the absorbance at the absorption maximum (λ_{max} = 791 nm) on the concentration of **5a** in methanol.

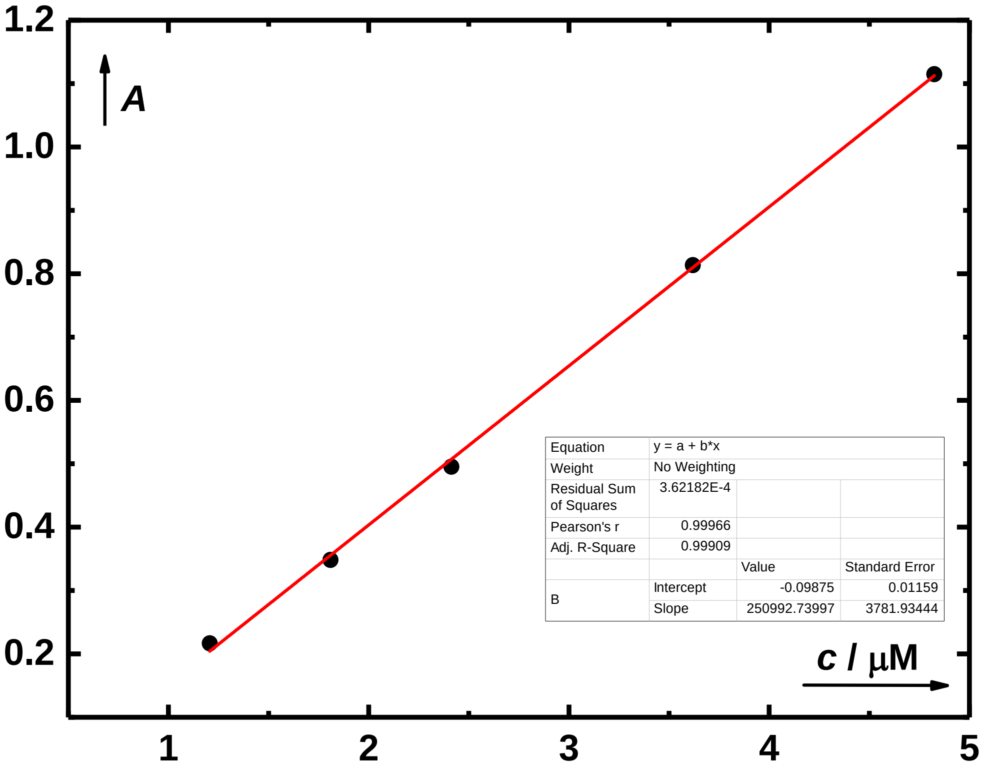

Figure S51. Dependence of the absorbance at the absorption maximum (λ_{max} = 793 nm) on the concentration of **5b** in methanol.

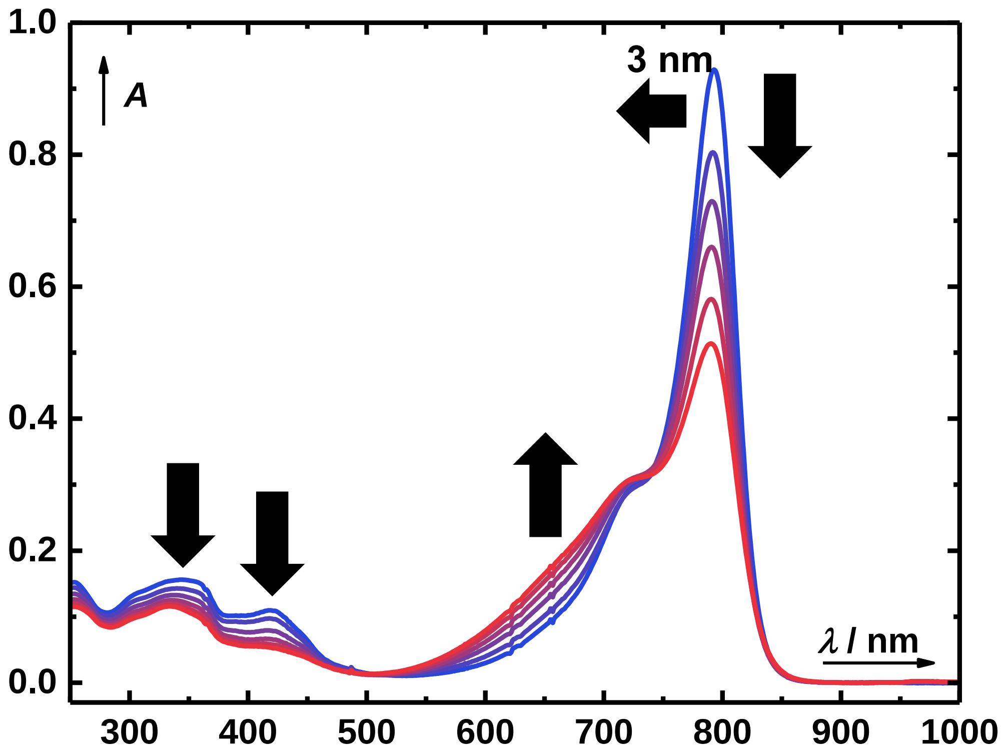

Figure S52. Irradiation of **5b** (c ~ 3.8 × 10^{-6} M) at 820 nm in aerated methanol followed by UV-vis spectroscopy in 10-min intervals (blue to red). The first 50 minutes are shown.

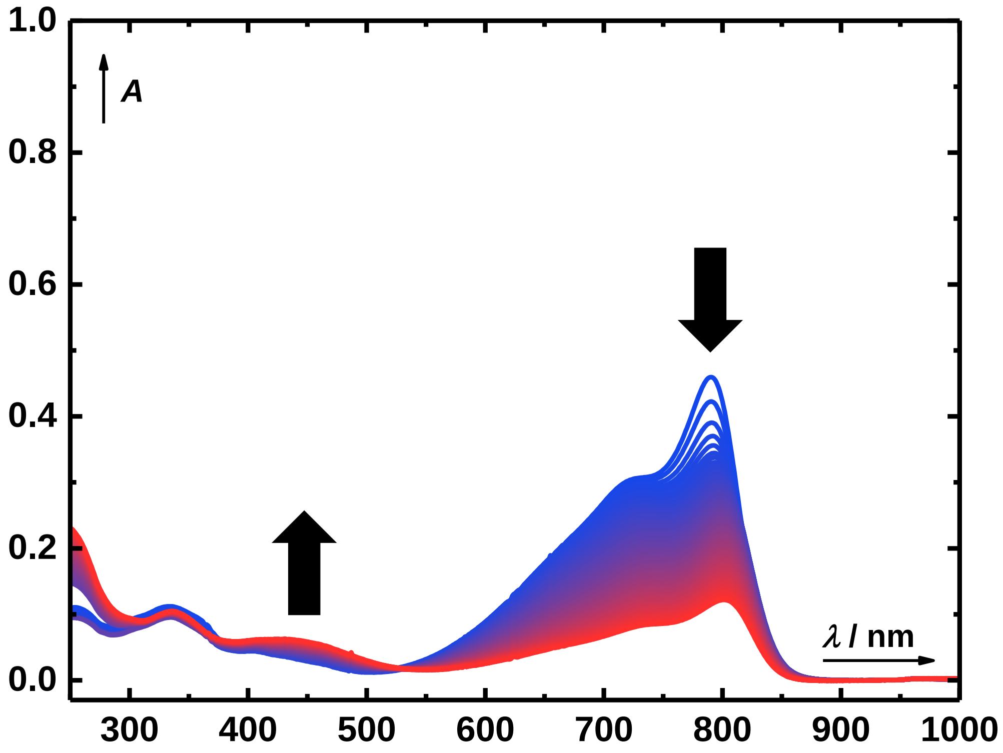

Figure S53. Irradiation of **5b** (c ~ 3.8 × 10^{-6} M) at 820 nm in aerated methanol followed by UV-vis spectroscopy in 10-min intervals (blue to red) corresponding to photobleaching of a cyanine scaffold. The time interval from t = 50 min to 900 min (see also Figure S7).

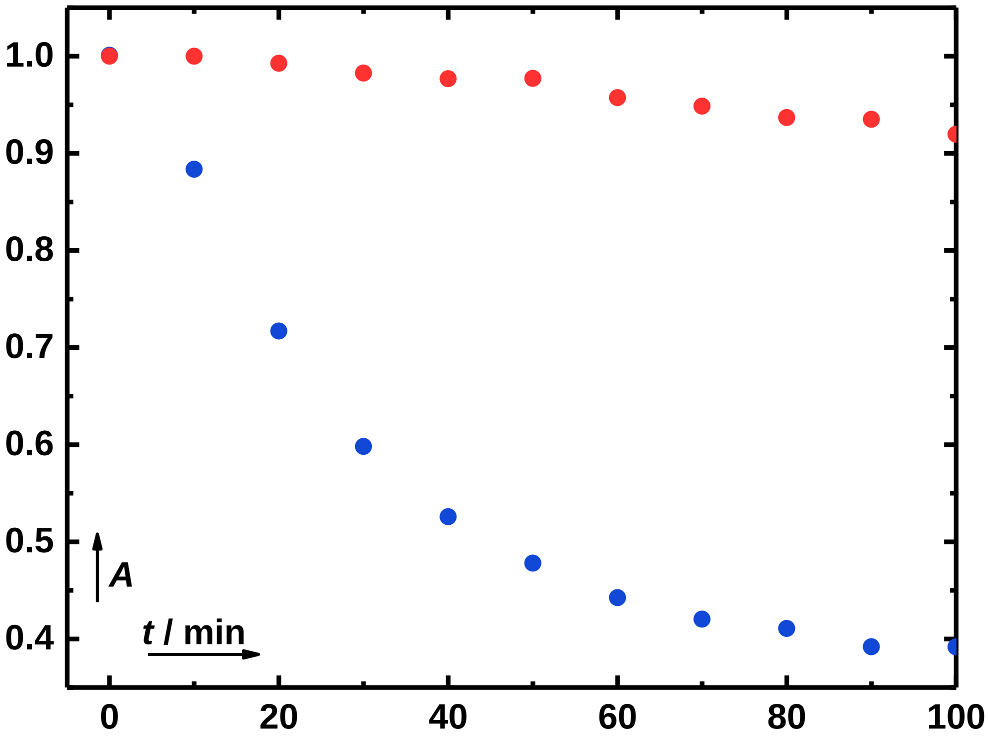

Figure S54. Comparison of kinetic traces measured at λ = 422 nm for **5b** in aerated (blue) and degassed (red) methanol irradiated at 820 nm. Normalized to *A* = 1.0 at *t* = 0 min.

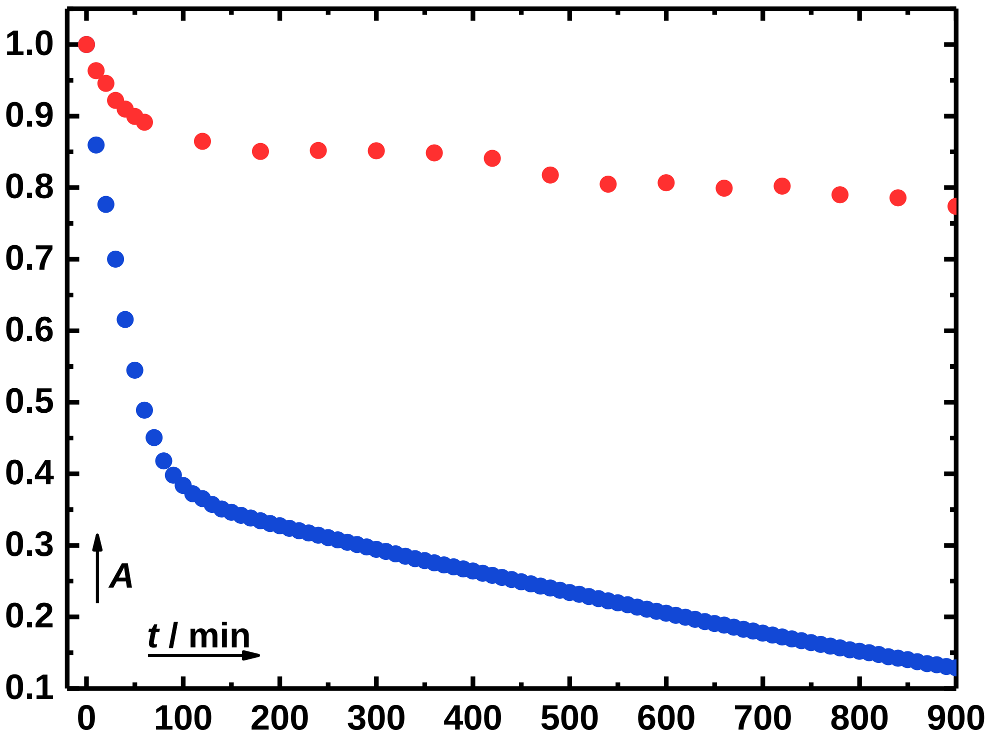

Figure S55. Comparison of kinetic traces measured at λ = 793 nm for **5b** in aerated (blue) and degassed (red) methanol irradiated at 820 nm. Normalized to *A* = 1.0 at *t* = 0 min.

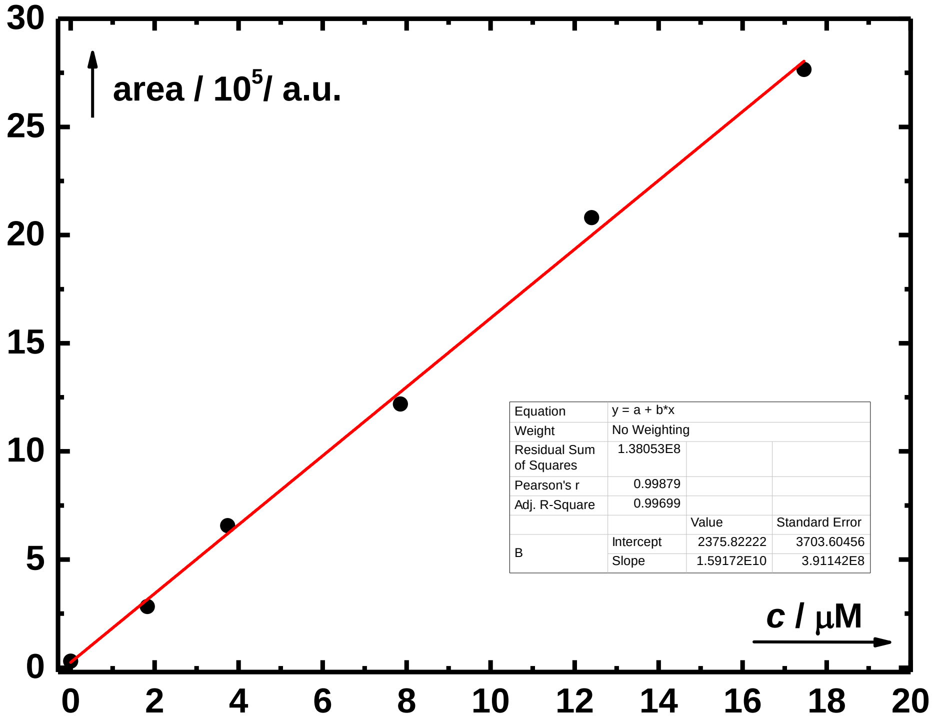

Figure S56. GC-headspace calibration (a 5Å molecular sieve-packed column equipped with a TIC/MS detector in a SIM mode). CO produced by irradiation of cyclopropenone photoCORM[10] at 375 nm and the signal detected at a retention time of 2.96 min.

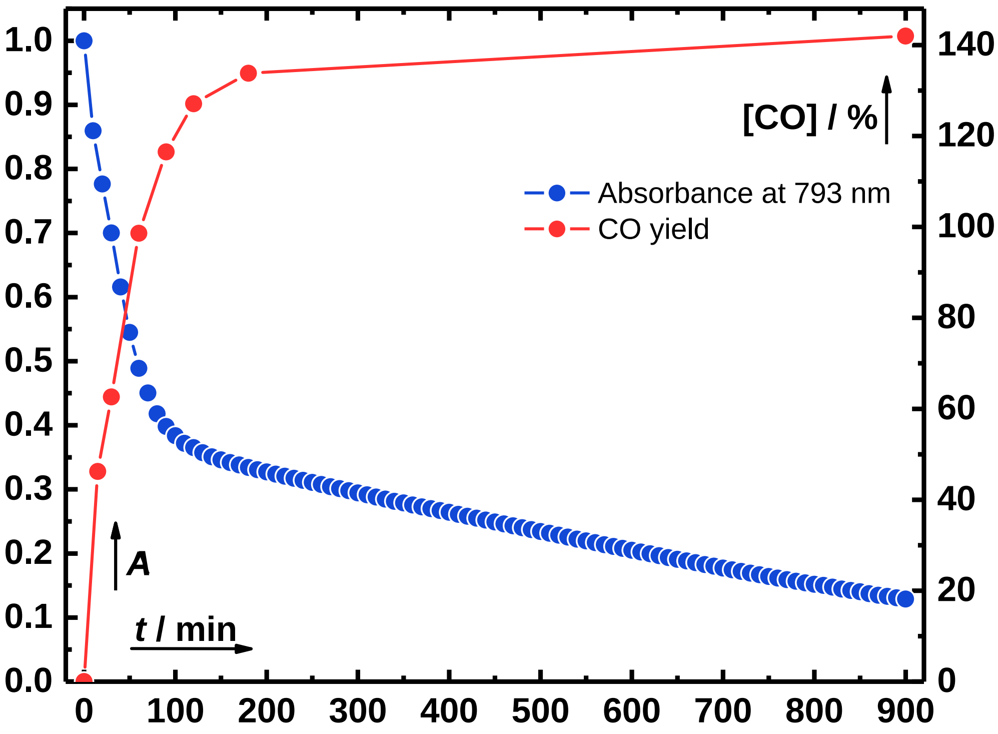

Figure S57. Kinetic trace at measured at λ = 793 nm (blue) and chemical yields of CO (red) produced upon irradiation of **5b** ($c \sim 3.8 \times 10^{-6}$ M) in methanol at 820 nm.

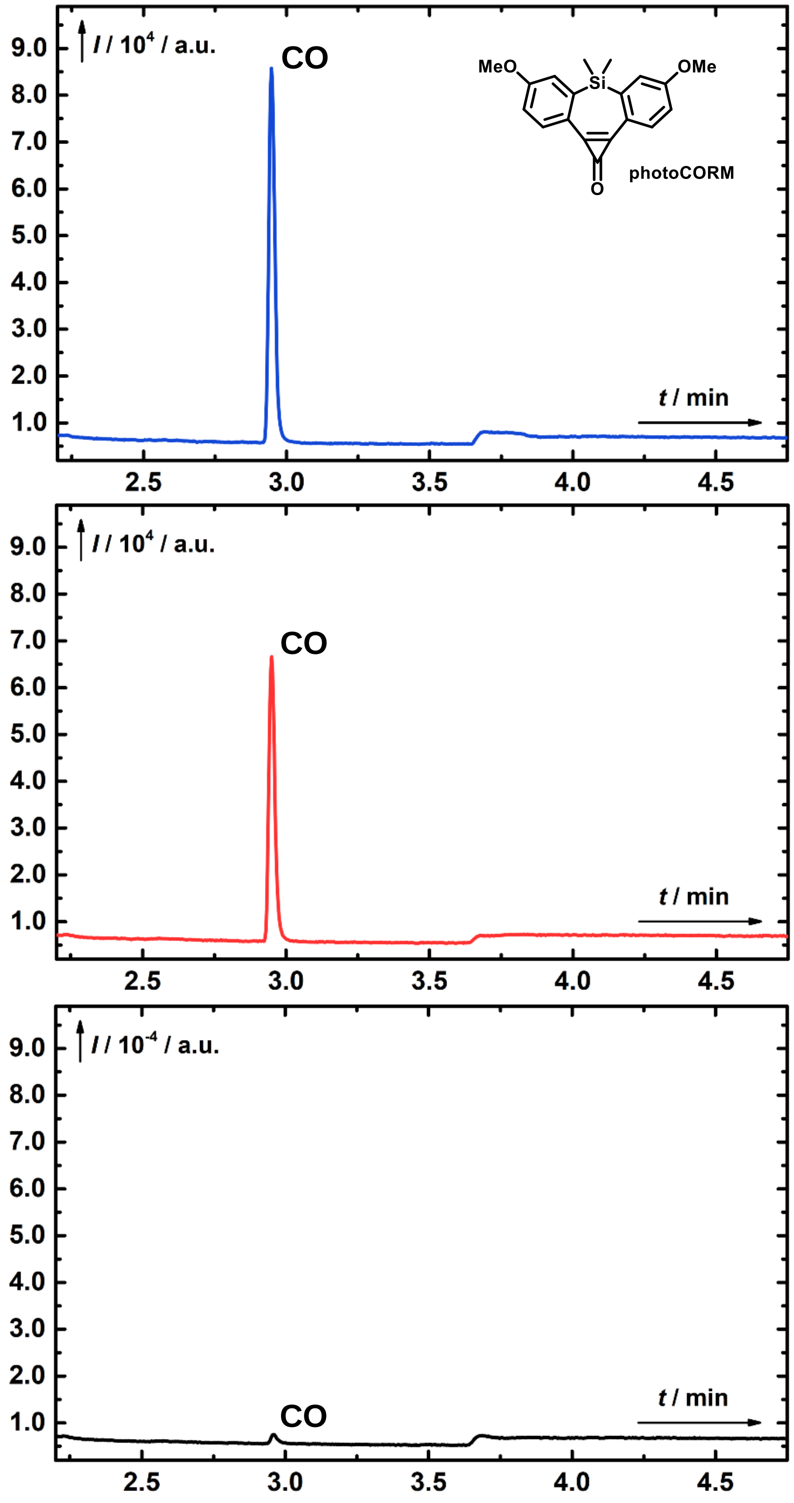

Figure S58. GC-Headspace chromatograms. (red) CO produced upon irradiation of cyclopropenone photoCORM[10] at 375 nm. (blue) CO produced upon irradiation of **5b** (c ~ 3.8 × 10^{-6} M) in methanol at 820 nm for 180 min. (black) CO produced from **5b** (c ~ 3.8 × 10^{-6} M) in methanol in the dark after 16h.

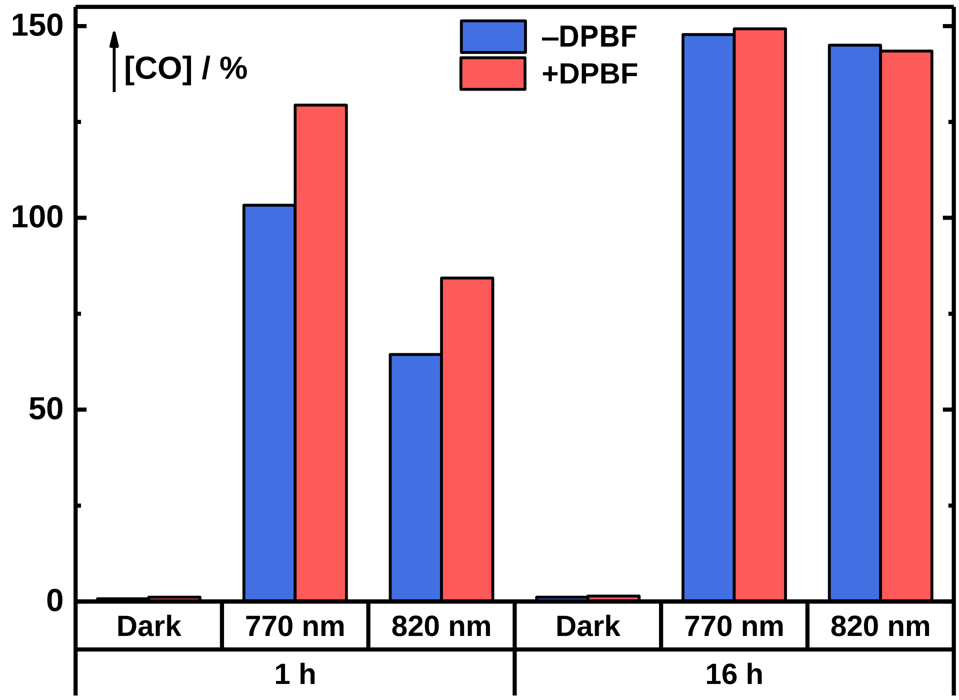

Figure S59. The relative concentrations of CO found in solutions of **5b** (c ~ 5.4 × 10^{-6} M) in aerated methanol in the dark or upon irradiation at 770 nm or 820 nm for 1 and 16 h in the absence (blue) or presence (red) of DPBF (c ~ 8.0 × 10^{-3} M) as a singlet oxygen trap.

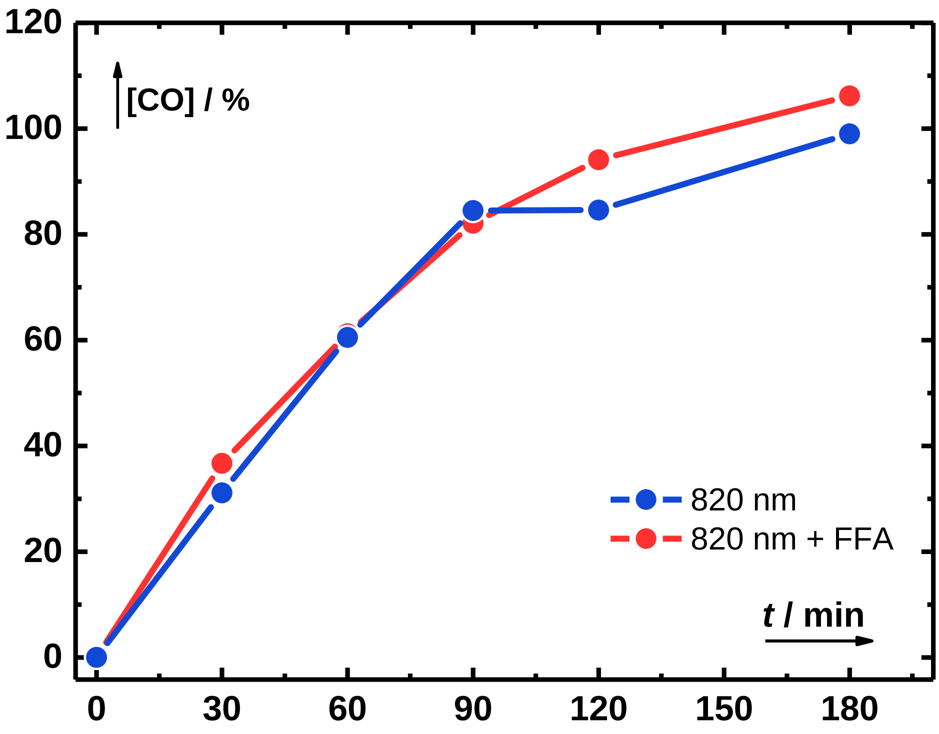

Figure S60. Chemical yields of CO produced upon irradiation of **5b** (c ~ 3.9 × 10^{-6} M) in aerated methanol at 820 nm in the absence (blue) or presence (red) of furfuryl alcohol (c ~ 6.0 × 10^{-3} M) as a singlet oxygen trap.

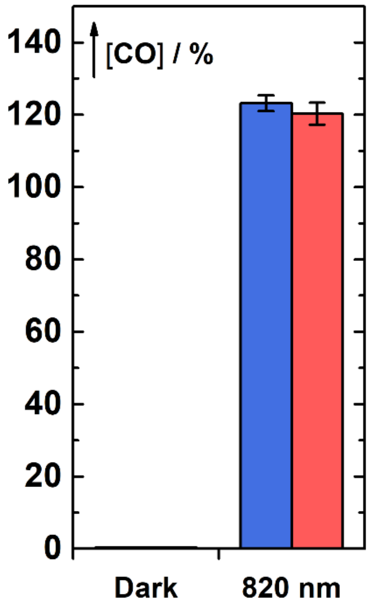

Figure S61. Chemical yields of CO produced in the dark or upon exhaustive irradiation of **5b** (*c* ~ 2.4 × 10^{-5} M) in PBS (pH 7.4, 10 mM, I = 100 mM) at 820 nm in the absence (blue) or presence (red) of bovine serum albumin BSA (*c* = 20 mg mL^{-1}).

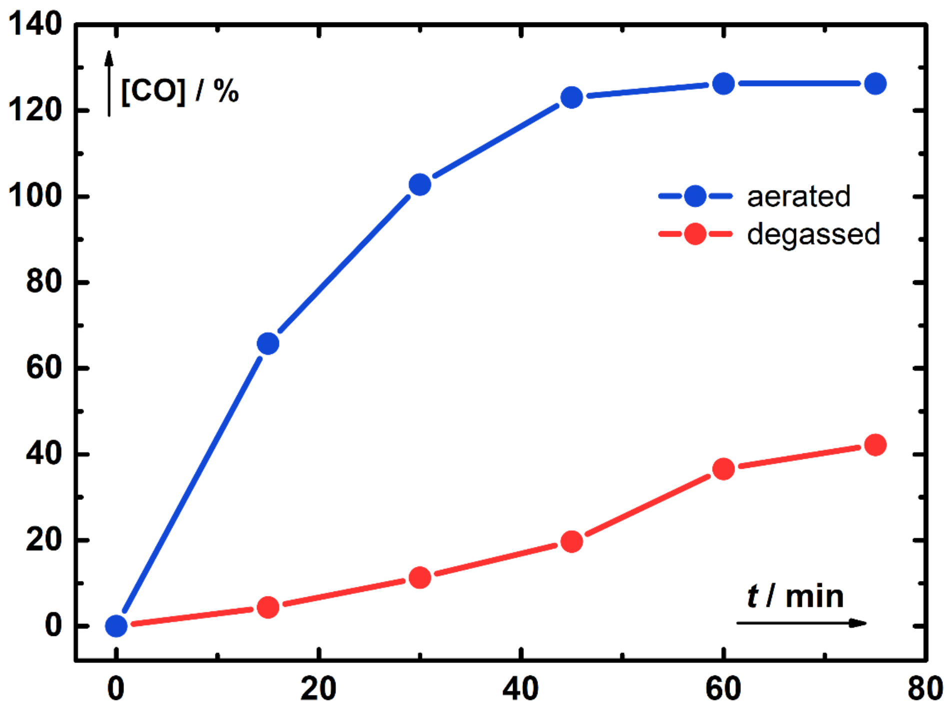

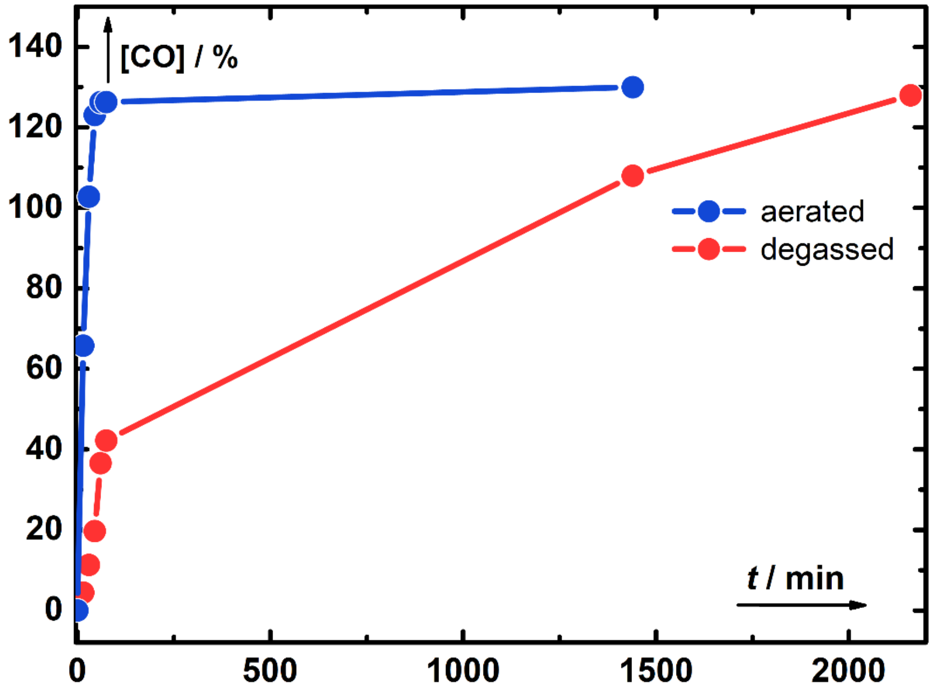

Figure S62. Time-dependent release of CO from **5b** in aerated methanol (blue) or samples prepared in hypoxic conditions (3 ppm O_2; red) ($c \sim 9 \times 10^{-6}$ M) upon irradiation with 770 nm light up to 75 min (top) and up to 36 h (bottom). The CO released was determined by GC–headspace and is expressed as the total chemical yield.

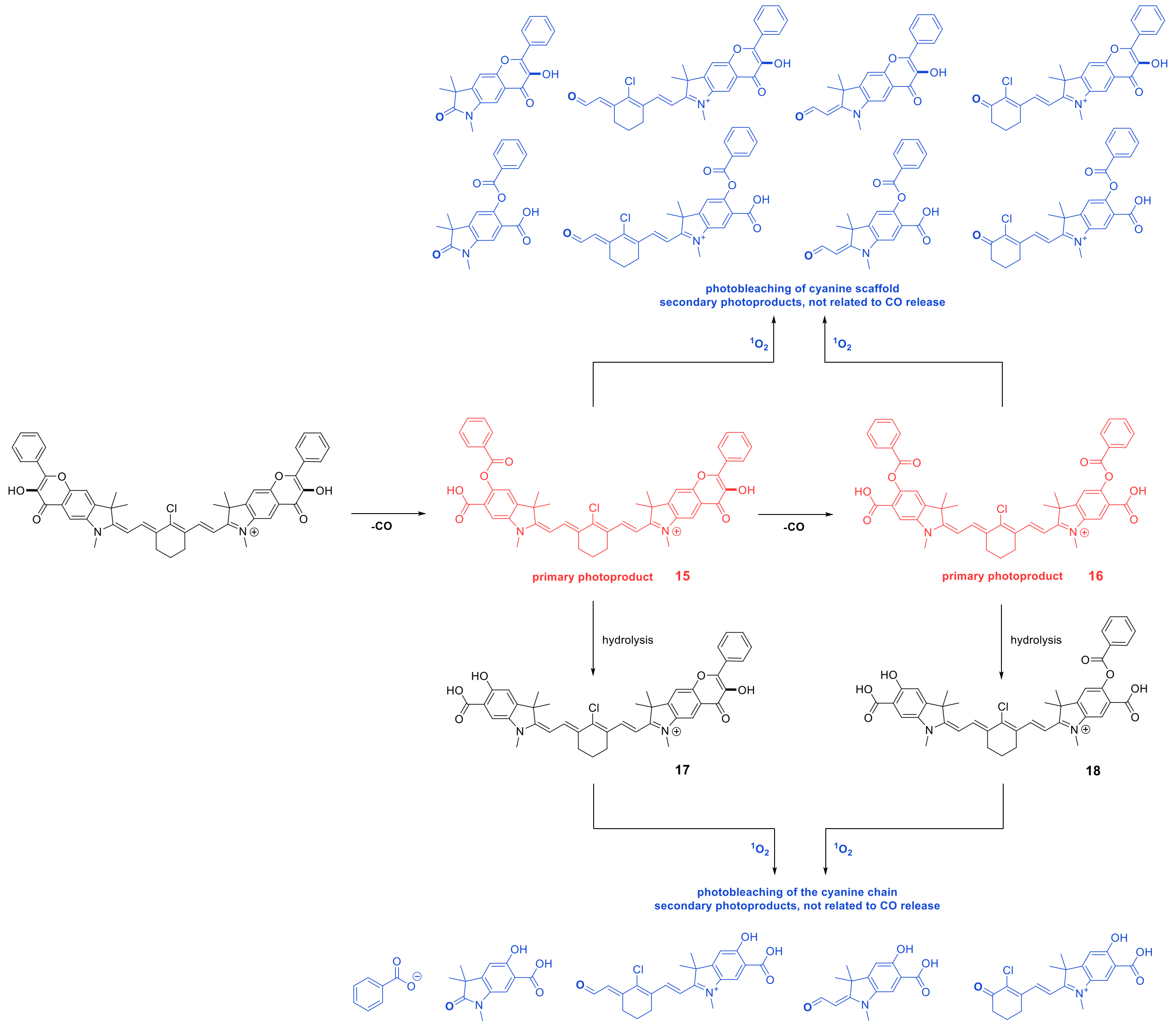

Figure S63. The depiction of photoproducts resulting from the irradiation of **5a**. The primary photoproducts related to the release of CO are depicted in red (**15** and **16**). The products of benzoate ester hydrolysis in the dark are depicted in black (**17** and **18**). The secondary photoproducts, which follow the general photooxygenation pathway of the cyanine scaffold, are depicted in blue.

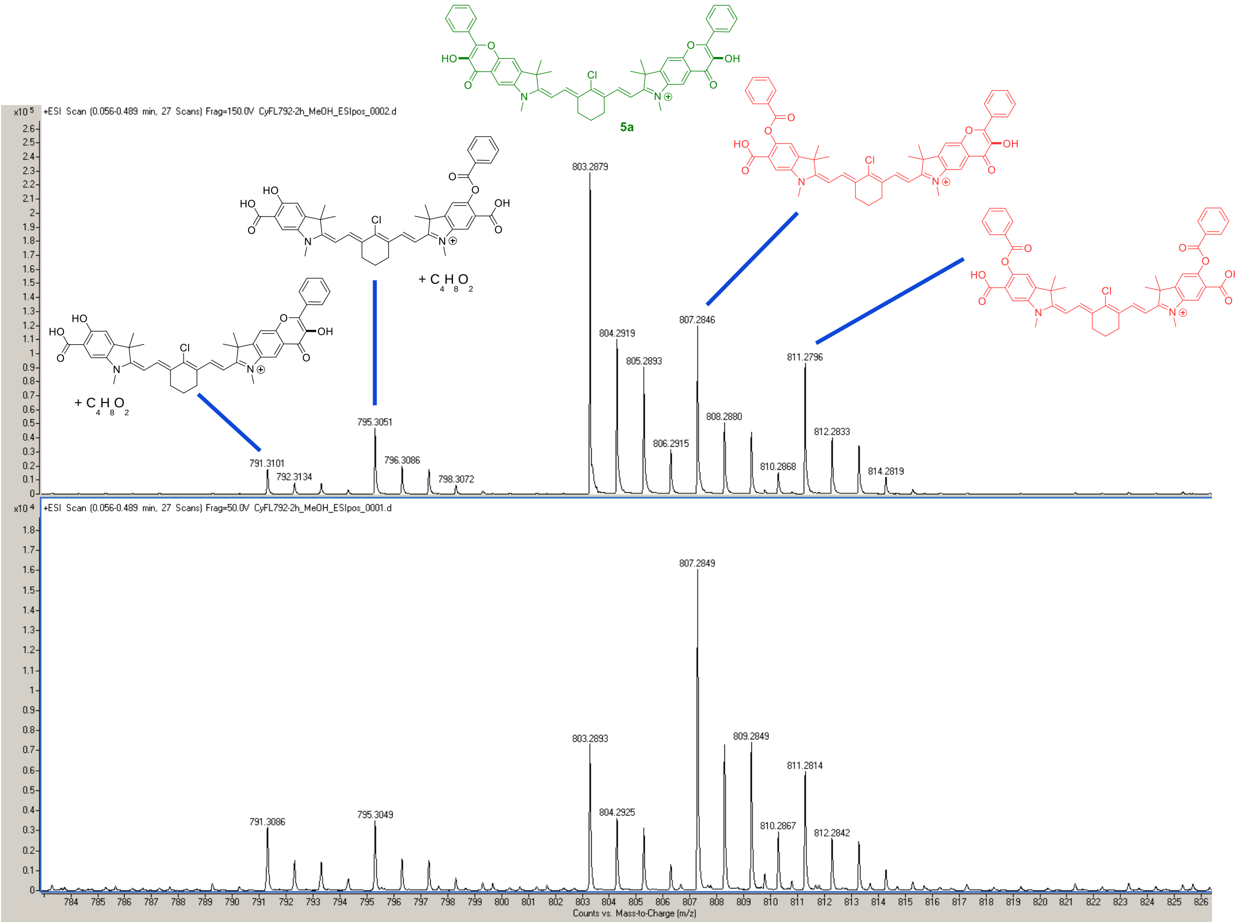

Figure S64. HRMS (ESI+) of **5a** in aerated methanol (c ~ 4 × 10^{-5} M, 1 mm cuvette) irradiated at 820 nm for 2 h (top) and 3 h (bottom). The primary photoproducts of photoinitiated CO release are depicted in red. The products resulting from spontaneous hydrolysis of the benzoate ester are depicted in black.

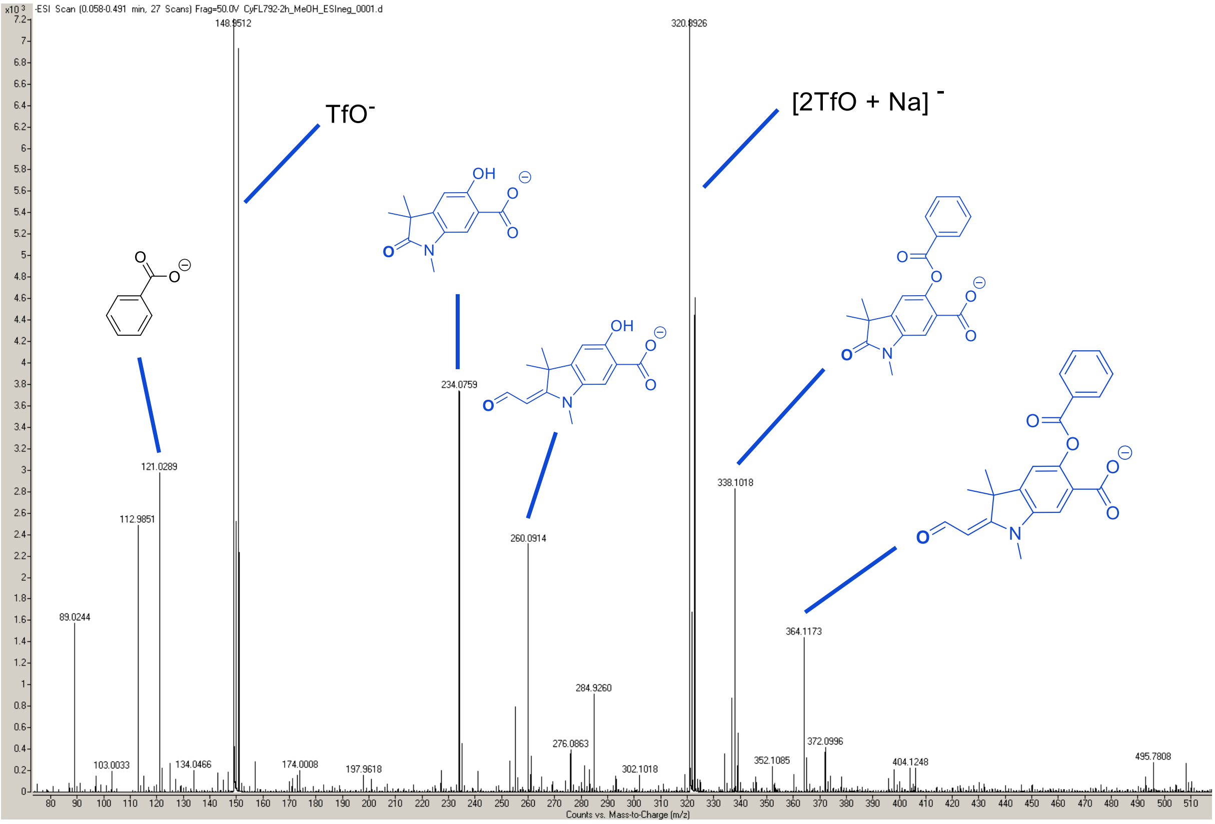

Figure S65. HRMS (ESI–) of **5a** in aerated methanol ($c \sim 4 \times 10^{-5}$ M) irradiated at 820 nm for 20 h. The secondary photoproducts resulting from photodegradation of the cyanine scaffold are depicted in blue. The products resulting from spontaneous hydrolysis of the benzoate ester are depicted in black.

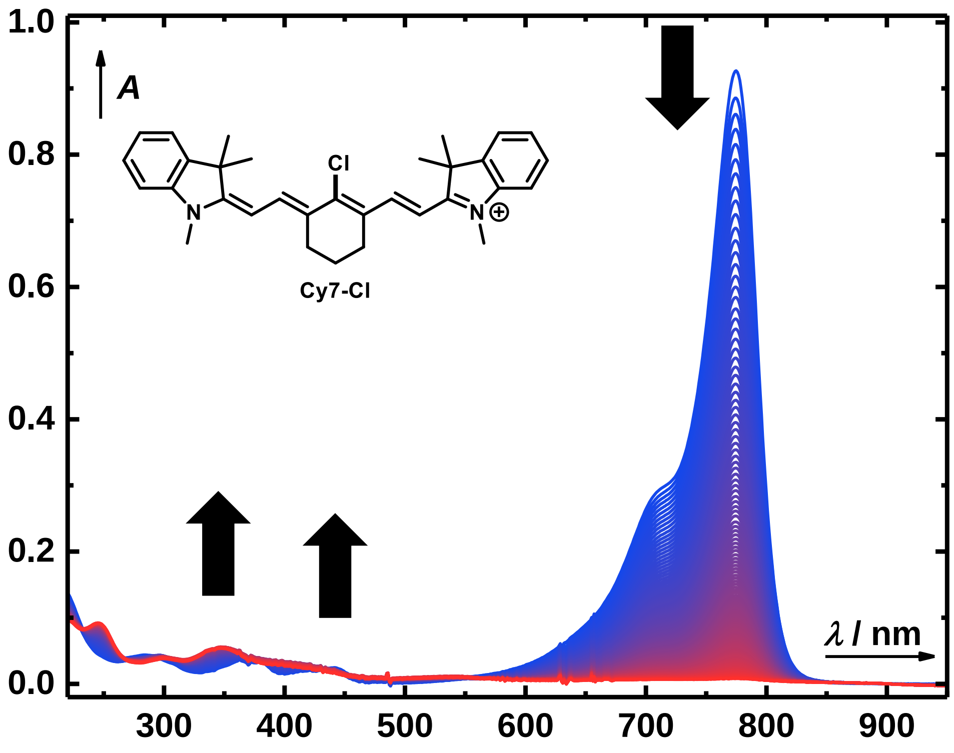

Figure S66. Photobleaching of Cy7-Cl (blue to red) in methanol upon irradiation at 770 nm monitored by UV-vis spectroscopy at 10-min intervals.

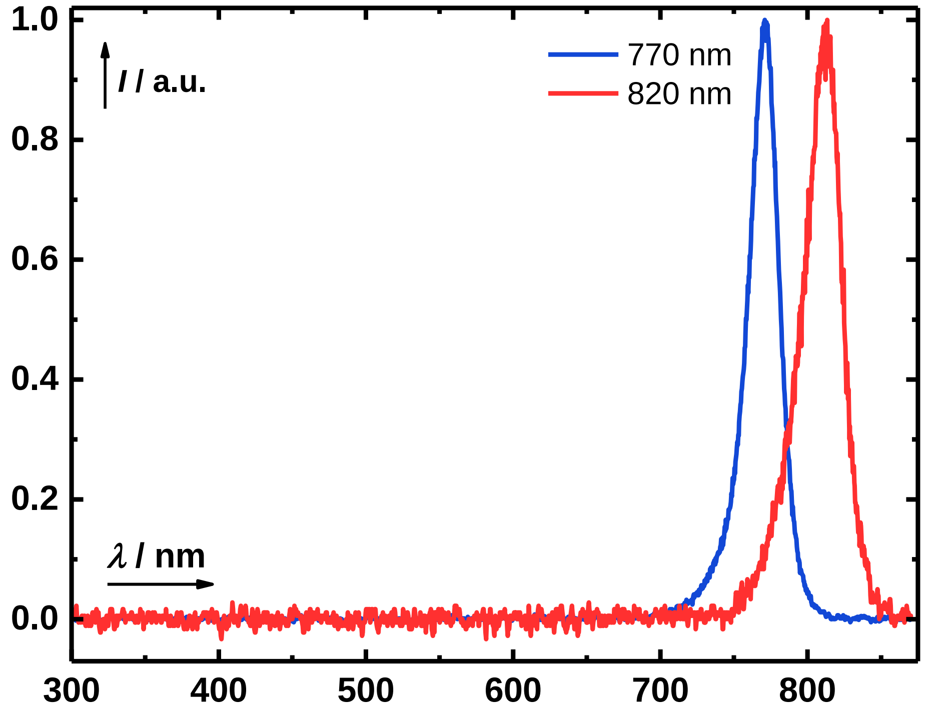

Figure S67. Normalized emission spectra of the LEDs used for irradiation of **5a–b**. λ_{max} = 771 nm (blue solid); λ_{max} = 815 nm (red solid).

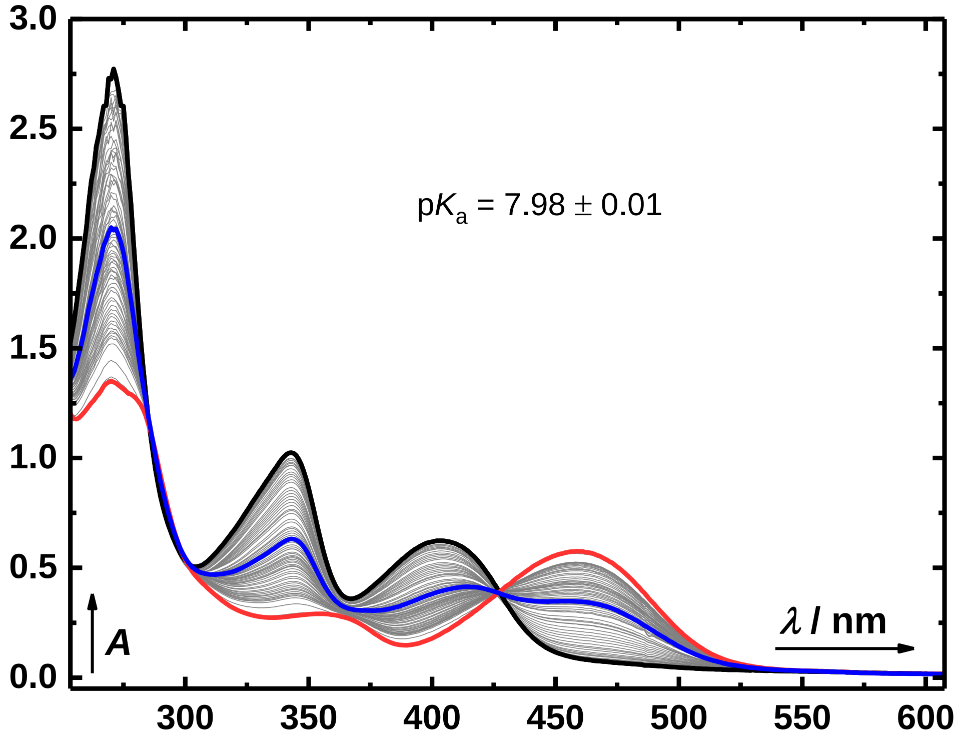

Figure S68. Spectroscopic determination of pK_a of **14** in water. Two distinct absorption bands of **14A** (λ_{max} = 403 nm; black solid) and **14B** (λ_{max} = 459 nm; red solid) with an isosbestic point at λ = 428 nm are shown.

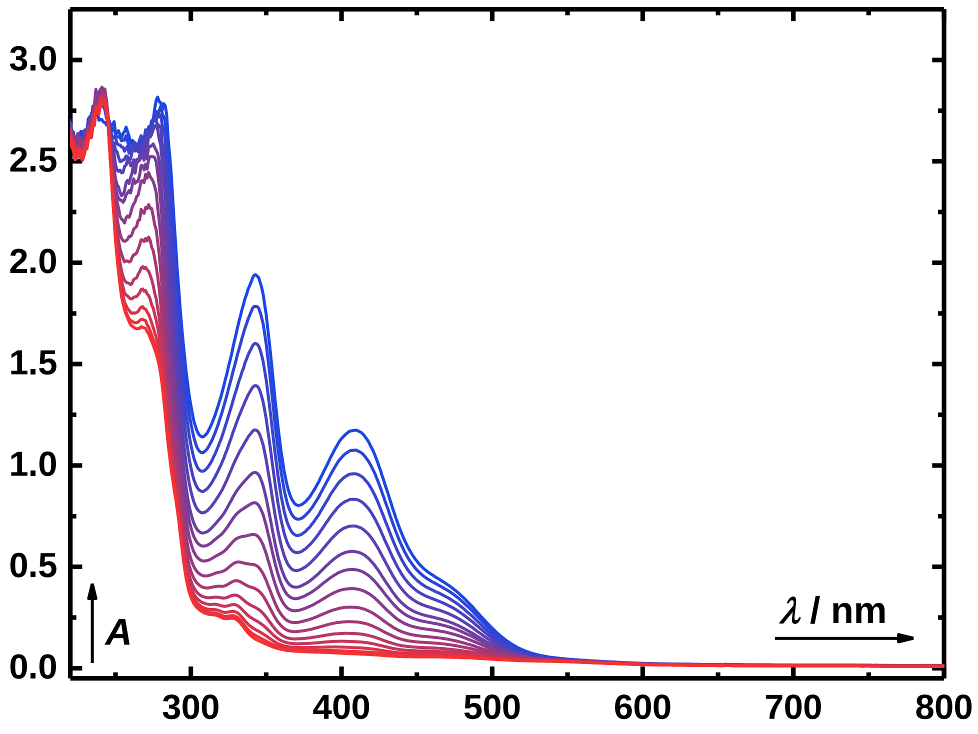

Figure S69. Irradiation of **14** in PBS (pH 7.4, 10 mM, I = 100 mM) at 405 nm followed by UV-vis spectroscopy at 1-min intervals (from blue to red).

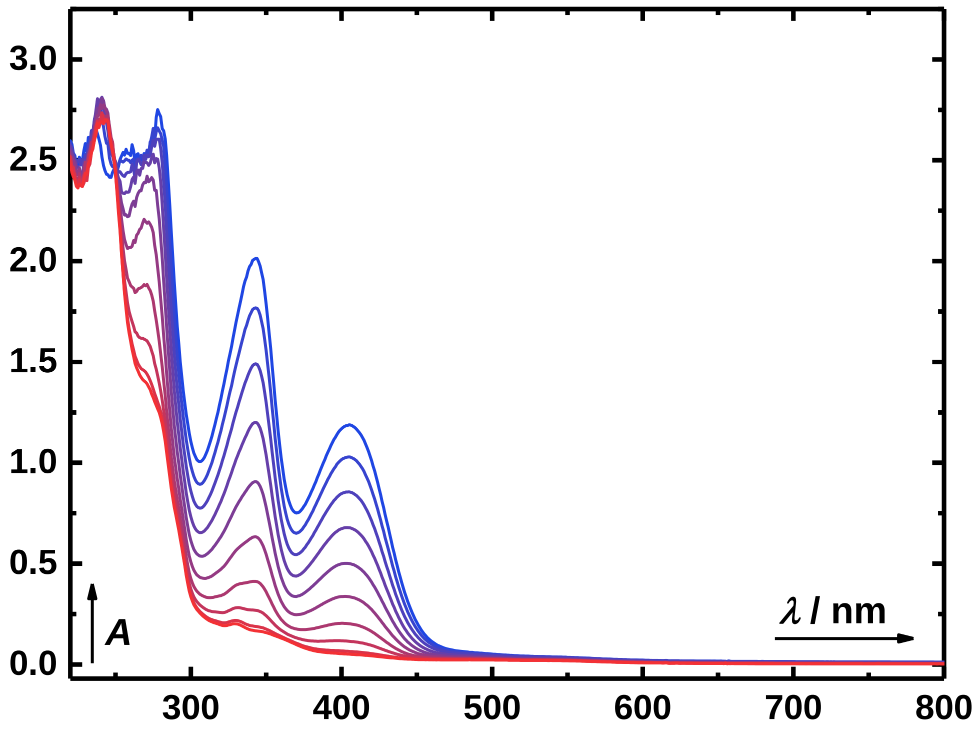

Figure S70. Irradiation of **14** in PBS (pH 5.0, 10 mM, I = 100 mM) at 405 nm followed by UV-vis spectroscopy at 2-min intervals (from blue to red).

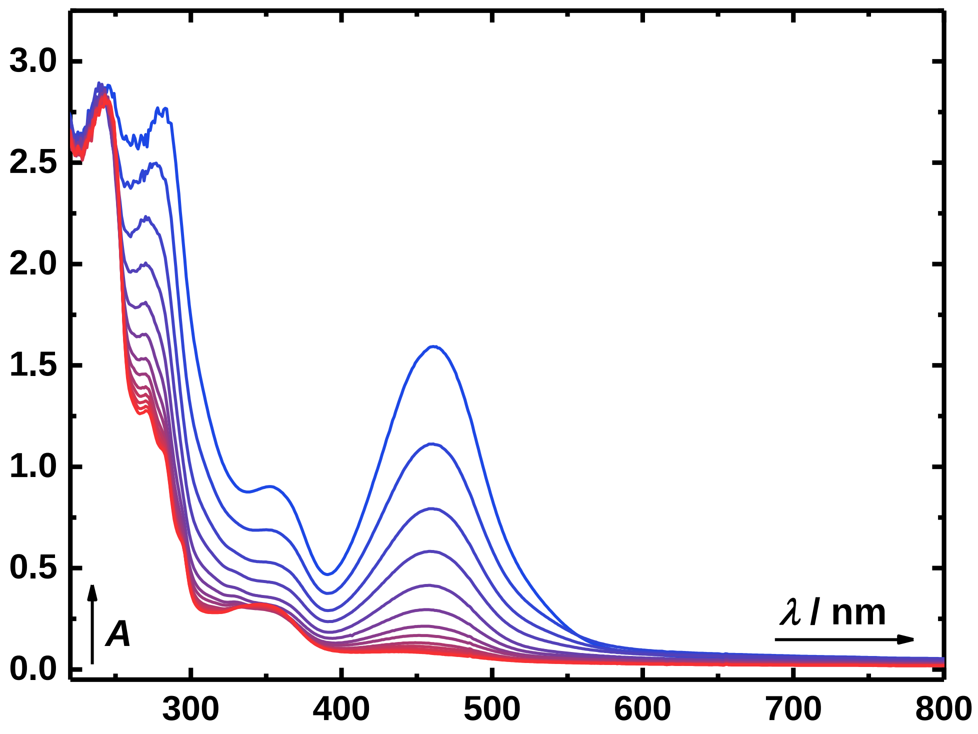

Figure S71. Irradiation of **14** in glycine buffer (pH 9.8, 10 mM) at 465 nm followed by UV-vis spectroscopy at 30-s intervals (from blue to red).

Competition Between CO Release and Cyanine Photooxygenation

The rate of photooxygenation of cyanine **4** can be expressed as:

$$k_{\mathrm{r}} = -\frac{d[\mathbf{4}]}{dt} = \frac{dn(\mathbf{4})}{V\,dt} = k_{\Sigma}\,[^{1}\mathrm{O}_2][\mathbf{4}] \qquad \text{(Eq. S4)}$$

where k_r is the rate of decomposition, [**4**] is the concentration of **4**, n(**4**) is the amount of cyanine **4** in moles, V is the sample volume, k_{Σ} is the bimolecular reaction rate of **4** and singlet oxygen, and $[^1O_2]$ is the concentration of singlet oxygen.

The quantum yield of polyene chain photooxygenation is defined by:

$$\Phi_{\mathrm{dec}} = \frac{dn(\mathbf{4})}{q_{n,\mathrm{p}}^{0}\,[1-10^{-A(\lambda)}]dt} \qquad \text{(Eq. S5)}$$

where $q_{n,\mathrm{p}}^{0}$ is the spectral photon flux of incident photons, and $A(\lambda)$ is the absorbance of the sample at the given wavelength. Combining Eq. S1 and Eq. S2 gives:

$$\Phi_{\mathrm{dec}} = \frac{k_{\mathrm{r}}[^{1}\mathrm{O}_2][\mathbf{4}]\,V}{q_{n,\mathrm{p}}^{0}\,[1-10^{-A(\lambda)}]} \qquad \text{(Eq. S6)}$$

The production and deactivation of singlet oxygen is given by the following rate law:

$$\frac{d[^{1}\mathrm{O}_2]}{dt} = \frac{\Phi_{\Delta}\,q_{n,\mathrm{p}}^{0}\,[1-10^{-A(\lambda)}]}{V} - k_{\mathrm{d}}[^{1}\mathrm{O}_2] - k_{\Sigma}\,[^{1}\mathrm{O}_2][\mathbf{4}] \qquad \text{(Eq. S7)}$$

where Φ_{Δ} is the known quantum yield of singlet oxygen formation of **4** and k_d is the first-order rate constant of the quenching by a solvent. Applying steady-state approximation for $[^1O_2]$, we get:

$$[^{1}\mathrm{O}_2] = \frac{q_{n,\mathrm{p}}^{0}\,[1-10^{-\mathrm{A}(\lambda)}]}{V} \times \frac{\Phi_{\Delta}}{(k_{\mathrm{d}} + k_{\Sigma}[\mathbf{4}])} \qquad \text{(Eq. S8)}$$

Finally, plugging Eq. S5 to Eq. S3 gives:

$$\Phi_{\mathrm{dec}} = \frac{k_{\Sigma}[\mathbf{4}]\Phi_{\Delta}}{k_{\mathrm{d}} + k_{\Sigma}[\mathbf{4}]} \qquad \text{(Eq. S9)}$$

Employing the values used in the UV-vis spectroscopy experiments ([**4**] = 3.8×10^{-6} M; k_{Σ} = 1.7×10^{7} M^{-1} s^{-1}; $k_d = 9 \times 10^4$ s^{-1} and $\Phi_{\Delta} = 0.0034$ and 0.0062 for **5a** and **5b**, respectively), the calculated values of $\Phi_{dec} = 2.4 \times 10^{-6}$ and 4.5×10^{-6} are obtained for **5a** and **5b**, respectively. Hence, the ratio of Φ_{CO} and Φ_{dec} is calculated (Eq. S7), consistent with our observations by UV-vis spectroscopy:

$$\mathbf{5a}: \frac{\Phi_{\mathrm{CO}}}{\Phi_{\mathrm{dec}}} = \frac{2.0\times10^{-4}}{2.4\times10^{-6}} = 83 \qquad \mathbf{5b}: \frac{\Phi_{\mathrm{CO}}}{\Phi_{\mathrm{dec}}} = \frac{3.0\times10^{-4}}{4.5\times10^{-6}} = 67 \qquad \text{(Eq. S10)}$$

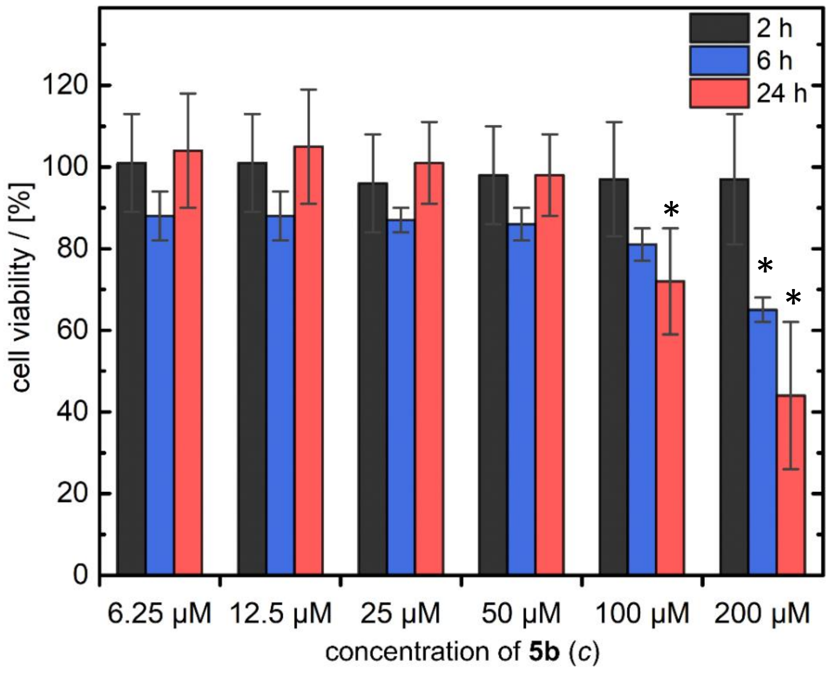

Figure S72. Effects of **5b** at different concentrations (*c*) on the viability of HepG2 cells. The cells were incubated in dark with **5b** for 2, 6 or 24 h. Viability was measured using an MTT assay. The experiments were performed in triplicates. The data are expressed as a mean ± standard deviation. * $P < 0.05$

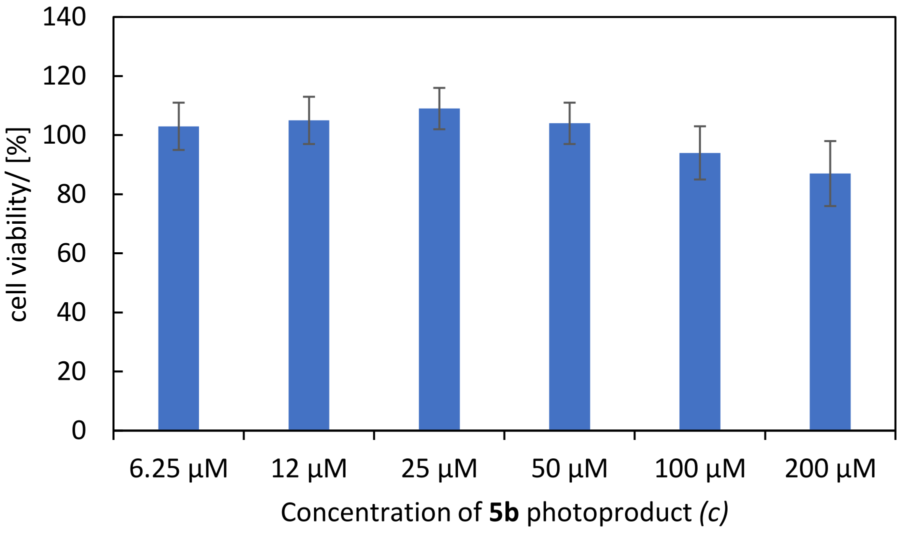

Figure S73. Effect of 5b photoproduct on the viability of HepG2 cells. The photoproduct was produced by irradiation of **5b** at λ = 780 nm for 24 h in the absence of cells. The cells were incubated with photoproduct for 24 h at different concentrations (*c*). Viability was measured using MTT assay. The experiments were performed in triplicates. Data expressed as a mean ± standard deviation.

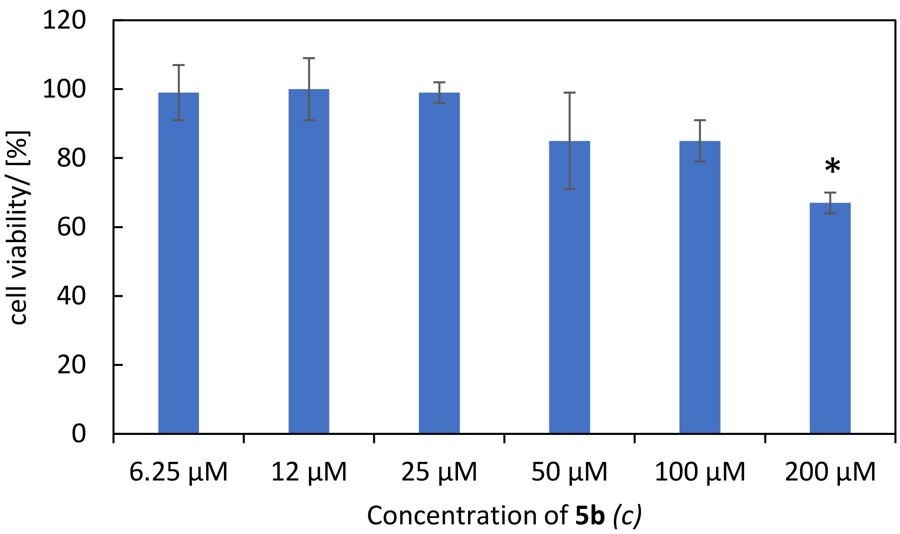

Figure S74. Effects of **5b** irradiation on the viability of HepG2 cells. HepG2 cells were irradiated with different concentrations of **5b** at λ = 780 nm for 1 h. Following consecutive 24 h incubation without irradiation, cell viability was measured using MTT assay. The experiments were performed in triplicates. Data expressed as a mean ± standard deviation. * $P < 0.05$
